# Stereoconvergent Chain-Growth Polymerization

**DOI:** 10.1021/acscentsci.5c00239

**Published:** 2025-05-05

**Authors:** Jake R. Jagannathan, Frank A. Leibfarth

**Affiliations:** Department of Chemistry, 214885 University of North Carolina at Chapel Hill, Chapel Hill, North Carolina 27514, United States

## Abstract

The stereochemistry of polymers has a profound impact on their properties. Despite the well-developed stereoselective methods for prochiral vinyl monomers, current methods for racemic monomers are limited. Conventional approaches treat *sp* ^*3*^ chiral centers as immutable, resulting in poor atom-economical processes and limited control over enantioselectivity. This contrasts with stereoconvergent catalysis in small molecules, which has revolutionized synthesis by interrupting the transfer of chiral information from the substrate to the product, providing a clear platform for catalysts to access enantiopure compounds from racemic mixtures in up to 100% yield. Here we designed a catalyst that converges stereochemical information during polymerization, enabling access to asymmetric, isotactic polymers with quantitative atom economy from racemic feedstocks. The mechanism of stereoconvergence is accomplished by the catalyst ablating chiral information, followed by a stereoselective propagation event to control both tacticity and enantioselectivity. Using this method, we accessed both enantiomers of an isotactic polymer from a single enantiomer of monomer and identified a novel stereocomplex. These results represent a conceptual framework to expand stereoconvergent polymerization into additional monomers and mechanisms.

## Graphical abstract

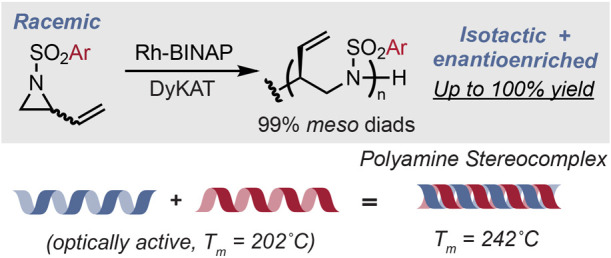

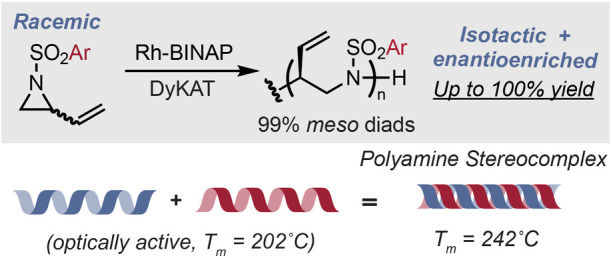

## Introduction

Developing methods to control the relative (tacticity) and absolute (asymmetry) stereochemistry of polymers is a well-known approach to access emergent properties from simple building blocks. As an exemplar, the stereoselective coordination–insertion polymerization of propylene to isotactic polypropylene provides tough semicrystalline plastics, representing the largest volume application of stereoselective catalysis at >50 million metric tons annually.^1–3^ Control over asymmetry^4–8^ provides orthogonal tuning of optical, biological, or supramolecular activity when repeat units lack pseudosymmetry, exemplified with advances in luminescent materials and polymer stereocomplexes.^9–12^ Coordination–insertion polymerization is highly effective for stereoselective vinyl polymerization, which enchains prochiral monomers into a macromolecule and simultaneously sets *sp* ^*3*^ chiral centers in the backbone.^13,14^ Although successful for controlling the tacticity of polyolefins, coordination–insertion polymerization translates poorly to monomers with heteroatoms due to catalyst poisoning.^15–17^ This fundamental limitation represents an impediment toward the synthesis of stereodefined materials from sustainable building blocks.^6,18^

There have been several foundational approaches to controlling stereochemistry with heteroatom-rich monomers. Modern petrochemically or biologically derived monomers often contain preinstalled *sp* ^*3*^ chiral centers in racemic form, which leads to atactic, racemic polymers in the absence of a catalyst that biases stereochemistry.^19–21^ Recent advances in isoselective polymerizations^22–24^ or accessing chiral pool feedstocks^25,26^ enable control of tacticity and enantioselectivity, respectively, leading to materials with valuable properties. However, simultaneous control of both tacticity and enantioselectivity starting from a racemic monomer remains an unsolved challenge. To access isotactic, asymmetric polymers from these racemic mixtures, asymmetric kinetic resolution polymerization (AKRP) is the state-of-the-art approach, where a chiral catalyst interacts preferentially with one monomer enantiomer and polymerizes it at a relatively faster rate than the opposite enantiomer (Figure 1A). While effective, an ideal AKRP with perfect selectivity demonstrates poor atom economy because the yield of an isotactic, enantiopure polymer is 50%. Subsequent propagations enchain the less reactive monomer, leading to a stereoblock polymer and a loss of asymmetry control because the polymer now contains both *R* and *S* stereocenters. Moreover, current catalysts cannot edit absolute stereochemistry prior to propagation, limiting the control catalysts can have during polymerization.^27^ While the resultant materials may retain valuable thermomechanical properties, these limitations can have deleterious consequences in applications such as biotechnology and optics where slight changes in asymmetry alter material function.

**Figure 1:**
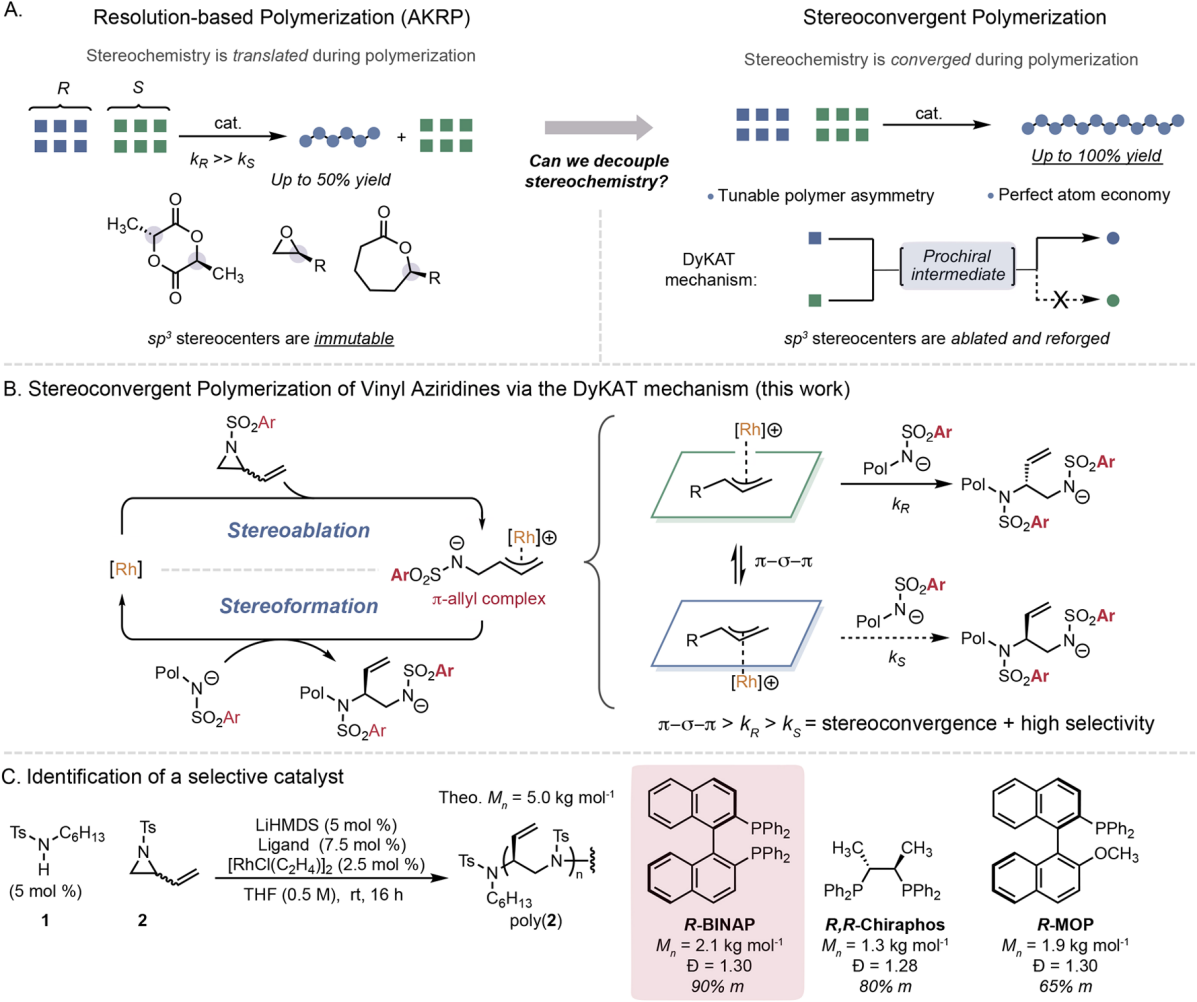
Stereocontrolled polymer synthesis with chiral monomers. (A) Asymmetric kinetic resolution polymerization is the state-of-the-art to achieve isotactic polymers from racemic monomers. (B) Stereoconvergent polymerization via the dynamic kinetic asymmetric transformation (DyKAT) mechanism. (C) Identification of a lead catalyst (R = generic substituent; cat. = catalyst; Ar = aryl group; LiHMDS = lithium hexamethyldisilazane; THF = tetrahydrofuran; Ts = tosyl; rt = room temperature).

The key limiting assumption of AKRP is that stereochemistry is immutable; the stereochemistry of the monomer translates to the stereochemistry of the polymer. Work in asymmetric catalysis, however, demonstrates that stereochemistry can be edited throughout the course of a reaction to improve the atom economy. Approaches take advantage of either the reversible racemization of the starting materials before a stereodetermining reaction (i.e., dynamic kinetic resolution, DKR) or a stereoablative reaction step to yield a prochiral intermediate that can be resolved through a facial selective reaction (i.e., dynamic kinetic asymmetric transformation, DyKAT) (Figure 1A).^28–30^ Despite the substantial precedent in small-molecule catalysis, the challenges inherent to chain-growth polymerization, including substoichiometric concentrations of reactive species and iterative reaction steps under triple diastereocontrol, have limited progress.^31^ Previously, stereoconvergent oligomerization has been advanced using a DKR approach with a two-catalyst system where one catalyst racemized a chiral chain-end and the other catalyst performed AKRP.^32,33^ Despite the fundamental advancement, the catalysts exhibited compatibility issues,^34^ which limited the isotactic oligomers to low molar masses (<3.0 kg mol^–1^).

We identified allylic amination of vinyl aziridines via prochiral transition-metal π-allyl complexes as an attractive transformation that could translate into a stereoconvergent polymerization (Figure 1B).^35–43^ We hypothesized that a DyKAT mechanism, where a single moiety both catalyzes polymerization and converges stereochemistry, is better suited for the development of stereoconvergent polymerizations to access well-defined, high molar mass polymers. In allylic amination, the initial stereochemistry is ablated and then reforged to enable incorporation of both enantiomers into an isotactic, enantioenriched polymer with quantitative atom economy. Furthermore, desymmetrization of the prochiral π-allyl complex provides a pathway to independently control tacticity and enantioselectivity through catalyst design. The resultant polyamine materials would enable systematic evaluations of tacticity and asymmetry control, with potential applications as biomaterials and adhesives.^41–43^

## Design of the Polymerization

The proposed catalytic cycle begins with a chiral metal catalyst performing stereoablative oxidative addition into the monomer to generate the prochiral π-allyl intermediate (Figure 1B). Attack of the chain-end or initiator to access the chiral regioisomer then regenerates the catalyst. Tacticity and enantioselectivity are controlled by a chiral ligand that differentiates the rates of enantiofacial nucleophilic addition to the π-allyl complex. Unsymmetrical π-allyl complexes are known to interconvert between **Pro**-*R* and **Pro**-*S* faces via π–σ–π isomerization, which serves as the fulcrum for stereoconvergence.^36,44,45^ When π–σ–π isomerization is faster than propagation, we hypothesized that a racemic monomer pool can converge to an enantioenriched, isotactic polymer under chain-growth conditions (Figure 1B).

A model system using sulfonamide **1** and aziridine **2** as initiator and monomer, respectively, was selected to develop a stereoconvergent chain-growth polymerization (Figure 1C). The addition of lithium hexamethyldisilazane base (LiHMDS) was needed to generate a nucleophilic chain-end to promote propagation.^40^ Screening polymerizations with a target number-average molar mass (*M*_*n*_) of 5.0 kg mol^–1^ with several metals^44,46,47^ identified Rh­(I) with the chloride counterion as competent to produce poly­(**2**) with *M*_*n*_ = 1.9 kg mol^–1^ and dispersity (*Đ*) of 1.38 as a single regioisomer containing a chiral center in the repeat unit (Table S1). Rhodium catalysts are known to exhibit regiochemical memory effects in allylic alkylation due to formation of enyl complexes.^47–49^ Analysis of poly­(**2**) via ^13^C NMR spectroscopy revealed 54% *meso* diads (% *m*), indicating an atactic polymer in the absence of a chiral ligand (Figure 2B). Chiral ligands were subsequently employed to bias facial addition to the π-allyl intermediate and engender stereoselectivity (Table S2). The polymerization using *R*-BINAP resulted in precipitation of an insoluble powder that did not dissolve in typical solvents for size exclusion chromatography (SEC). We hypothesized that the product may be isotactic poly­(**2**), which was confirmed by ^13^C NMR spectroscopy to have 90% *m* diads (Figure S2). Both the binaphthyl and bisphosphine components were necessary for high selectivity, as evident with *R,R*-chiraphos and *R*-MOP producing poly­(**2**) with 80% and 65% *m*, respectively (Figure 1C).

**Figure 2:**
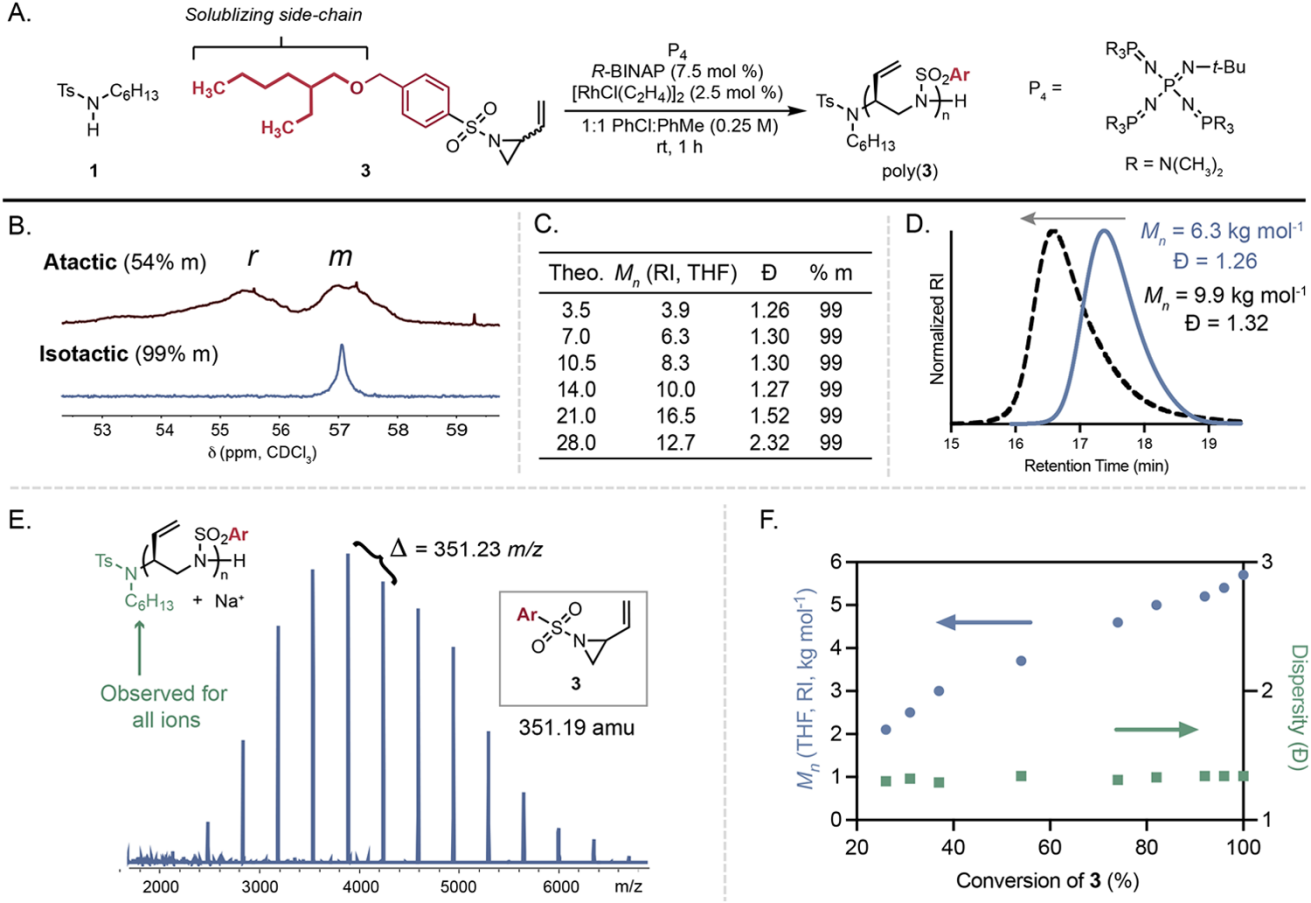
Stereoconvergent polymerization to access well-defined, isotactic polymers. (A) Optimized polymerization conditions with **3**. (B) ^13^C NMR spectra in CDCl_3_ of atactic (top) and isotactic (bottom) poly­(**3**). (C) Tuning molar mass with reduced loading of **1**; SEC (kg mol^–1^) was performed in THF in comparison to polystyrene calibrants. (D) Chain-extension of poly­(**3**). (E) MALDI-TOF MS spectrum of poly­(**3**). (F) *M*_*n*_ and *Đ* vs conversion. Theo. = theoretical; RI = refractive index.

To overcome the solubility challenges with poly­(**2**), monomer **3** containing a solubilizing *rac*-2-ethylhexyloxy group was synthesized and produced soluble polymers for the proceeding experiments (Figure 2A). Polymerization of **3** with *R*-BINAP targeting an *M*_*n*_ of 7.0 kg mol^–1^ produced poly­(**3**) with 99% *m*, an *M*_*n*_ = 4.8 kg mol^–1^, and a *Đ* = 1.30 (Figure 2B). Several phosphines such as H8-BINAP, *p*-tolyl-BINAP, SEGPHOS, and Difluorphos failed to produce poly­(**3**) with comparable molar mass or *meso* selectivity, solidifying *R*-BINAP as the preferred ligand (Table S3). Small-molecule ring-opened side-product formation was found to be the primary cause of reduced molar mass, which prompted the assessment of solvent and base identity to increase the rate of propagation (Figure S3). The use of a PhCl:PhMe cosolvent system improved the molar mass to 5.3 kg mol^–1^ with identical dispersity and maintained 99% *m* diads (Table S4). Evaluation of the base identity revealed P_4_-phosphazenes as preferred for achieving the target molar mass, which produced poly­(**3**) with *M*_*n*_ = 6.3 kg mol^–1^, *Đ* = 1.30, and 99*% m* with minimal side-product formation (Table S5, Figure 2C, entry 2).

We proceeded to tune the molar mass to evaluate the efficiency of leveraging rhodium π-allyl complexes for chain-growth polymerizations. Polymerizations targeting poly­(**3**) between 3.5 and 28.0 kg mol^–1^ produced materials that eluted at different retention times via SEC, indicative of a controlled polymerization that enables tuning of the molar mass. We calculated a maximum *M*_*n*_ of 16.5 kg mol^–1^ as determined in THF with a refractive index detector (Figure 2C and Figure S6). We observed a reduction in *M*_*n*_ and a loss of control when targeting 28 kg mol^–1^, which we attribute to initiator-free oligomerization that produces poly­(**3**) with *M*_*n*_ = 5.0 kg mol^–1^ and *Đ* = 1.35 (Figures S7 and S8).^40,50^ As initiator loading decreased, a near constant 99% *m* of the resultant poly­(**3**)­s was maintained, suggesting that selectivity remains constant through the reaction (Figure S9). To determine the chain-end fidelity of the polymerization, poly­(**3**) was used as a macroinitiator, and chain-extension was successful to produce higher molar mass materials while maintaining high isotacticity (Figure 2D and Figure S13). Evidence of a chain-growth mechanism of propagation is observed with several techniques. Characterization of poly­(**3**) with matrix-assisted laser desorption ionization time-of-flight mass spectrometry (MALDI-TOF MS) resulted in a single distribution of ions where the mass of **1** is observed on each chain, supporting a single initiation during polymerization (Figure 2E). Spacing between ions was 351.28 *m*/*z*, equal to the exact mass of **3**. Further evidence for chain-growth behavior consistent with a controlled polymerization was found by monitoring the polymerization over time. Linear increases in molar mass are observed with increasing conversion, while dispersity stayed at a near constant 1.3 (Figure 2F).

While ^13^C NMR demonstrated highly isotactic polymers, evaluations with nonlinear optical techniques such as circular dichroism (CD) are needed to determine enantioenrichment. When synthesized with *R*-BINAP, poly­(**3**) displayed a positive Cotton effect with the maximum CD response observed at 243 nm, indicating the formation of enantioenriched materials (Figure 3A).^51,52^ Cotton effects of opposite directions with identical magnitudes are observed when *S-*BINAP is used during polymerization, demonstrating ligand-controlled enantioenrichment of poly­(**3**) (Figure 3A). A comparison of poly­(**3**) synthesized with the rhodium-*S-*BINAP catalyst to poly­(**3**) synthesized by stereoretentive anionic polymerization from an enantioenriched monomer (*R*-**3**) confirmed that *R*-stereocenters are set in the backbone when using *S*-BINAP. Overall, these data support the formation of isotactic enantioenriched polymers with tunable asymmetry based on catalyst stereochemistry.

**Figure 3:**
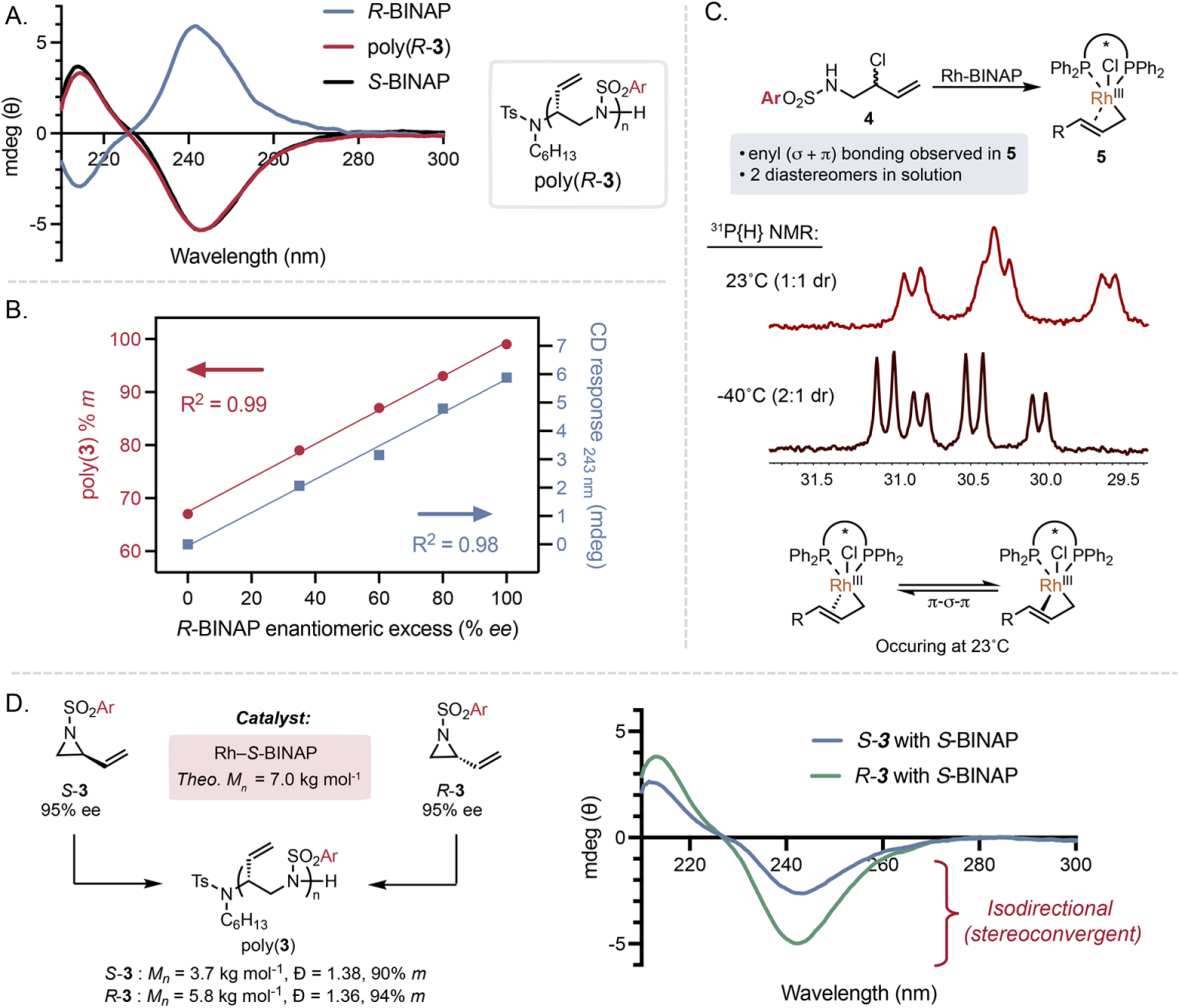
Analysis of enantioselectivity and mechanism supports a stereoconvergent polymerization. (A) CD spectra of poly­(**3**) made using ligands of different enantioenrichment. (B) Poly­(**3**) *meso* content and CD response at 243 nm vs BINAP enantiomeric excess. (C) ^31^P NMR studies of **5**. (D) Polymerization of enantioenriched **3** using *S*-BINAP. ^31^P­{^1^H}, ^1^H-decoupled ^31^P NMR; dr, diastereomeric ratio; Ar = (*rac*)-1-(((2-ethylhexyl)­oxy)­methyl)-4-phenyl.

We sought to understand the relationship among ligand enantiomeric excess (% *ee*), polymer enantioenrichment, and *meso* content in poly­(**3**). To probe this, polymerizations of **3** with a target *M*_*n*_ of 7.0 kg mol^–1^ with varied % *ee* of BINAP were conducted, and the poly­(**3**)­s were accessed with *M*_*n*_ = 6.1 kg mol^–1^, *Đ* = 1.30, and 64–99% *m* (Figures S55 and S57). The CD response at 243 nm of poly­(**3**)­s strongly correlated with the % *ee* of BINAP, supporting an increase in polymer asymmetry from a monomeric rhodium catalyst performing stereoselective propagations to impart enantioselectivity (*R* ^2^ = 0.98, Figure 3B, blue, and Figure S56).^53^ Varying the % *ee* of BINAP likely affects the enantioselectivity of the initiation with **2**, so we performed polymerizations using chiral sulfonamide initiators with enantiopure *R*-BINAP to decouple the role of initiation enantioselectivity on poly­(**3**) asymmetry. Polymerizations with a target *M*_*n*_ of 14.0 kg mol^–1^ using chiral initiators produced poly­(**3**) with *M*_*n*_ = 12.0 kg mol^–1^ and *Đ* = 1.42, and the initiator was observed by ^1^H NMR (Figures S58 and S59). The maximum CD response at 243 nm of poly­(**3**)­s varied based on initiator stereochemistry by up to 20% (1.5 mdeg), indicative of a mismatch effect (Figure S60). However, no significant changes to tacticity were observed by ^13^C NMR, supporting that the enantioselectivity of the initiation has a distinct impact on the asymmetry of the resultant poly­(**3**) compared to subsequent propagations (Figures S61 and S62).^54^ With regard to tacticity, *meso* content strongly correlated with the % *ee* of BINAP as well (Figure 3B, red), suggesting that high levels of *meso* content arise from individual enantioselective propagation events with little effect from the previously enchained monomer, and the chain-end is likely not associated with the catalyst. This is further exemplified with a minimal increase in % *m* when *rac*-BINAP is used compared to ligand-free polymerizations (64% *m* vs 54% *m*, respectively), which we would expect to still have high *meso* content if a coordination–insertion mechanism were operative. All together, these data support high *meso* content arising from high enantioselectivity that is ultimately controlled by a monomeric rhodium catalyst performing monomer activation and biasing enantiofacial addition of the chain-end to the prochiral π-allyl complex.

The operative rhodium-BINAP π-allyl complex was studied to understand the mechanism of stereoconvergence. Direct study of the intermediate derived from **3** and Rh-BINAP proved unsuccessful, so model compound **4** was used (Figures S48 and S49). A stoichiometric reaction between *rac*-**4** and the rhodium-*R-*BINAP catalyst quantitatively provided **5** as a stable 1:1 mixture of diastereotopic η^3^-syn-complexes, supported by ^1^H, ^31^P NMR, and 2D NMR experiments (Figure 3C and Figures S73–S78).^55^ Based on ^1^H and ^13^C NMR data, the η^3^ binding present in **5** displays enyl character, providing a rationale for the observed regioselectivity as oxidative addition and propagation occur via regiospecific S_N_2′ mechanisms (Figure S52).^49^ At 23 °C, slight broadening of ^31^P resonances is observed in **5**, indicating a dynamic process in solution, π–σ–π isomerization, fitting with similar behavior for previous NMR studies of π-allyl intermediates.^56,57^ Evidence for interconversion between diastereomers was observed using variable temperature NMR. Reducing the temperature from 23 to −30 °C resulted in a 2:1 distribution of diastereomers, which recovered to 1:1 when warmed up to 23 °C and was repeatable (Figure 3C, Figures S53 and S54). Diastereomers of **5** are analogous to the diastereomers accessed from oxidative addition into aziridine **3**, which demonstrates that π–σ–π interconversion occurs in solution, which is amenable for stereoconvergent polymerization.

We next explored whether π–σ–π isomerization occurs at similar rates to polymerization, which would enable stereoconvergence. This is accomplished via polymerization of enantioenriched monomers with the same enantiomer of ligand and then analyzing the enantioenrichment of the materials.^29^ Polymerization of both enantiomers of **3** (*ee* > 95%) with the rhodium-*S-*BINAP catalyst successfully produced poly­(**3**) regardless of monomer stereochemistry (Figure 3D). Slight mismatch effects are observed with *R*-**3** in both the molar mass and the tacticity (3.9 vs 5.8 kg mol^–1^ and 90 vs 94% *m*, respectively, Figures S63–S65). However, both poly­(**3**)­s exhibited isodirectional CD responses, indicative of a net stereoenrichment to the *R*-stereocenter in the repeat unit. Variation in magnitudes of CD responses likely arises from molar mass effects on the CD response, supported by control experiments (Figure 3D and Figure S43). The polymerization of enantiopure *S*-**3** with *R*-BINAP is the most extreme case for a catalyst to stereoconverge a monomer to an asymmetric polymer, which provides strong support that π–σ–π isomerization occurs faster than polymerization.

We propose the following catalytic cycle that leads to a stereoconvergent chain-growth polymerization. Based on small-molecule reports, oxidative addition and propagation occur via S_N_2′ mechanisms, leading to net retention of stereochemistry from monomer to polymer in the absence of π–σ–π isomerization.^49^ When using *rac*-**3**, oxidative addition provides a 1:1 mixture of **Pro-** *R* and **Pro-** *S* diastereomers, which we hypothesize remains constant throughout polymerization, supported by studies of **5** at 23 °C. Control of tacticity and enantioselectivity occurs via the selective addition of the chain-end or initiator to one complex and is dictated by the stereochemistry of BINAP (**Pro-** *R* with *S*-BINAP, Figure 4). When π–σ–π isomerization is faster than polymerization, the mismatched monomer’s stereochemistry (*S*-**3** with *S*-BINAP, Figure 4, blue cycle) can invert to the favored diastereomer via a short-lived η^1^ rhodium-allyl intermediate prior to propagation. Altogether, this allows both *R* **-3** and *S* **-3** to be incorporated into poly­(**3**) with identical absolute stereochemistry via a ligand-controlled process.

**Figure 4:**
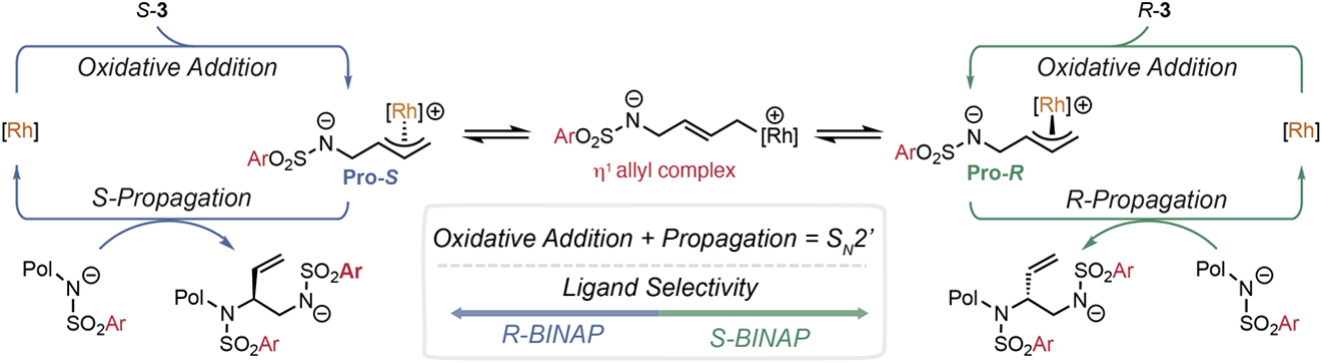
Mechanistic studies support that π–σ–π isomerization occurs faster than propagation. Proposed catalytic cycle for stereoconvergent polymerization via π–σ–π isomerization.

We identified several stereochemistry-induced property changes through postpolymerization modification. The alkenes of poly­(**3**) were reactive to transformations including hydrosilylation, thiol–ene click, and reduction reactions, which produced poly­(**6**), poly­(**7**), and poly­(**8**); CD measurements support that all polymers displayed asymmetric conformations in solution (Figure 5A and Figures S44–S47). Tacticity-induced property changes are observed with poly­(**8**), wherein the isotactic poly­(**8**) (99% *m* by ^13^C NMR, see Figure S24) exhibits semicrystallinity with a *T*_*m*_ of 250 °C as seen with differential scanning calorimetry (DSC) compared to atactic poly­(**8**), where no *T*_*m*_ is observed, though both *T_g_* s remain at 90 °C (Figure 5B). Additionally, RuO_4_-catalyzed C–H oxidation of the benzyl ether moiety of poly­(**8)** produced enantioenriched poly­(**9**). Access to enantioenriched poly­(**9**) synthesized with *R*- and *S*-BINAP enabled identification of a stereocomplex when performing DSC of a 1:1 blend of polymer enantiomers.^11^ We observed a 40 °C increase in the *T*_*m*_ to 242 °C, a sharpening of the exotherm, and an increase in the enthalpy of melting (Δ*H*) to 10.5 J g^–1^ from 1.5 J g^–1^ (Figure 5D). All together, these works justify the development of methods to control both the relative and absolute configurations of a polymer.

**Figure 5:**
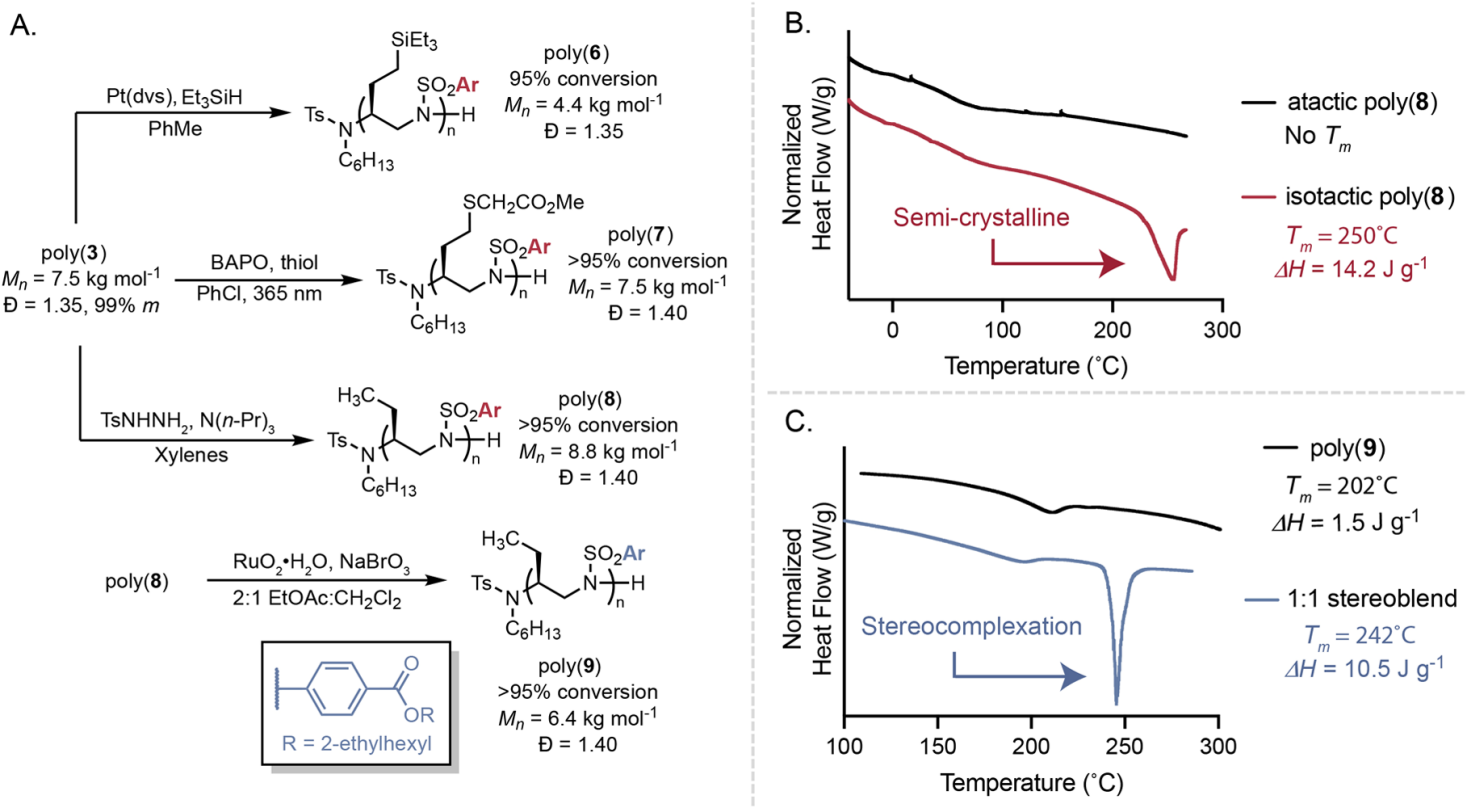
Stereochemistry is responsible for property outcomes. (A) Postpolymerization modification of poly­(**3**). (B) DSC data (second heating cycle, 10 °C/min) of atactic and isotactic poly­(**8**). (C) DSC data (second heating cycle, 10 °C/min) of homochiral and a 1:1 stereoblend of poly­(**9**). *T*_*m*_, melting point; Pt­(dvs), platinum(0)-1,3-divinyl-1,1,3,3-tetra­methyl­disiloxane; BAPO, phenyl­bis­(2,4,6-trimethyl­benzoyl)­phos­phine oxide; thiol, methylthioglycolate.

We have demonstrated the feasibility of decoupling monomer and polymer stereochemistry with a DyKAT mechanism to access an enantioenriched, isotactic polymer by using a single catalyst. Rhodium catalysis proved enabling for the polymerization of vinyl aziridines via π-allyl intermediates. While rhodium is a precious metal, the use of BINAP as an inexpensive and readily available ligand is a benefit; future work will explore more abundant transition metals such as molybdenum^58^ and cobalt^59^ that have precedent in π-allyl chemistry. Further, extending stereoconvergent polymerization via π-allyl intermediates to activated cyclopropanes^60^ or heterocycles containing oxygen^45,61^ and sulfur^62^ will expand the utility of this approach. Overall, this report represents the first example of stereoconvergent chain-growth polymerization to high molar mass polymers and, therefore, serves as a coherent conceptual framework to expand the concept to additional mechanisms and substrates with the opportunity to transform racemic building blocks into enantioenriched, stereodefined polymers.

## Supplementary Material



## Notes

Materials and methods, experimental procedures for polymers and small molecules, characterization data including ^1^H and ^13^C NMR, SEC, TGA, DSC, CD, and MALDI-TOF mass spectrometry data (PDF)
