# Supplementary material for: Stereoconvergent Chain-Growth Polymerization
Source: ACS Cent Sci. 2025 May 5;11(5):797–804. doi: 10.1021/acscentsci.5c00239 (PMC12123547; doi:10.1021/acscentsci.5c00239)
Supplement: Supplementary file 1 [file oc5c00239_si_001.pdf]

Supporting Information for:

Stereoconvergent Chain-growth Polymerization

Jake R. Jagannathan and Frank A. Leibfarth*

Department of Chemistry, University of North Carolina Chapel Hill, Chapel Hill, NC 27599 (USA) *Correspondence to: frankl@email.unc.edu

Table of Contents

1. Materials, Methods and General Guidelines 1
3. Synthesis of Monomers and Initiators 3
3. Optimization 11
4. Polymer Synthesis and Post-polymerization Modification 16
5. MALDI-TOF MS of poly(3) 40
6. Characterization of tacticity and enantioselectivity of polymers 41
7. Circular Dichroism and Optical Rotation of polymers 45
8. NMR Studies of model π-allyl complex 49
9. Mechanistic Analysis 55
10. References 63
11. Characterization Data 65

1. Materials, Methods and General Guidelines

1.1 General considerations

Unless otherwise noted, solvents were dried and degassed using a Pure Process Technology solvent purification system and then subsequently stored over molecular sieves (3Å) in a N2-filled glovebox. Other reagents whose syntheses are not described were purchased from commercial sources (Alfa Aesar (Ward Hill, MA), Sigma-Aldrich (St. Louis, MO), Oakwood Products (West Columbia, SC), Acros Organics (Geel, Belgium), Strem, and TCI America (Portland, OR)) and used without further purification. All syntheses were performed under an inert N_2 atmosphere using flame-dried or oven-dried glassware or in a N_2-filled glovebox unless specified otherwise. Thin layer chromatography (TLC) was performed on SiliaPlate 250µm thick silica gel plates provided by Silicycle. Visualization was accomplished with short wave UV light (254 nm), aqueous basic potassium permanganate solution, or aqueous acidic ceric ammonium molybdate solution followed by heating. Flash chromatography was performed using SiliaFlash P60 silica gel (40-63 µm) purchased from Silicycle.

1.2 Characterization

NMR NMR spectra were recorded using a Bruker Neo 400 MHz, or Bruker AVANCE III 600 MHz CryoProbe spectrometer. Chemical shifts δ (ppm) are referenced to tetramethylsilane(TMS) using the residual solvent as an internal standard (^{1}H and ^{13}C). For ^{1}H NMR: $CDCl_3$ = 7.26 ppm, CD_2Cl_2 = 5.30. For ^{13}C NMR: $CDCl_3$ = 77.16 ppm, CD_2Cl_2 = 53.52. Coupling constants (J) are expressed in hertz (Hz). ^{1}H NMR data are reported as follows: chemical shift, multiplicity (s = singlet, d = doublet, t = triplet, q = quartet, m = multiplet, dd = doublet of doublets, dt = doublet of triplets, bs = broad singlet), coupling constants (Hz), and integration. For tacticity calculations, a 13C NMR spectrum with 8192 scans with a 1-second relaxation delay was collected with concentrated polymer samples (50 mg/mL). Splines baseline correction was applied prior to analysis.

THF GPC Tosoh EcoSEC Elite GPC
Gel permeation chromatography (GPC) was performed on a Tosoh EcoSEC Elite GPC system equipped with a TSKgel Super HM-M (17392) column maintained at 40 °C with an RI detector and a Tosoh LENSTM 3 multi-angle light scattering detector (MALS) or an Agilent Technologies 1260 Infinity II instrument equipped with two PL gel 10 µm mixed-B LS columns connected in series, with tetrahydrofuran (THF) (35 °C) as the eluent,. Tetrahydrofuran was used as the mobile phase at a flow rate of 0.5 mL/min. Molecular weight and dispersity data are reported relative to polystyrene standards for both instruments.

Chloroform GPC Agilent 1260 Infinity
Gel permeation chromatography (GPC) for samples were performed on an Agilent 1260 Infinity separation module liquid chromatograph equipped with two Agilent Resipore Columns (PL1113- 6300) maintained at 50 °C, and an Agilent 1260 RID G1362A refractive index detector at 50 °C. Chloroform ($CHCl_3$, containing amylene as a stabilizer) was used as the mobile phase at a flow rate of 1.0 mL/min. Molecular weight and dispersity data are reported relative to polystyrene standards.

TGA TA Instruments Q5000 Thermogravimetric Analyzer
Decomposition onset temperatures (T_d) of precipitated and dried polymer samples were measured by thermal gravimetric analysis (TGA) on a TA Instruments Q5000 Thermogravimetric Analyzer. Polymer samples were heated from ambient temperatures to 600 °C at a heating rate of 10 °C/min. Values of T_d (temperature at 5% weight loss) were obtained from wt % vs. temperature (°C) plots.

DSC TA Instruments Discovery DSC
Melting-transition temperature (T_m) and glass-transition temperature (T_g) of precipitated and dried polymer samples were measured using differential scanning calorimetry (DSC) on a TA Instruments Discovery DSC. Unless specifically noted otherwise, values for T_m and T_g were obtained from a second heating scan after the thermal history was removed. Scan rates are included with individual entries.

HRMS High-resolution mass spectrometry (HRMS) was performed with a ThermoScientific Q Exactive HF-X mass spectrometer using electrospray ionization at the University of North Carolina Mass Spectrometry Core Laboratory.

MALDI-TOF MS Analysis of polymers was conducted using a Bruker ultraflextreme MALDI-TOF mass spectrometer (Bruker Daltonics, Billerica, MA). Samples were prepared by making solutions of *trans*-2-[3-(4-*tert*-Butylphenyl)-2-methyl-2-propenylidene]malononitrile (DCTB, 20 mg/mL), polymer (5 mg/mL), NaTFA (1 mg/mL). A 26 μL matrix was made containing 20 μL of DCTB solution, 5 μL analyte solution, and 1 μL NaTFA solution was combined and mixed thoroughly. 1 μL of the matrix solution was applied using the droplet method. Samples were analyzed in reflector mode.

IR Infrared (IR) spectra were obtained using a PerkinElmer Frontier FT-IR spectrometer.

CD Circular dichroism (CD) measurements were collected on a Chirascan Plus instrument with a wavelength range of 250-350 nm in 1.0 nm steps with 4.000 sec scans per point. Samples were observed in a 1 mm quartz cuvette at 0.1 mg/mL in HPLC grade, filtered THF. The sample temperature was controlled using a Quantum Northwester TC125 unit with Julabo AWC100 water bath. A Savitzky-Golay smoothing filter with window size < 8 was applied to the chromatogram.

Optical Rotation Optical rotations were collected using a Jasco P-1010 polarimeter at 25 ºC, using a 2 mL cell with 1 dm path length. Data was collected in HPLC-grade chloroform at 0.25 mg/mL

HPLC High pressure liquid chromatography was performed using an Agilent Series 1260 HPLC system equipped with a 1260 diode array detector. Daicel Chiralpak IC (two columns, first received: August 2014, second received November 2019), were available for use in separations.

3. Synthesis of Monomers and Initiators

2.1 Tosyl initiator 1

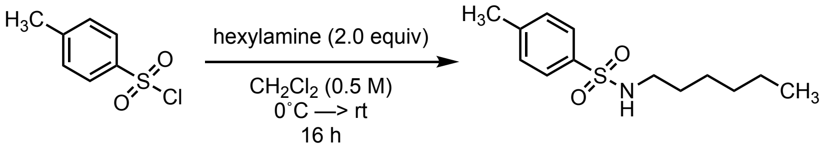

Following a reported procedure,[1] a flame-dried round-bottom flask with an egg-shaped stir-bar was charged with tosyl chloride (1.91 g, 10.0 mmol, 1.0 equiv), purged with N_2 for 15 min, followed by the addition of CH_2Cl_2 (20 mL, 0.5 M). The reaction flask was cooled to 0˚C using an ice bath, and hexylamine (2.64 mL, 20.0 mmol, 2.0 equiv) was added dropwise. After the addition, the reaction was allowed to warm to room temperature and stirred for 16 h. The reaction was filtered using a fritted Büchner funnel to remove precipitates, and the precipitate was washed with CH_2Cl_2 (3 x 10 mL). The organic solution was washed with brine (20 mL), dried with $MgSO_4$, and the solvent was removed under reduced pressure. The crude product was loaded onto silica gel and purified by column chromatography (90:10 to 40:60 hexanes:ethyl acetate) to furnish the product **1** as a white solid in 78% yield (1.99 g, 7.83mmol). Spectral assignments are in agreement with previous reports.[1]

R_f (2:1 hexanes/ethyl acetate) = 0.6 (UV and $KMnO_4$ stain)

^{1}H NMR (400 MHz, $CDCl_3$) δ 7.78 – 7.71 (m, 1H), 7.31 (d, J = 8.0 Hz, 1H), 4.28 (t, J = 6.3 Hz, 0H), 2.93 (q, J = 6.8 Hz, 1H), 2.43 (s, 1H), 1.44 (p, J = 7.1 Hz, 1H), 1.31 – 1.07 (m, 2H), 0.85 (t, J = 6.9 Hz, 1H).

^{13}C NMR (100 MHz, $CDCl_3$) 143.5, 137.1, 129.8, 127.3, 43.4, 31.4, 29.7, 26.3, 22.6, 21.7, 14.1.

2.2 Tosyl monomer 2

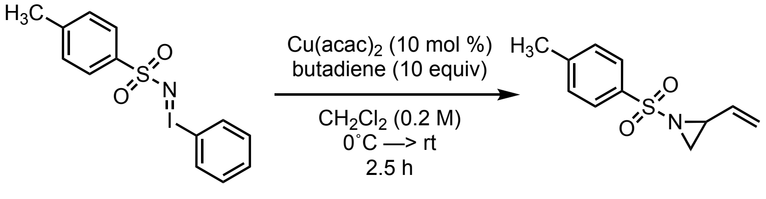

Following a reported procedure,[2] a flame-dried three-neck flask with an egg-shaped stir-bar was charged with phenyl-λ3-iodaneylidene arylsulfonamide (8.60 g, 23.0 mmol). The flask was purged with Ar for 10 min, followed by the addition of dichloromethane (0.2 M), and butadiene (42 mL of a 2.77 M solution in toluene, 10 equiv). The flask was cooled to 0˚C in an ice bath and $Cu(acac)_2$ (10 mol %, 0.603 g, 2.30 mmol) was added in a single portion. The flask stirred at 0˚C for 15 min, warmed to room temperature, and stirred for 2.5 h, or until the solution became homogeneous. The reaction contents were filtered through a pad of silica gel using a fritted Büchner funnel to remove copper salts, and the solvent was removed under reduced pressure, loaded onto celite, and purified by column chromatography (100:0 to 80:20 hexanes/ethyl

acetate) furnished the product as (white solid) in 70% yield (3.60 g, 16.1 mmol). Spectral assignments are in agreement with previous reports.[2]

Note the product was placed in a -20°C freezer for 16 h and dried under vacuum (<1 torr) to promote crystallization to a solid after column chromatography

R_f (9:1 hexanes/ethyl acetate) = 0.22 (UV and $KMnO_4$ stain)

^{1}H NMR (600 MHz, $CDCl_3$) δ 7.83 (d, *J* = 8.3 Hz, 2H), 7.34 (d, *J* = 7.9 Hz, 2H), 5.51 (ddd, *J* = 17.3, 10.1, 7.4 Hz, 1H), 5.43 (dd, *J* = 17.2, 1.3 Hz, 1H), 5.24 (dd, *J* = 10.2, 1.1 Hz, 1H), 3.27 (td, *J* = 7.2, 4.5 Hz, 1H), 2.78 (d, *J* = 7.1 Hz, 1H), 2.44 (s, 3H), 2.22 (d, *J* = 4.5 Hz, 1H).

^{13}C NMR (151 MHz, $CDCl_3$) δ 144.7, 134.9, 133.1, 129.9, 128.0, 120.1, 41.1, 34.3, 21.8.

2.3 Ethylhexyl monomer 3

Synthesis of 4-(bromomethyl)benzenesulfonamide

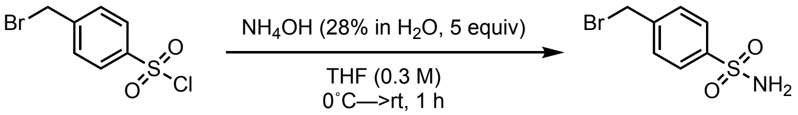

NH_4OH (28 % in H_2O) (9.30 mL, 138.3 mmol, 5 equiv) was added to a stirred solution of 4-bromomethylbenzenesulfonyl chloride (10.0 g, 37.1 mmol) in THF (120 mL) at 0 °C. The solution was stirred at room temperature for 1 h. the mixture was diluted with H_2O and the product was extracted with EtOAc (100 mL x 3). Organic layers were dried over anhydrous Na_2SO_4 and concentrated in vacuo to give 4-(bromomethyl)benzenesulfonamide as a white powder (9.10 g, 9.8 mmol, 98 % yield). The material was used in the next step without purification. Spectral data match with previously reported values.[3]

^{1}H-NMR (400 MHz, DMSO-*d*$_6$) δ; 7.78 (2H, d, J=8.5Hz), 7.63 (2H, d, J=8.5Hz), 7.40 (2H, s), 4.75 (2H, s).

^{13}C-NMR (101 MHz, DMSO-*d*$_6$) δ: 143.8, 141.9, 129.8, 126.0, 32.9.

R_f= 0.40 (CH_2Cl_2/EtOH 19:1)

Synthesis of *rac*-4-(((2-ethylhexyl)oxy)methyl)benzenesulfonamide

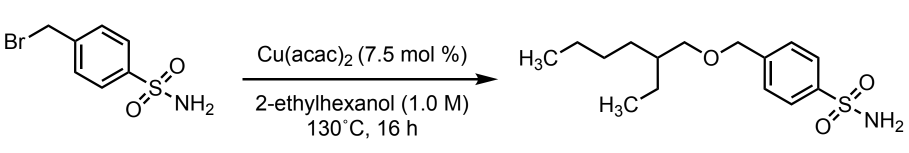

With slight modifications to a reported procedure,[4] a 50 mL round bottom flask with a stirbar was charged with copper(II) acetylacetenoate (560 mg, 2.14 mmol, 7.5 mol %), 4-(bromomethyl)benzenesulfonamide (7.15 g, 28.6 mmol), followed by 2-ethylhexanol (27.0 mL, 172 mmol, 6 equiv). The suspension was

allowed to stir at room temperature for 15 min. The flask was placed in an oil bath heated to 130˚C and stirred overnight. The reaction was allowed to cool to room temperature, and the flask was connected to a short-path distillation column, and 2-ethylhexanol was distilled off from the reaction mixture (60-90˚C, <1 torr), which took approximately three hours. A sample was analyzed using ^{1}H MR to confirm the absence of 2-ethylhexanol from the crude mixture. The distillation was allowed to cool to room temperature, and the remaining material was dissolved in 5% MeOH in CH_2Cl_2 (20 mL), and passed through a silica gel plug (200 g) using 5% MeOH in CH_2Cl_2 (500 mL). The solvent was removed under reduced pressure to furnish 4-(((2-ethylhexyl)oxy)methyl)benzenesulfonamide as a brown oil (7.00 g, 28.6 mmol, 82% yield).

^{1}H NMR (400 MHz, $CDCl_3$) δ 7.93 (d, J = 8.4 Hz, 2H), 7.51 (d, J = 8.1 Hz, 2H), 4.93 (bs, 2H), 4.58 (s, 2H), 3.40 (d, J = 5.9 Hz, 2H), 1.58 (h, J = 6.0 Hz, 1H), 1.52 – 1.22 (m, 8H), 0.91 (m, 6H).

^{13}C NMR (151 MHz, $CDCl_3$) δ 144.46, 140.81, 127.66, 126.50, 73.79, 72.04, 39.77, 30.58, 29.11, 23.90, 23.08, 14.12, 11.12.

IR: 3265 (s), 2957 (s), 2928 (s), 2858 (s), 1329 (m), 1158 (s), 1092 (m), 735 (w), 540 (m)

ESI: Expected [M+Na] = 322.1453; Found: 322.1456

Synthesis of *rac*-1-((4-(((2-ethylhexyl)oxy)methyl)phenyl)sulfonyl)-2-vinylaziridine

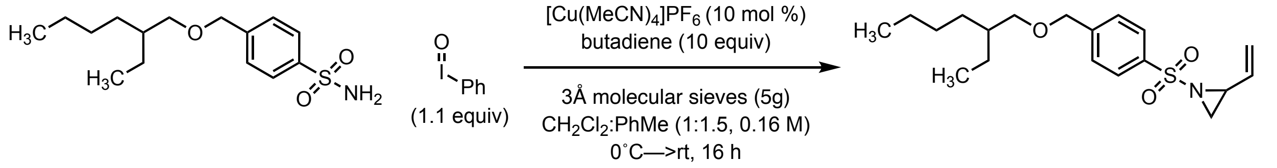

Based on a reported procedure,[5] a 2-neck round bottom flask with a stirbar and 3Å molecular sieves (5.00 g, dried for 48 h at 160˚C) was flame dried under vacuum, back-filled with nitrogen, allowed to cool to room temperature, and transferred to a nitrogen-filled glovebox. In the glovebox, tetrakis(acetonitrile)copper(I) hexafluorophosphate (0.436 g, 1.17 mmol, 10 mol %) was added to the flask, and the flask was sealed with rubber septa and taken out of the glovebox. To the flask, a solution of butadiene (42 mL of a 2.77 M solution in PhMe) was added, followed by a solution of *rac*-4-(((2-ethylhexyl)oxy)methyl)benzenesulfonamide (3.50 g, 11.7 mmol) in CH_2Cl_2 (29 mL) was added (Note: the order of addition was found to affect yield and highest yields were obtained with addition of butadiene followed by the sulfonamide). The flask was cooled to 0˚C, and iodosyl benzene (2.83 g, 12.9 mmol, 1.1 equiv) was added in portions over a 2.5 h period ((0.57 g each time, five portions in total). Once the addition was complete, the reaction stirred overnight and was allowed to warm to room temperature. The reaction contents were filtered through a pad of silica gel to remove copper salts, washed with CH_2Cl_2 (50 mL) and the solvent was removed under reduced pressure. The crude contents were loaded onto celite and purified by column chromatography with silica gel (98:2 to 80:20 hexanes/ethyl acetate) to furnish *rac*-1-((4-(((2-ethylhexyl)oxy)methyl)phenyl)sulfonyl)-2-vinylaziridine as a yellow oil (2.61g, 7.43 mmol, 64% yield).

No evidence of diastereoenrichment was observed by NMR

R_f (9:1 hexanes/ethyl acetate) = 0.28 (UV and $KMnO_4$ stain)

^{1}H NMR (600 MHz, CDCl$_3$) δ 7.94 (d, J = 8.4 Hz, 2H), 7.53 (d, J = 8.5 Hz, 2H), 5.54 (ddd, J = 17.3, 10.1, 7.4 Hz, 1H), 5.45 (dd, J = 17.2, 1.5 Hz, 1H), 5.27 (dd, J = 10.4, 1.2 Hz, 1H), 4.59 (s, 2H), 3.41 (dd, J = 5.8, 1.8 Hz, 2H), 3.32 (td, J = 7.2, 4.5 Hz, 1H), 2.82 (d, J = 7.1 Hz, 1H), 2.25 (d, J = 4.5 Hz, 1H), 1.58 (h, J = 6.1 Hz, 1H), 1.50 – 1.24 (m, 9H), 0.91 (m, 6H).

^{13}C NMR (101 MHz, CDCl$_3$) δ 145.5, 136.9, 133.0, 128.0, 127.65 120.5, 73.9, 72.1, 41.2, 39.9, 34.3, 30.7, 29.2, 24.0, 23.2, 14.2, 11.2.

IR: 2957 (s), 2928 (s), 2858 (s), 1736 (s), 1326 (s), 1159 (m), 929 (m), 629 (w)

ESI: Expected [M+H] = 352.1491; Found: 352.1491

Synthesis of *N*-(2-chlorobut-3-en-1-yl)-4-(((2-ethylhexyl)oxy)methyl)benzenesulfonamide (4)

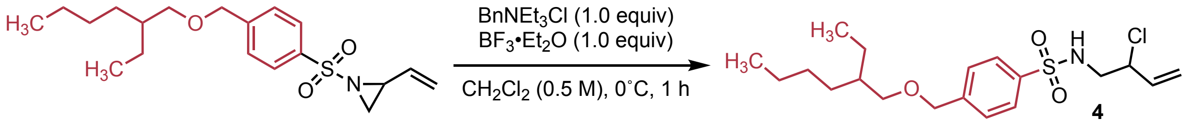

4

Based on a reported procedure,[6] to a stirred solution of *rac*-1-((4-(((2-ethylhexyl)oxy)methyl)phenyl)sulfonyl)-2-vinylaziridine (0.350 g, 1.00 mmol, 1.0 equiv) and benzyltriethylmmonium chloride (0.141g, 1.00 mmol, 1.0 equiv) in dry CH_2Cl_2 (2 mL, 0.5 M) was added $BF_3·OEt_2$ (0.126 mL, 1.00 mmol, 1.0 equiv) dropwise at 0 °C and the reaction stirred for 1 h. After completion of the reaction (monitored by TLC), it was quenched with water. The product was extracted by CH_2Cl_2 (3 × 2.0 mL) and dried over anhydrous Na_2SO_4. After removal of the solvent the crude product was purified by flash column chromatography on silica gel (98:2 to 70:30 hexanes/ethyl acetate) to furnish *N*-(2-chlorobut-3-en-1-yl)-4-(((2-ethylhexyl)oxy)methyl)benzenesulfonamide as an oil (0.350g, 0.91 mmol, 91% yield).

R_f (9:1 hexanes/ethyl acetate) = 0.15 (UV and $KMnO_4$ stain)

^{1}H NMR (600 MHz, CDCl$_3$) δ 7.84 (d, J = 8.4 Hz, 2H), 7.50 (d, J = 8.6 Hz, 2H), 5.77 (ddd, J = 16.9, 10.2, 7.9 Hz, 1H), 5.33 (d, J = 16.9 Hz, 1H), 5.25 (d, J = 10.2 Hz, 1H), 4.90 – 4.80 (m, 1H), 4.56 (s, 2H), 4.37 (td, J = 7.7, 5.2 Hz, 1H), 3.39 (dd, J = 5.9, 2.0 Hz, 2H), 3.33 (ddd, J = 12.8, 7.4, 5.2 Hz, 1H), 3.20 (ddd, J = 13.5, 7.6, 5.7 Hz, 1H), 1.57 (hept, J = 6.1 Hz, 1H), 1.48 – 1.20 (m, 8H), 0.89 (m, 6H).

^{13}C NMR (151 MHz, CDCl$_3$) δ 144.7, 138.7, 134.6, 127.8, 127.1, 119.7, 73.8, 72.0, 60.9, 48.8, 39.8, 30.6, 29.1, 23.9, 23.1, 14.1, 11.1.

IR: 3280 (s), 2926 (s), 1601 (w), 1457 (m), 1329 (s), 1158 (s), 1091 (s), 683 (m)

ESI: Expected [M+H] = 388.1713, Found : 388.1717

Synthesis of *rac*-1-bromo-4-(((2-ethylhexyl)oxy)methyl)benzene

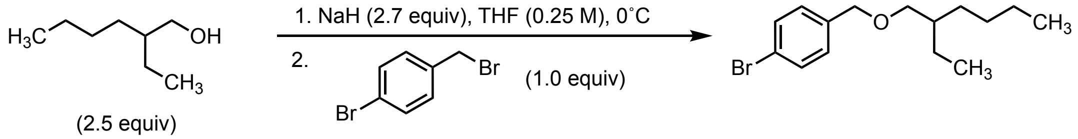

A flame-dried, 2-neck round-bottomed flask with a stir bar was charged with 2-ethylhexanol (11.7 mL, 75.0 mmol, 2.5 equiv), followed by THF (120 mL). The solution was cooled to 0˚C with an ice bath and NaH (60% dispersion in mineral oil, 3.18 g, 81 mmol, 2.5 equiv) was added in portions over 15 minutes and the solution stirred at 0˚C until bubbling ceased. After bubbling ceased, a solution of 4-bromobenzyl bromide (7.50g, 30.0 mmol) in dry THF (20 mL) was added over 5 min, and the solution turned a light yellow color. The temperature was increased to 55˚C and the solution stirred for 16 h. The reaction was then cooled to 0˚C and quenched with sat'd. aq. NH_4Cl (30 mL) and stirred for 10 min. The product was extracted with EtOAc (3x100 mL) and the organic layer was separated and washed with brine (50 mL). The aqueous layer was removed and the organic layer was dried with Na_2SO_4, filtered, and the solvent was removed under reduced pressure. The product was purified via column chromatography in pure hexanes to furnish 1-bromo-4-(((2-ethylhexyl)oxy)methyl)benzene as a clear oil (7.50 g, 25.1 mmol, 83% yield).

^{1}H NMR (600 MHz, $CDCl_3$) δ 7.46 (d, J = 8.4 Hz, 2H), 7.21 (d, J = 8.5 Hz, 2H), 4.44 (s, 2H), 3.34 (dd, J = 5.9, 2.3 Hz, 2H), 1.53 (dp, J = 12.2, 5.7 Hz, 1H), 1.45 – 1.20 (m, 10H), 0.94 – 0.82 (m, 6H).

^{13}C NMR (151 MHz, $CDCl_3$) δ 138.1, 131.5, 129.2, 121.3, 73.4, 72.3, 39.9, 30.7, 29.2, 24.0, 23.2, 14.2, 11.2.

IR: 2956 (m), 2923 (s), 2854 (s), 1468 (s), 1457 (m), 1092 (s), 801 (s)

Did not ionize with ESI-MS

Synthesis of methyl ((4-(((2-ethylhexyl)oxy)methyl)phenyl)sulfonyl)-*D*-serinate

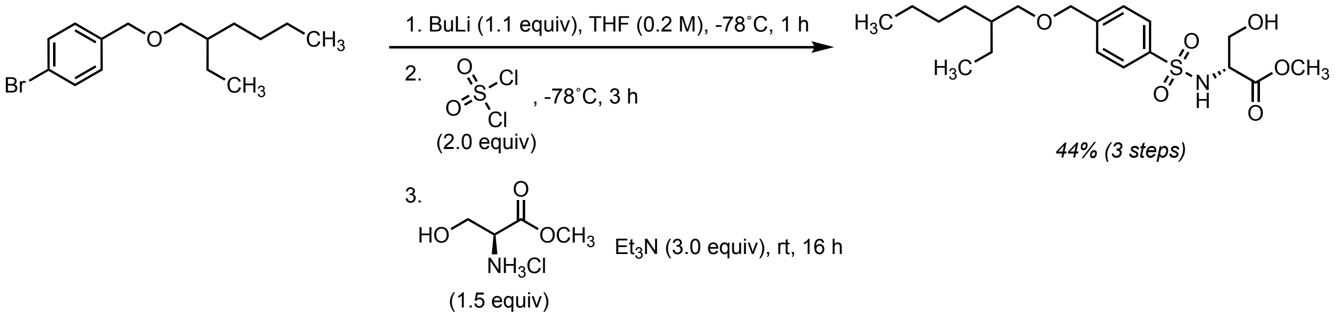

A flame-dried round-bottomed flask with a stir bar was charged with 1-bromo-4-(((2-ethylhexyl)oxy)methyl)benzene (1.19 g, 4.00 mmol), followed by THF (16 mL). The flask was cooled to -78˚C and nBuLi (2.5 M in hexanes, 1.75 mL, 4.4 mmol, 1.1 equiv) was added drop-wise and the reaction stirred at -78˚C for 1 h. Sulfuryl chloride (0.67 mL, 8.4 mmol, 2.1 equiv), was added quickly via syringe and the reaction stirred at -78˚C for 3 h. The reaction was quenched by the addition of brine (15 mL) and

the flask was allowed to warm to room temperature. The reaction was diluted and extracted with EtOAc (3 x 25 mL). The organics were collected, dried with Na_2SO_4, filtered, and the solvent was removed under reduced pressure to produce the crude sulfonyl chloride. The crude reaction was dissolved in THF (10 mL) and added drop-wise to a flame-dried flask with a stir bar containing a solution of *D*-serine methyl ester hydrochloride (0.621 g, 4.00 mmol, 1.0 equiv), triethylamine (1.67 mL, 12.0 mmol, 3.0 equiv) in THF (16 mL). The reaction stirred at room temperature for 16 h. After stirring, the reaction was diluted with water (20 mL) and extracted with EtOAc (3 x 20 mL). The organic contents were washed with 10% citric acid (15 mL x 3), sat'd aq. $NaHCO_3$ (15 mL x 3) and brine (15 mL). The organic layer was separated and dried with Na_2SO_4, filtered and the solvent was removed under reduced pressure. The crude was loaded onto celite and purified via flash chromatography in hexanes/ethyl acetate (80:20→0:100) to furnish methyl ((4-(((2-ethylhexyl)oxy)methyl)phenyl)sulfonyl)-*D*-serinate as a white solid (0.714 g, 1.78 mmol , 44% yield over 2 steps).

The same procedure was followed with L-serine to obtain the enantiomeric product

R_f (1:1 hexanes/ethyl acetate) = 0.50 (UV and $KMnO_4$ stain)

^{1}H NMR (600 MHz, $CDCl_3$) δ 7.86 (d, J = 8.4 Hz, 2H), 7.51 (d, J = 8.5 Hz, 2H), 5.55 (d, J = 7.3 Hz, 1H), 4.57 (s, 2H), 4.00 (dt, J = 7.3, 3.7 Hz, 1H), 3.93 (ddd, J = 6.7, 3.7, 1.4 Hz, 2H), 3.66 (s, 3H), 3.41 (dd, J = 5.9, 1.9 Hz, 2H), 2.11 (t, J = 6.6 Hz, 1H), 1.59 (dt, J = 12.2, 6.0 Hz, 1H), 1.50 – 1.24 (m, 10H), 0.91 (m, 6H).

^{13}C NMR (151 MHz, $CDCl_3$) δ 170.1, 145.0, 138.1, 127.8, 127.4, 74.0, 72.1, 63.9, 57.6, 53.2, 39.9, 30.7, 29.2, 24.0, 23.2, 14.2, 11.2.

IR: 3506 (s), 3273 (s), 2955 (s), 2930 (s), 2857 (s), 1748 (s), 1327 (m), 1089 (s), 578 (m)

ESI: Expected [M+K] = 440.1509, Found : 440.1509

Synthesis of methyl (2*R*)-1-((4-(((2-ethylhexyl)oxy)methyl)phenyl)sulfonyl)aziridine-2-carboxylate

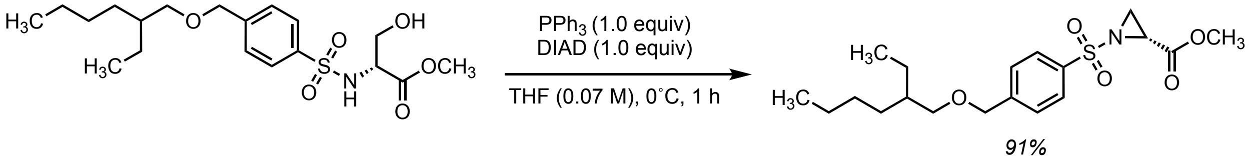

A flame-dried round-bottomed flask with a stir bar was charged with methyl ((4-(((2-ethylhexyl)oxy)methyl)phenyl)sulfonyl)-*D*-serinate (1.27 g, 3.2 mmol), triphenylphosphine (0.830 g, 3.2 mmol) followed by THF (45 mL). The reaction was cooled to 0˚C, and a solution of DIAD (0.62 mL, 3.2 mmol) in THF (10 mL) was added drop-wise at 0˚C and the reaction stirred for 1 h and monitored by TLC. Once complete, the solvent was removed under reduced pressure and the crude mixture was loaded onto celite and purified using flash chromatography in hexanes/ethyl acetate (90:0 to 50:50) to furnish methyl (2*R*)-1-((4-(((2-ethylhexyl)oxy)methyl)phenyl)sulfonyl)aziridine-2-carboxylate as an oil (1.10 g, 2.9 mmol, 91% yield).

The same procedure was followed with L-serine to obtain the enantiomeric product

R_f (4:1 hexanes/ethyl acetate) = 0.30 (UV and $KMnO_4$ stain)

^{1}H NMR (400 MHz, $CDCl_3$) δ 7.96 (d, *J* = 8.5 Hz, 2H), 7.55 (d, *J* = 8.6 Hz, 2H), 4.60 (s, 2H), 3.77 (s, 3H), 3.50 – 3.32 (m, 3H), 2.81 (d, *J* = 7.1 Hz, 1H), 2.60 (d, *J* = 4.0 Hz, 1H), 1.58 (d, *J* = 11.1 Hz, 1H), 1.52 – 1.22 (m, 10H), 0.92 (m, 6H).

^{13}C NMR (101 MHz, $CDCl_3$) δ 167.3, 146.2, 135.8, 128.4, 127.7, 74.0, 72.0, 53.0, 39.9, 35.8, 32.2, 30.72, 29.2, 24.0, 23.2, 14.2, 11.2.

IR: 2956 (m), 2927 (m), 2858 (m), 1747 (s), 1332 (m), 1161 (m), 905 (m)

ESI: Expected [M+K] = 422.1398 Found: 422.1399

Synthesis of (2*S*)-1-((4-(((2-ethylhexyl)oxy)methyl)phenyl)sulfonyl)-2-vinylaziridine

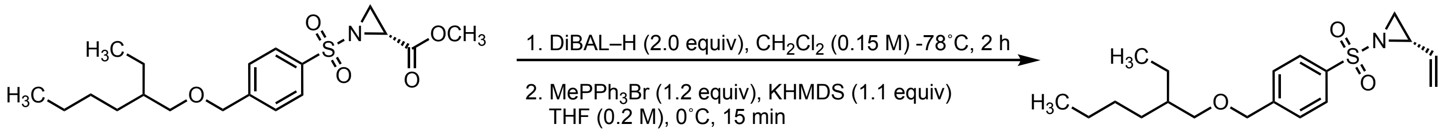

A flame-dried round-bottomed flask with a stir bar was charged with (2*R*)-1-((4-(((2-ethylhexyl)oxy)methyl)phenyl)sulfonyl)aziridine-2-carboxylate (1.00 g, 2.6 mmol) followed by CH_2Cl_2 (18 mL). The reaction was cooled to -78˚C using a dry ice/acetone bath and DiBAL-H (1.0 M in CH_2Cl_2, 3.4 mL, 3.4 mmol, 1.1 equiv) was added drop-wise over 5 min and stirred for 2 h at -78˚C. The reaction was quenched with the addition of aq. HCl (1 M, 10 mL) and the flask warmed up to room temperature. The contents were extracted with CH_2Cl_2 (25 mL x 3) and the organic portion was washed with brine, dried with Na_2SO_4, and the solvent was removed under reduced pressure and placed on a Schlenk line under high vacuum (<1 torr) for 1 h. A sample was analyzed by ^{1}H NMR spectroscopy to confirm the presence of aldehyde. The product was carried to the next step without purification.

In a separate flame-dried round-bottomed flask with a stir bar was charged with methyltriphenylphosphonium bromide (1.21 g, 3.4 mmol, 1.2 equiv) followed by THF (14 mL). The solution was cooled to 0˚C and KHMDS (0.7 M in PhMe, 4.5 mL, 3.1 mmol, 1.1 equiv) was added drop-wise and the solution turned yellow and stirred for 15 min at 0˚C. A solution of aldehyde (*vide infra*) in THF (5 mL) was added quickly at 0˚C and the reaction stirred for 15 min and was quenched with saturated aq. NH_4Cl (15 mL). The reaction was extracted with EtOAc (15 mL x 3) and the organics were collected and washed with brine (15 mL). The organics were separated and dried with Na_2SO_4, filtered and the solvent was removed under reduced pressure. The crude reaction was loaded onto celite and purified by flash chromatography in hexanes/ethylacetate (100:0→80:20) to furnish (2*S*)-1-((4-(((2-ethylhexyl)oxy)methyl)phenyl) sulfonyl)-2-vinylaziridine as an oil (0.121 g, 0.34 mmol, 12% yield).

The same procedure was followed with L-serine to obtain the enantiomeric product

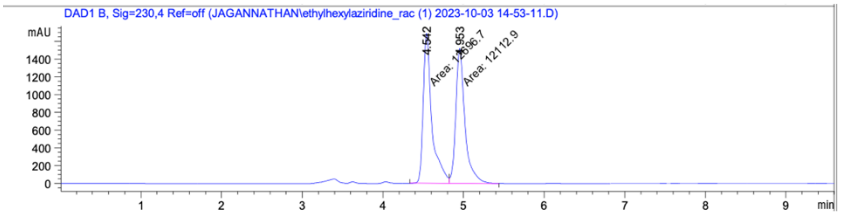

Signal 2: DAD1 B, Sig=230,4 Ref=off

Peak #	RetTime [min]	Type	Width [min]	Area [mAU*s]	Height [mAU]	Area %
1	4.542	MF	0.1251	1.26967e4	1691.34119	51.1766
2	4.953	FM	0.1329	1.21129e4	1518.98743	48.8234
Totals :				2.48096e4	3210.32861	

L-AA derived

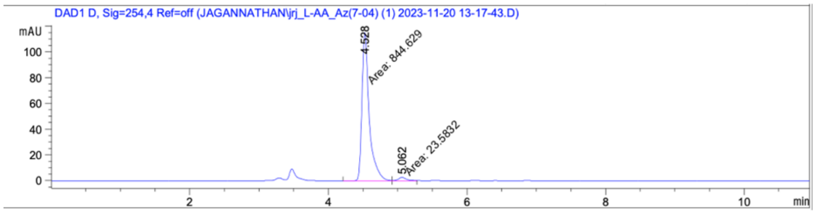

Signal 4: DAD1 D, Sig=254,4 Ref=off

Peak #	RetTime [min]	Type	Width [min]	Area [mAU*s]	Height [mAU]	Area %
1	4.528	MF	0.1220	844.62866	115.39095	97.2837
2	5.062	FM	0.1459	23.58316	2.69460	2.7163
Totals :				868.21182	118.08554	

D-AA derived

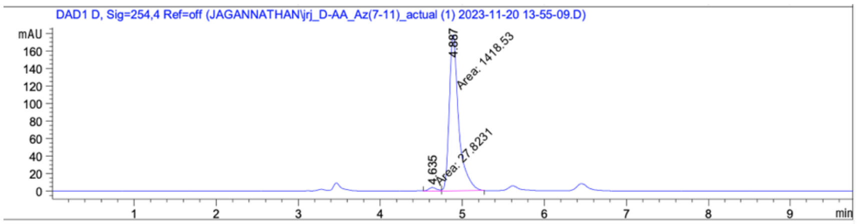

Signal 4: DAD1 D, Sig=254,4 Ref=off

Peak #	RetTime [min]	Type	Width [min]	Area [mAU*s]	Height [mAU]	Area %
1	4.635	MF	0.1145	27.82307	4.05075	1.9237
2	4.887	FM	0.1329	1418.52551	177.90469	98.0763
Totals :				1446.34858	181.95544	

Figure S1. Chiral stationary phase HPLC of **3** using an AD-H column and 90:10 hexanes:2-propanol eluent.

3. Optimization

3.6 Pre-catalyst Screen

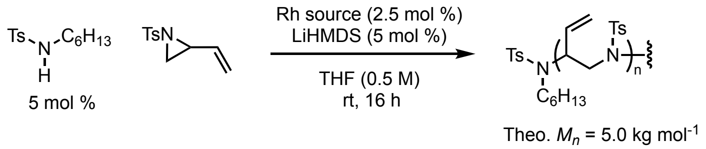

Table S1. Pre-catalyst screen

Pre-catalyst	Conversion (NMR)	M_n ($CHCl_3$, RI) (kg•mol^{-1})	Đ
$[Rh(C_2H_4)Cl]_2$	>95	2.1	1.30
$[Rh(COD)BF_4]_2$	50%	0.4	ND
$[Rh(COD)OTf]_2$	>95	0.3	ND
$[Rh(COD)OH]_2$	>95	0.6	ND
$[Rh(COD)SbF_6]_2$	65	0.5	ND
$[Rh(COD)BAr^F{}_4]_2$	30	0.8	ND

3.6 Additional Ligands Explored

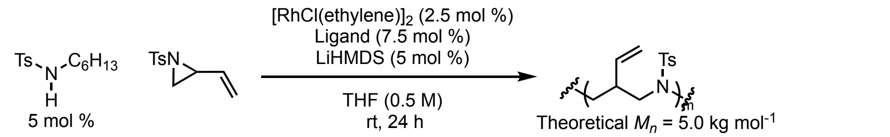

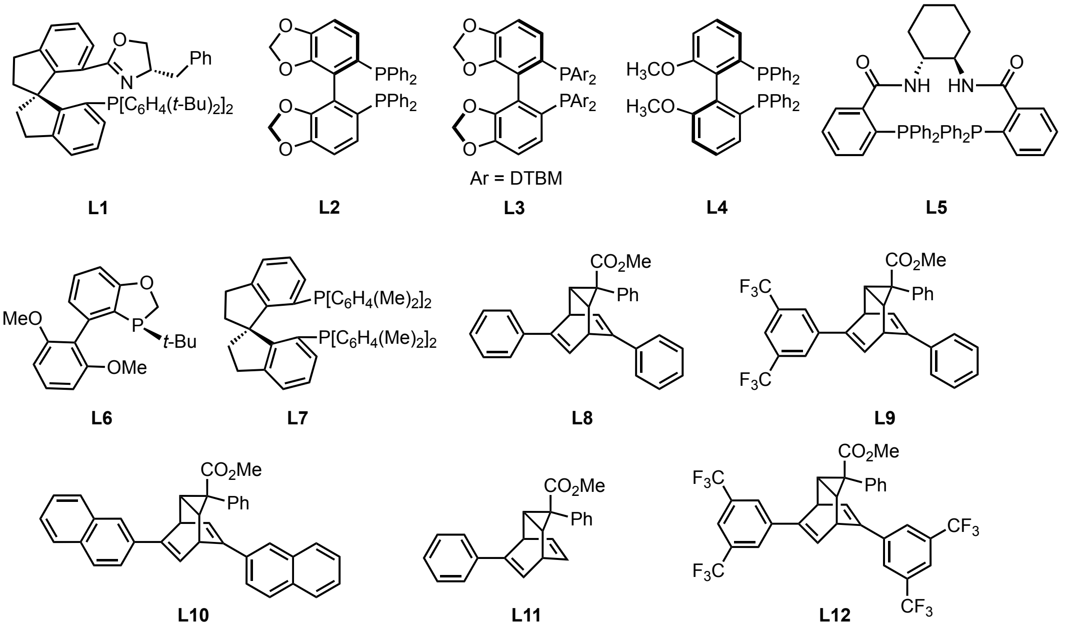

Table S2. Ligand screen

Ligand	Conversion (NMR)	M_n ($CHCl_3$, RI) (kDa)	Đ	% m
**L1**	>95	2.0	1.6	66
**L2**	>95	ND	ND	91
**L3**	>95	1.1	ND	55
**L4**	>95	0.7	ND	50
**L5**	>95	<0.5	ND	60
**L6**	>95	<0.5	ND	--
**L7**	>95	<0.5	ND	--
**L8**	>95	1.0	1.5	68
**L9**	>95	1.1	1.3	61
**L10**	>95	0.9	1.45	63
**L11**	>95	1.0	1.23	65
**L12**	>95	1.3	1.29	70

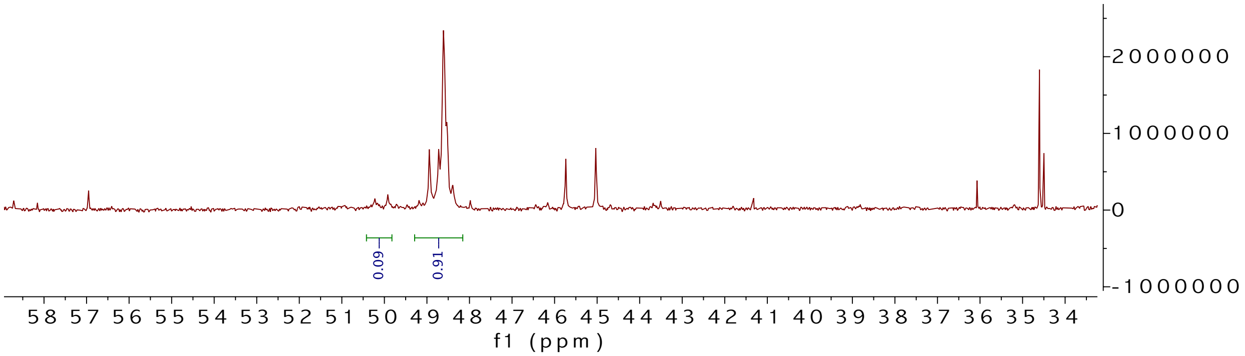

Figure S2. ^{13}C NMR of poly(**2**) in $CDCl_3$ after polymerization using Rhodium catalysis and S-BINAP. See Figure S37-39 for identification of *m* and *r* diads.

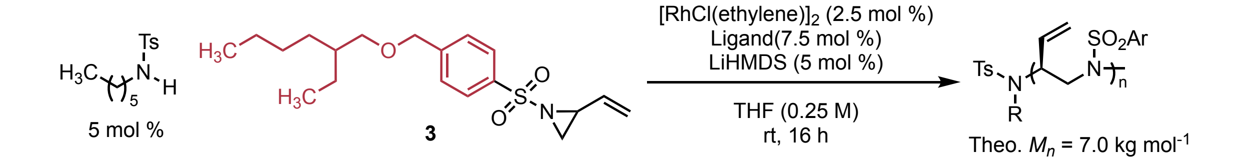

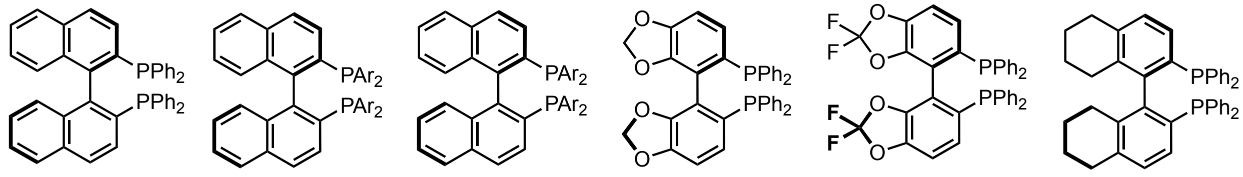

BINAP **L13**, Ar = *p*-tolyl **L14**, Ar = *m*-xylyl **L15** **L16** **L17**

Table S3. Ligand screen with **3**

Ligand	Conversion (NMR)	M_n (THF, RI) (kg mol^{-1})	Đ	% m
**BINAP**	>95	4.8	1.30	99
**L13**	>95	<0.5	ND	ND
**L14**	>95	<0.5	ND	ND
**L15**	>95	3.0	1.28	99
**L16**	>95	4.2	1.35	99
**L17**	>95	<0.5	ND	ND

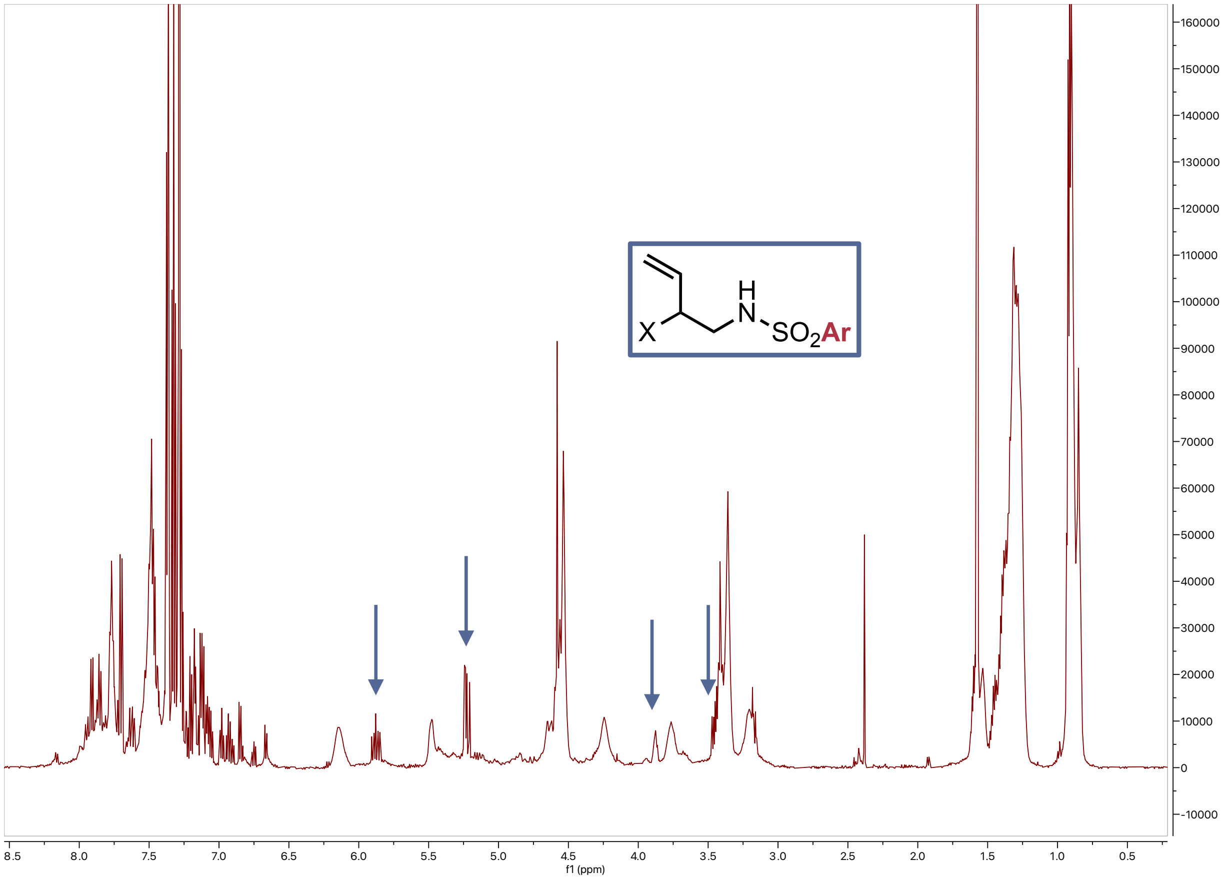

Figure S3. Crude ^{1}H NMR spectrum in $CDCl_3$ of the polymerization of **3** using Rh–BINAP in THF with LiHMDS as a base (Table 6 entry 1). Side product resonances are noted with blue arrows.

3.3 Solvent Screen

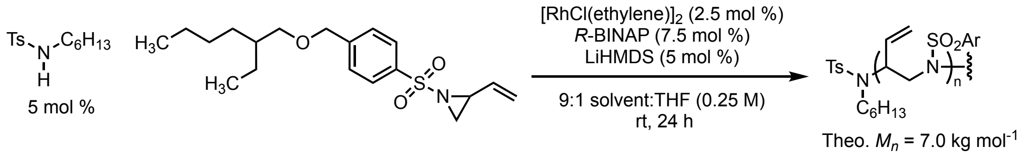

Table S4. Solvent Screen

Solvent	% Conversion	M_n (THF, RI) (kDa)	D	% m
THF	>95	3.8	1.30	99
PhCl	>95	4.3	1.35	99
Glyme	>95	3.0	--	99
Dioxane	>95	2.1	1.50	99
DCM	>95	<1.0	ND	--
MEK	>95	2.7	1.8	99
PhMe	>95	3.0	1.50	99
1:1 PhCl:PhMe	>95	4.3	1.35	99

3.4 Base screen

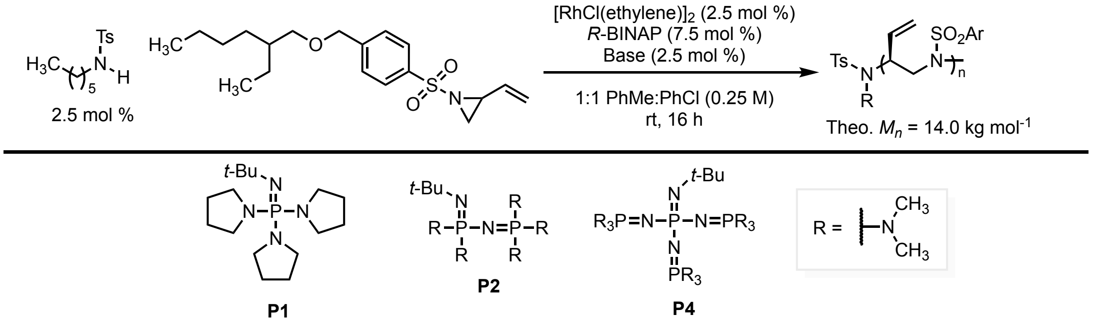

Table S5. Base screen

Base (mol %)	Conversion (NMR)	M_n (THF, RI) (kDa)	D	% m
LiHMDS	>95	6.0	1.33	99
P_1	>95	5.3	1.50	99
P_2	>95	7.4	1.42	99
P_4	>95	9.0	1.33	99

4 Polymer Synthesis and Post-polymerization Modification

4.1 Procedure for stereocnvergent polymerization

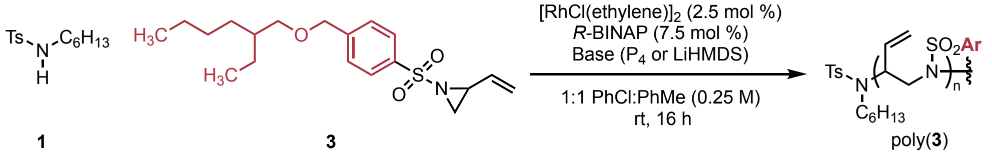

In an N_2-filled glovebox, an 8-mL vial was charged with $[Rh(C_2H_4)_2Cl]_2$ (4.8 mg/mL), BINAP (23 mg/mL), followed by solvent, and stirred for 15 min to ensure full complexation. In separate 8-mL vials with stir bars, the catalyst solution (0.375 mL) was added, followed by a solution of initiator in THF or PhMe (1 equiv relative to initiator, 0.040 mL, deprotonated with base and stirred 5 min prior to addition), and the vials stirred at room temperature for 10 min. Vinyl aziridine **3** (65 mg, 0.18 mmol) was added in a single portion as a solution in PhMe (0.375 mL), and the reaction stirred for the allotted time. Polymerizations were quenched with 1 mL of 0.1% (v/v) of TFA in MeOH and the solvent was removed under reduced pressure. A sample was analyzed by NMR to determine conversion, and the polymer was dissolved in 1 mL CH_2Cl_2 and precipitated into cold MeOH, and dried via vacuum to furnish pure polymer samples for analysis by SEC, TGA, and DSC, optical rotation and CD.

^{1}H NMR (400 MHz, $CDCl_3$) δ 8.12 – 7.68, 7.59 – 7.37, 6.37 – 5.72 , 5.58 – 4.92, 4.70 – 4.44, 4.26, 3.92 – 3.58, 3.46 – 3.29, 3.27 – 3.08, 1.54, 1.46 – 1.18, 0.99 – 0.74.

^{13}C NMR (101 MHz, $CDCl_3$) δ 144.7, 137.8, 132.9, 128.0, 127.7, 127.2, 124.7, 123.4, 73.8, 72.0, 58.8, 50.4, 49.0, 39.7, 30.6, 29.1, 23.9, 23.1, 14.2, 11.1.

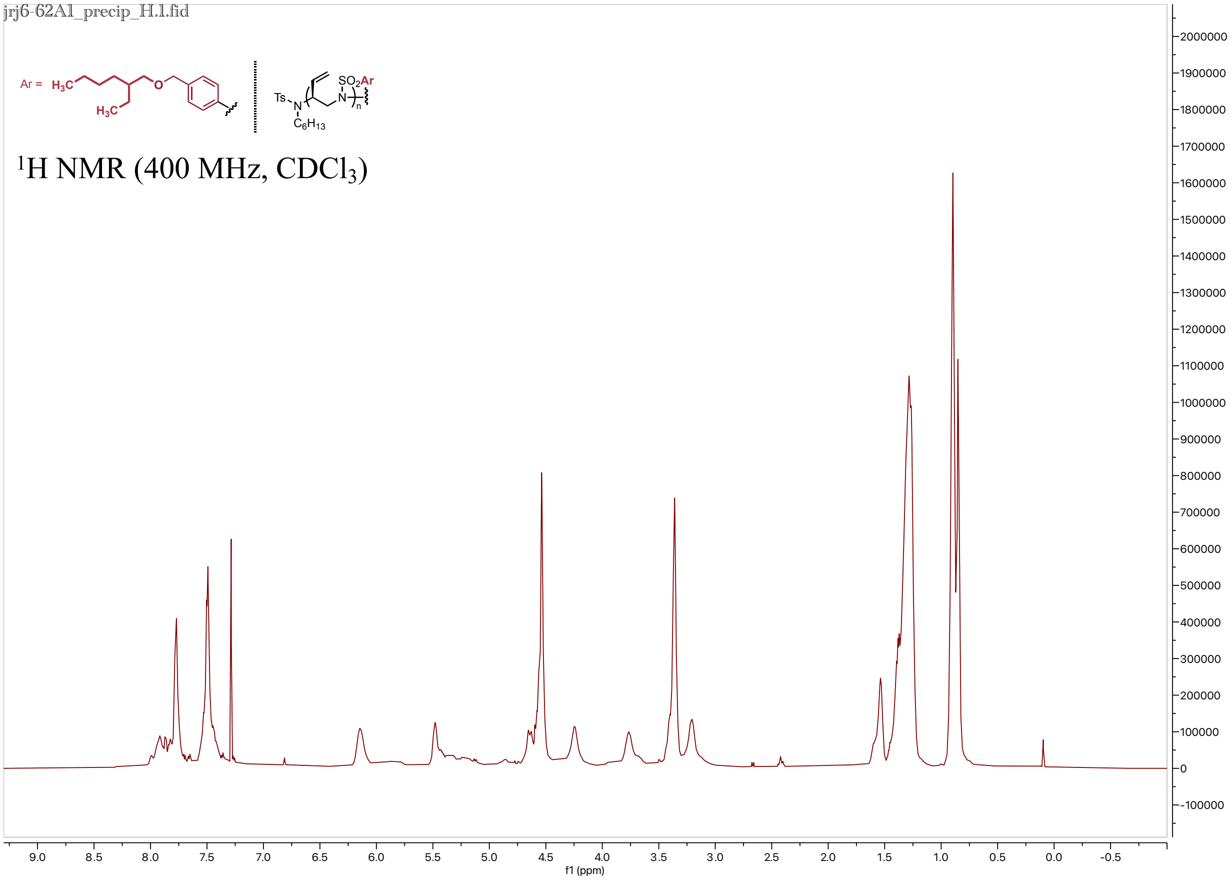

Figure S4. Representative ^{1}H NMR spectrum of isotactic poly(**3**) in $CDCl_3$.

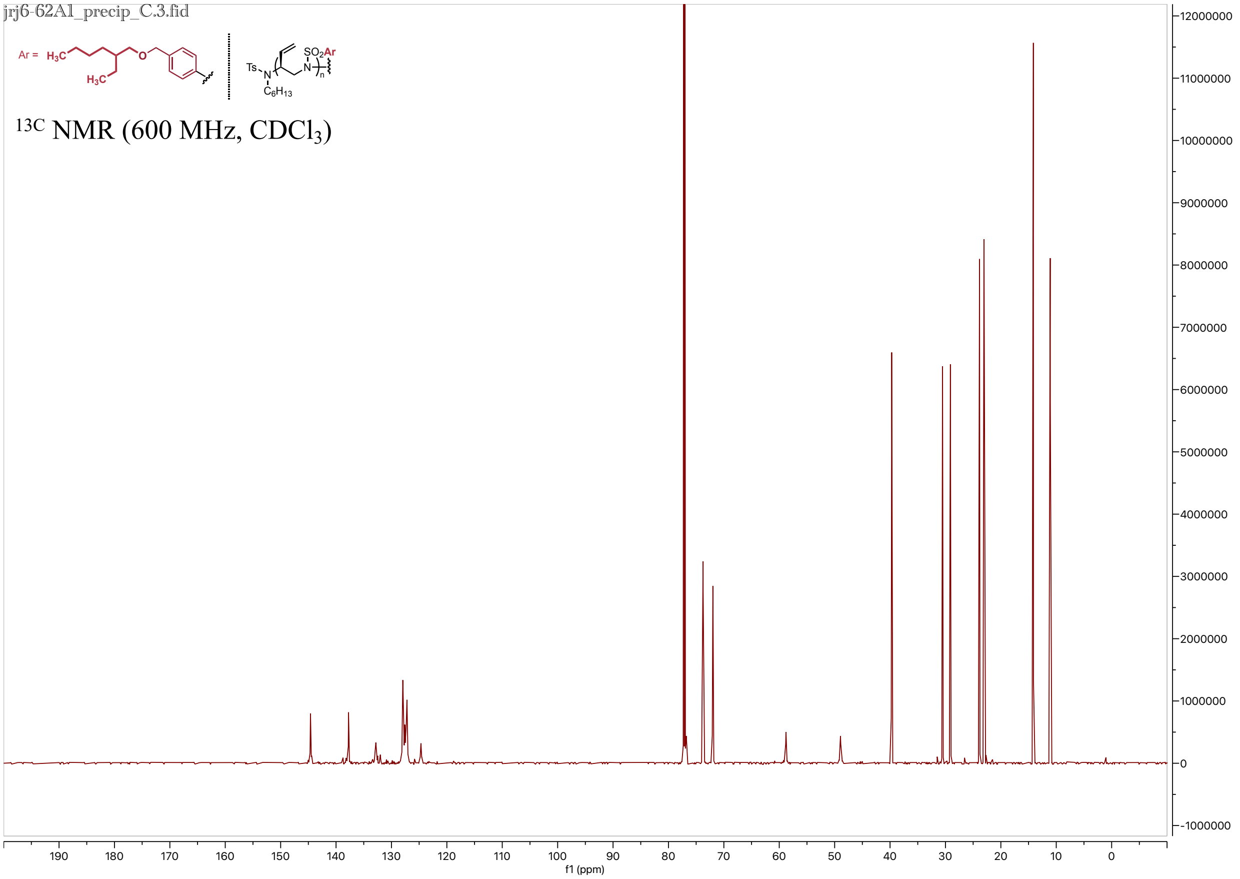

Figure S5. Representative ^{13}C NMR spectrum of poly(**3**) in $CDCl_3$.

Table S6. SEC data based on target molar mass

Target M_n (kg•mol^{-1})	Mol % **1**	M_n THF, RI (kg mol^{-1})	Đ	% *m*
3.5	10.0	3.9	1.26	99
7.0	5.0	6.3	1.30	99
10.5	3.3	8.3	1.30	99
14.0	2.5	10.0	1.27	99
21.0	1.8	16.5	1.52	99
28.0	0.6	12.7	2.32	99
No initiator	0	5.0	1.30	ND

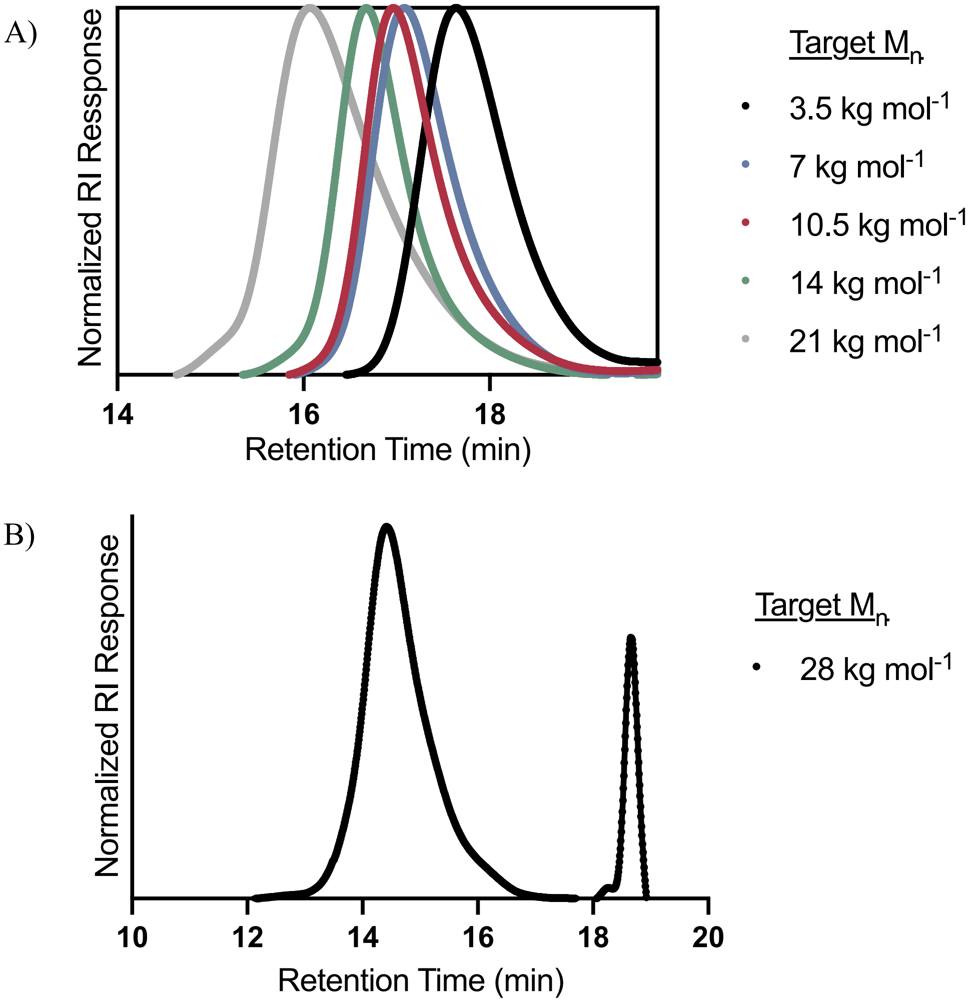

Figure S6. SEC traces of poly(**3**) targeting between A) 3.5-21 kg mol^{-1} and B) M_n = 28.0 kg mol^{-1}. Data was collected on two instruments (Tosoh GPC for A) and Agilent for B), *vide supra*) but in identical solvents (THF) using polystyrene calibrants.

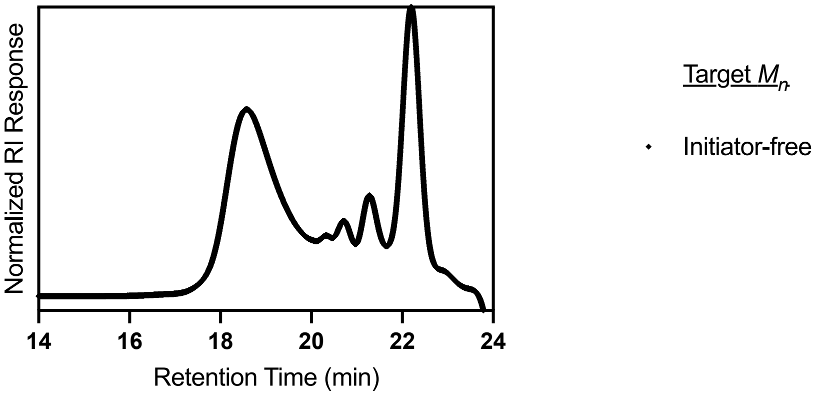

Figure S7. SEC traces of poly(**3**) under initiator-free conditions using an Agilent GPC (*vide supra*). M_n = 5.0 kg mol^{-1}, Đ = 1.30.

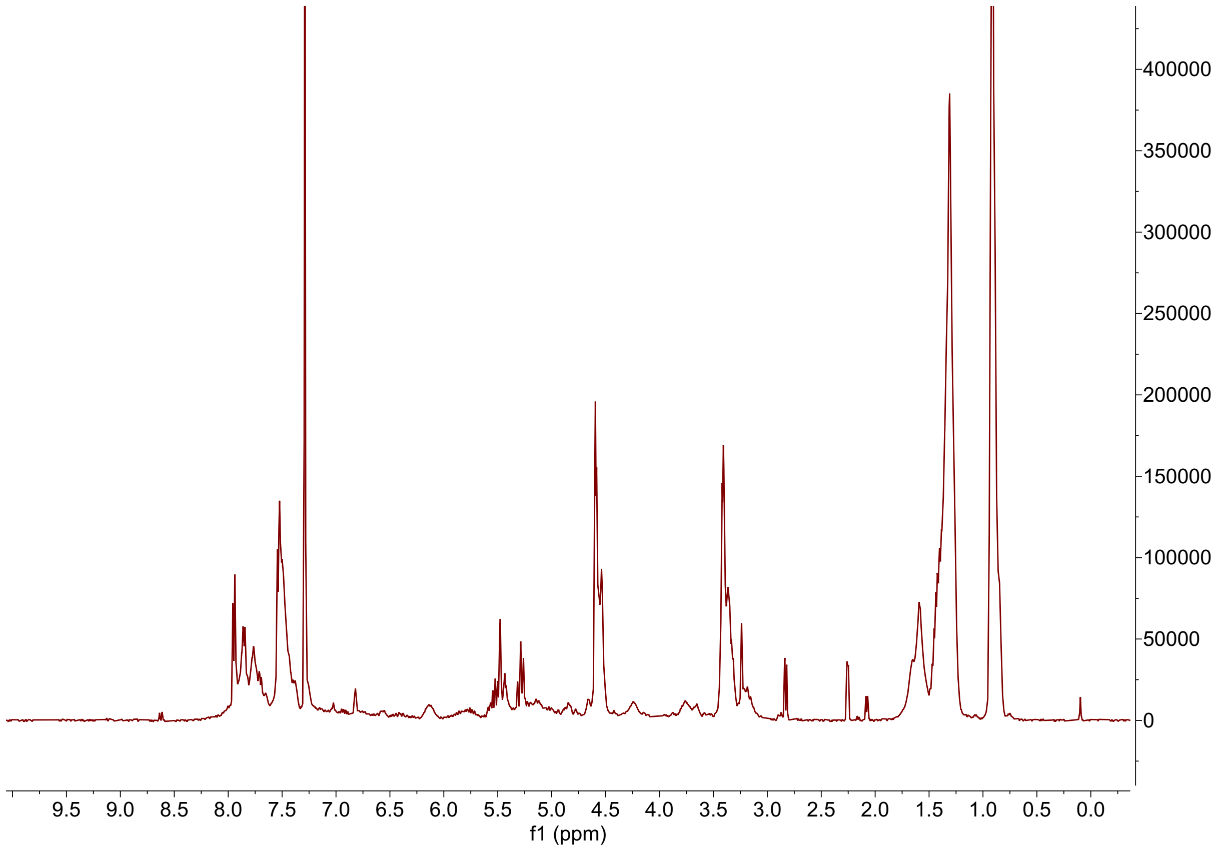

Figure S8. Crude ^{1}H NMR spectrum in $CDCl_3$ of the polymerization of **3** using Rh–BINAP in 1:1 PhCl:PhMe in the absence of initiator **2** and base.

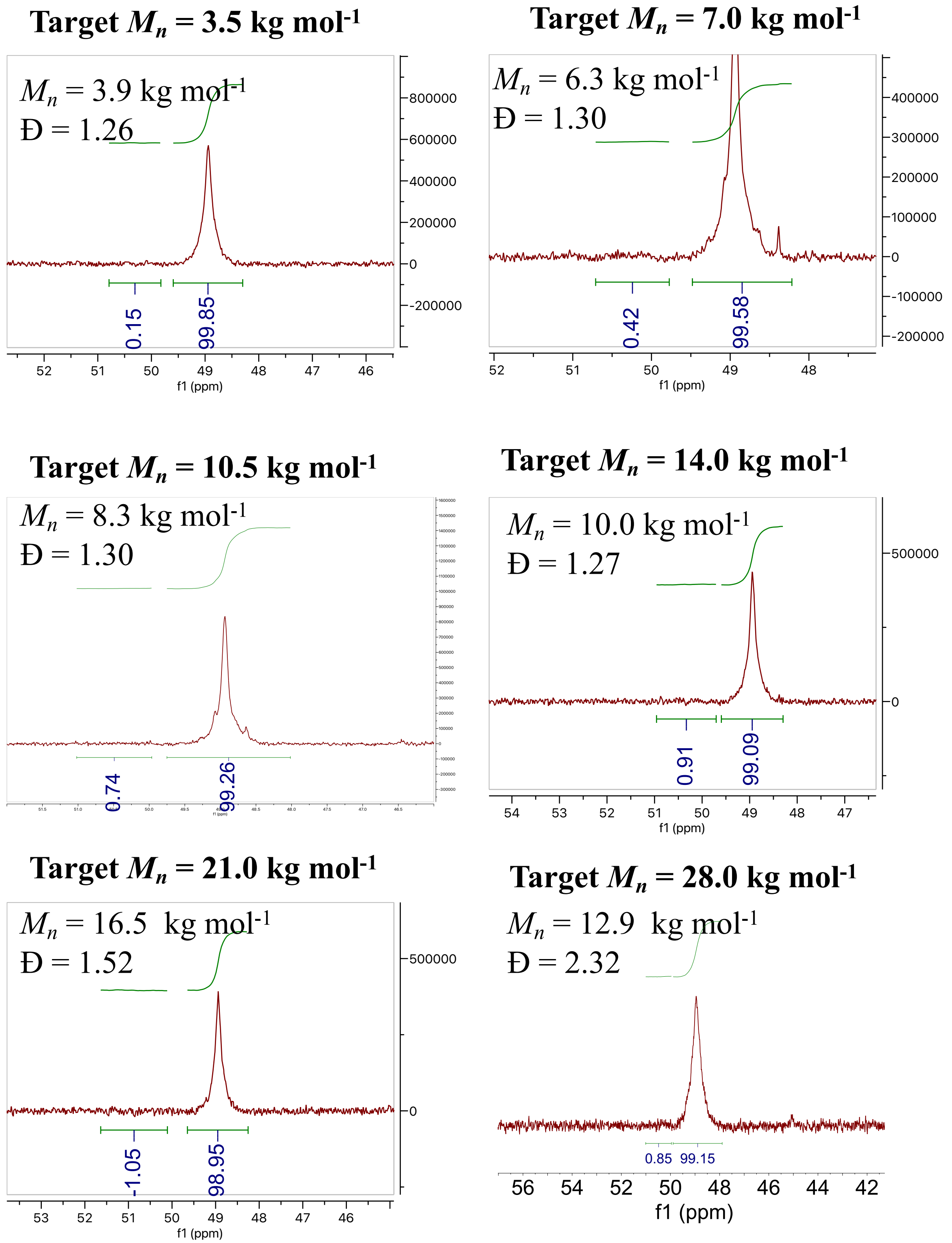

Figure S9. Isotacticity calculations of poly(**3**)s based on target molar mass with *rac*-**3**. All spectra were acquired with 8192 scans and corrected with splines prior to analysis.

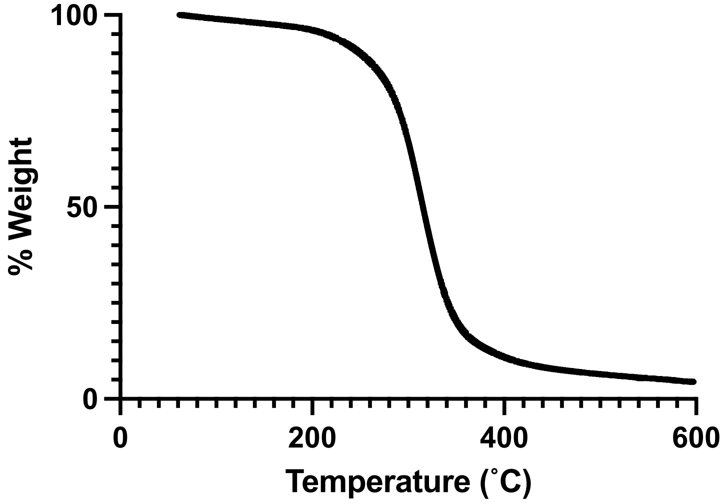

Figure S10. TGA trace of poly(**3**).

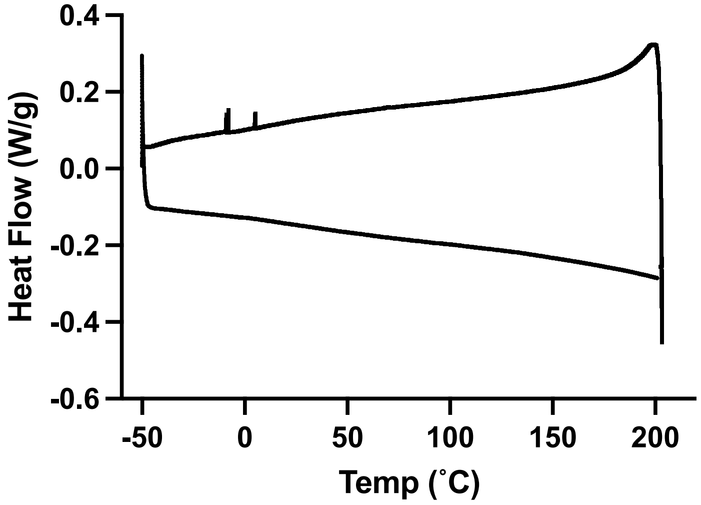

Figure S11. DSC trace of poly(**3**) using a ramp rate of 10˚C/min. No thermal transitions were observed.

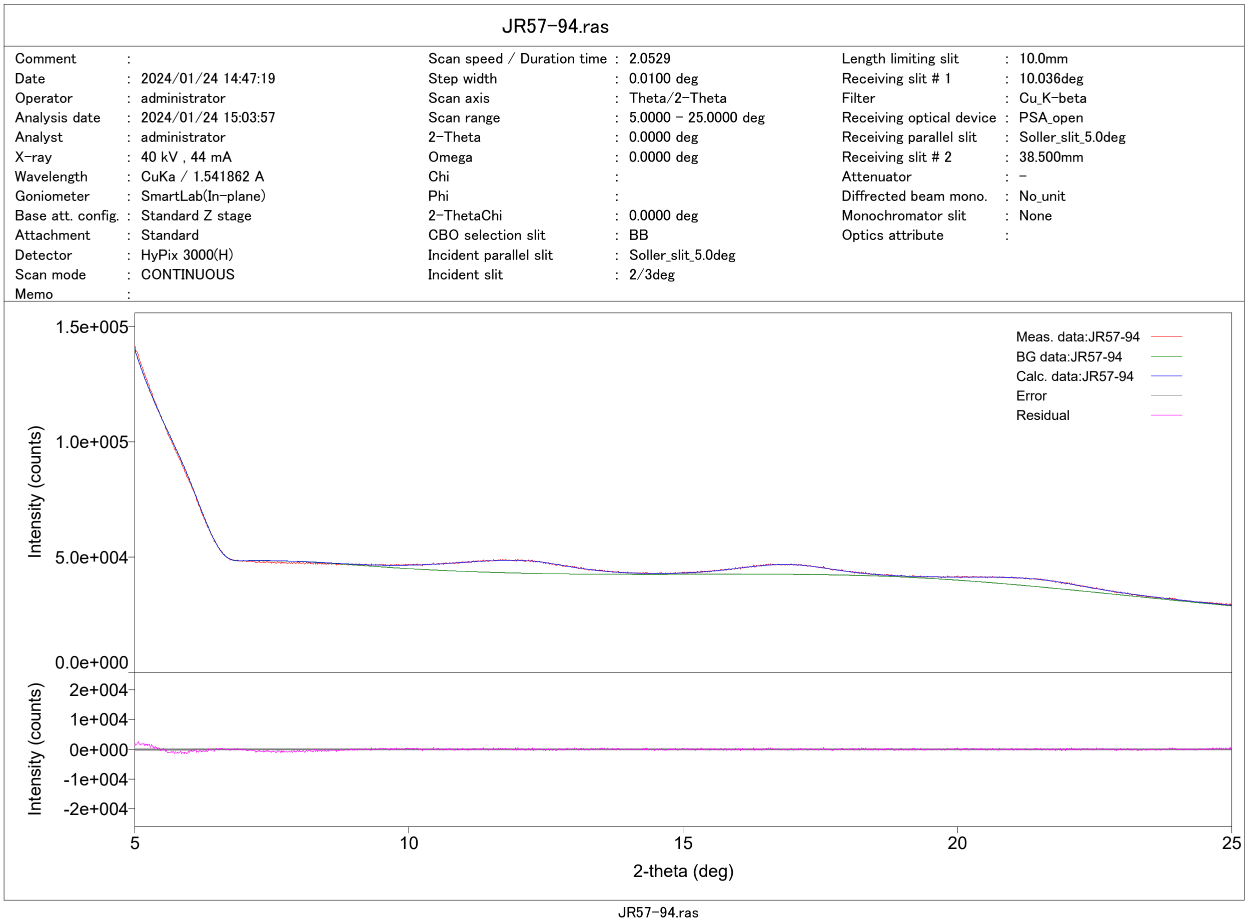

Figure S12. PXRD of poly(**3**) (ethyl hexyl). No evidence of crystallinity was observed

4.2 Procedure for Chain-extension

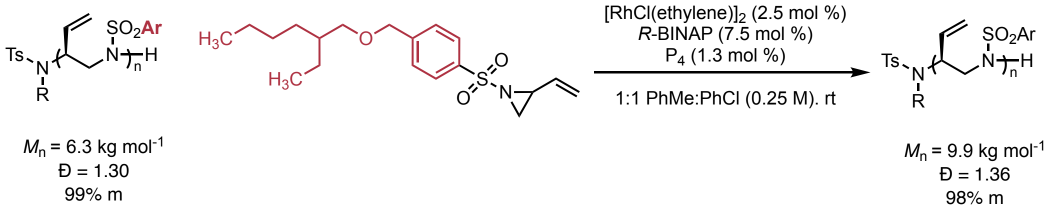

In an N_2-filled glovebox, an 8-mL vial was charged with $[Rh(C_2H_4)_2Cl]_2$ (9.6 mg/mL), BINAP (46 mg/mL), followed by PhCl, and stirred for 15 min to ensure full complexation. In separate 8-mL vial with a stir bar, poly(**3**) (65 mg, 0.18 mmol repeat unit) was added, followed by P_4 (5 µL of a 0.8 M solution in hexanes) and stirred for 10 min. To the vial with polymer and P4, catalyst solution (0.18 mL) was added, followed by a solution of monomer (0.18 mmol in 0.375 mL PhMe) and the reaction stirred for 16 h. Polymerizations were quenched with 1 mL of 0.1% (v/v) of TFA in MeOH and the solvent was removed under reduced pressure. A sample was analyzed by NMR to determine conversion, and the polymer was dissolved in 1 mL CH_2Cl_2 and precipitated into cold MeOH, and dried via vacuum to furnish pure polymer samples for analysis by NMR, SEC, and CD.

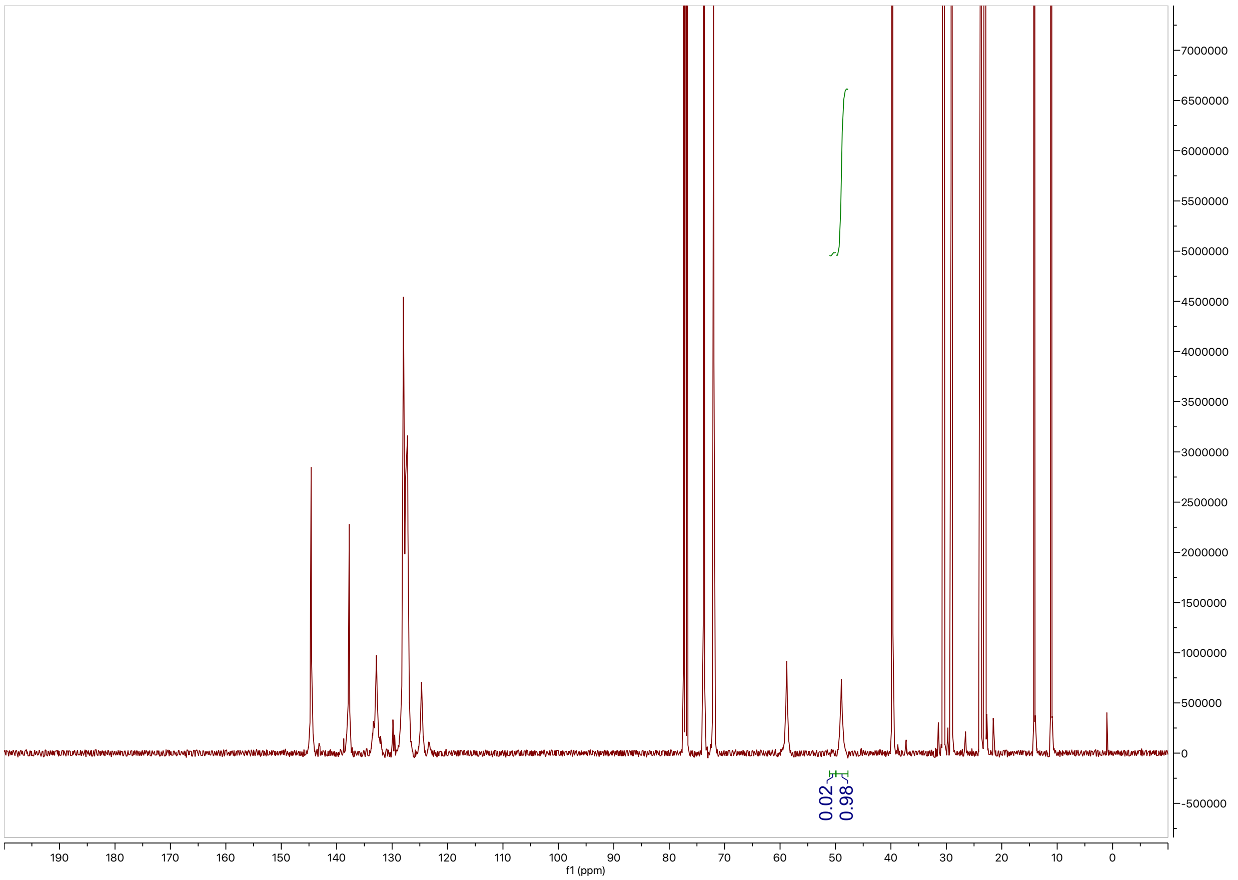

Figure S13. Tacticity calculation of poly(**3**) after chain-extension.

4.3 Hydrosilylation (poly(6))

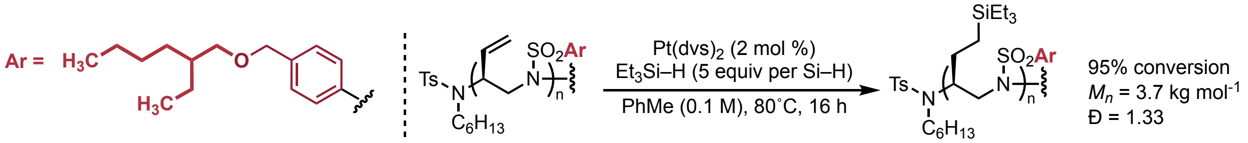

A flame-dried 20-mL scintillation flask with a stir bar was charged with poly(**3**) (100 mg, 0.28 mmol repeat unit), and purged with N_2 for 5 min. To the vial was added PhMe (3 mL, 0.1 M concentration), triethylsilane (0.2 mL, 5.0 equiv per alkene), followed by Karstedt's catalyst (0.08 mL of a 2 wt% solution in xylenes). The reaction was heated to 80˚C and stirred for 16 h. The reaction was allowed to cool to room temperature, and the solvent was removed via rotary evaporator. The crude reaction mixture was precipitated twice from CH_2Cl_2/cold MeOH to furnish poly(**6**) as a brown solid which was analyzed by NMR, GPC and CD.

^{1}H NMR (400 MHz, $CDCl_3$) δ 8.15-7.63, 7.59-7.37, 4.65-4.45, 4.40, 4.13-3.95, 3.51-3.36, 3.28-3.02, 2.35-1.18, 1.07-0.25.

^{13}C NMR (151 MHz, $CDCl_3$) δ 144.5, 138.2, 132.9, 127.8, 127.4, 124.6, 73.7, 71.9, 61.8, 58.6, 50.8, 49.0, 47.6, 39.7, 30.5, 29.1, 23.8, 23.0, 15.0, 11.1, 7.4, 6.6, 5.8, 3.1, 1.0, -0.5.

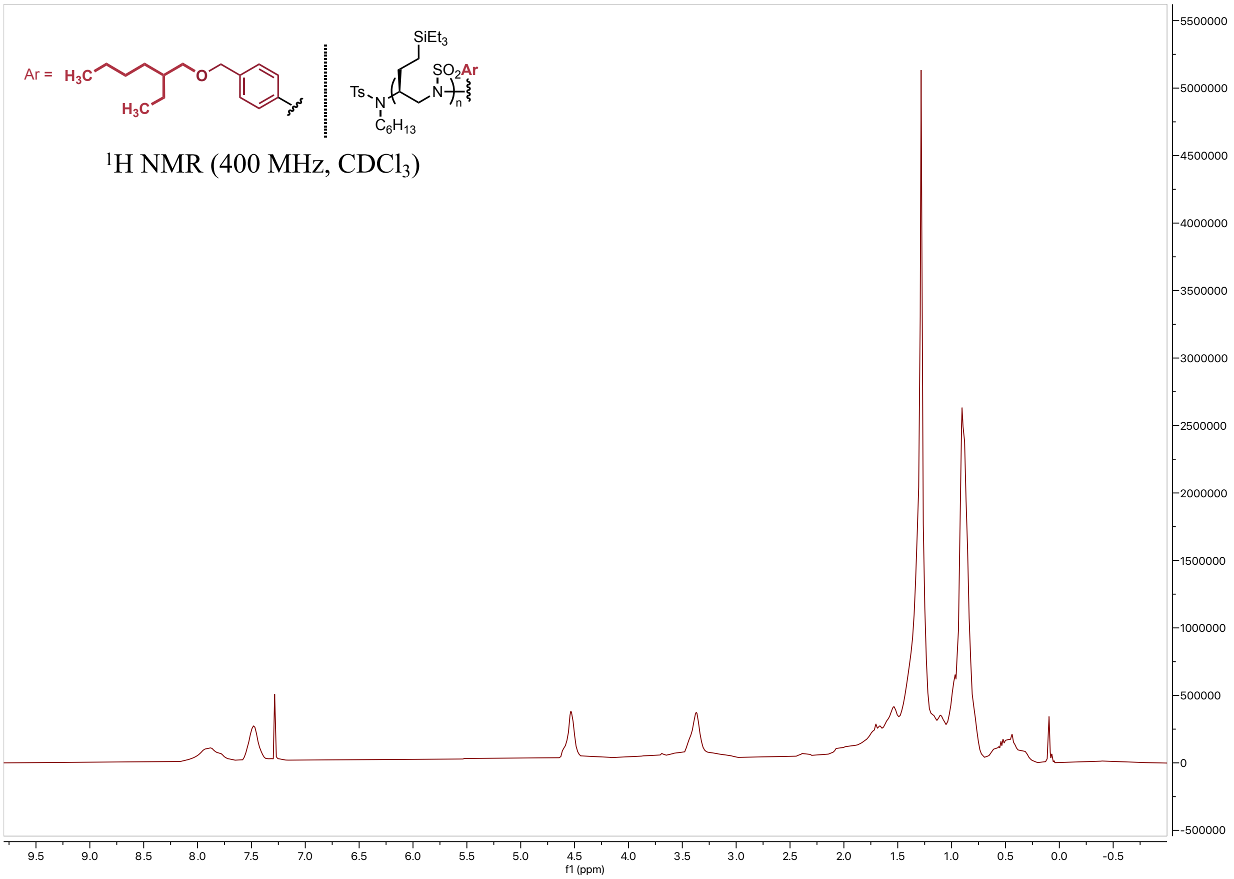

Figure S14. ^{1}H NMR spectrum of poly(**6**) in $CDCl_3$.

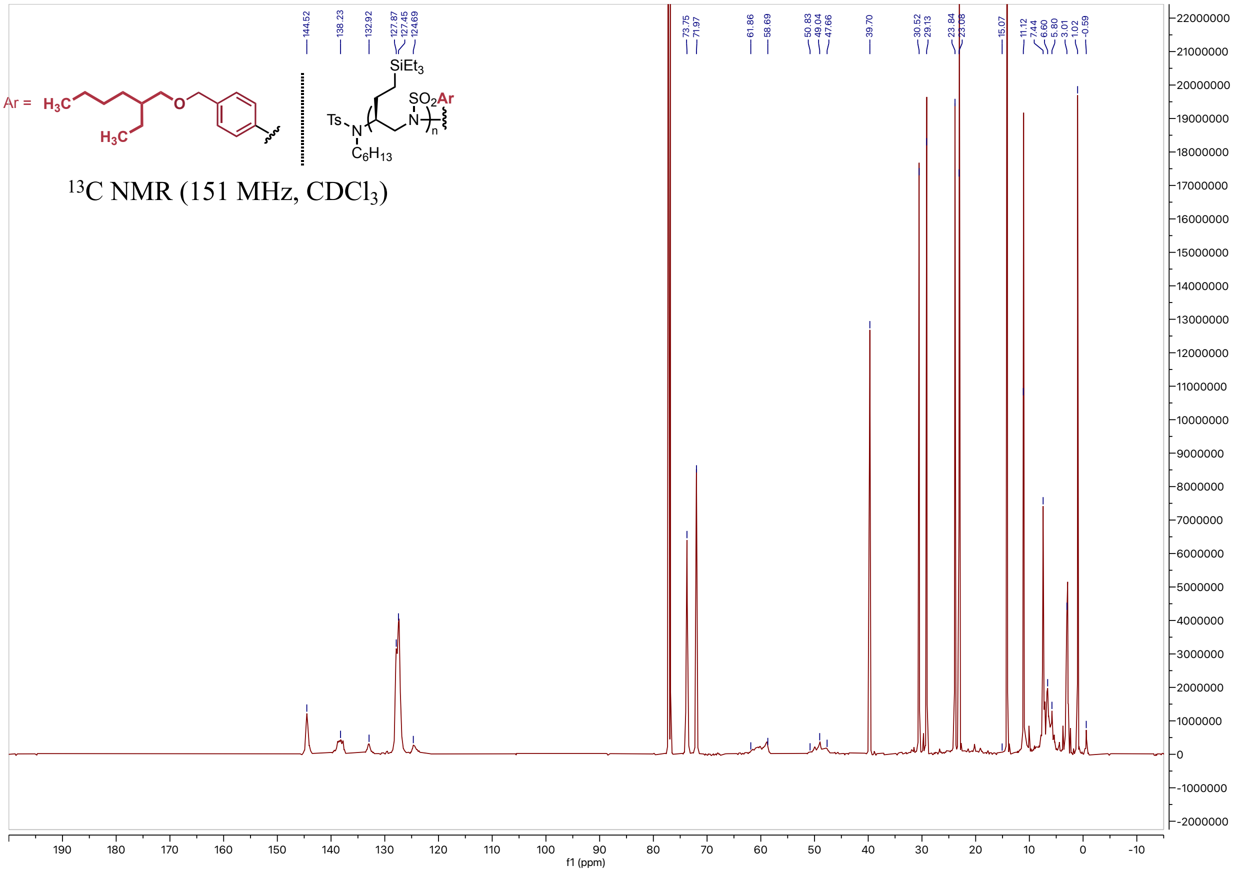

Figure S15. ^{13}C NMR spectrum of poly(**6**) in $CDCl_3$.

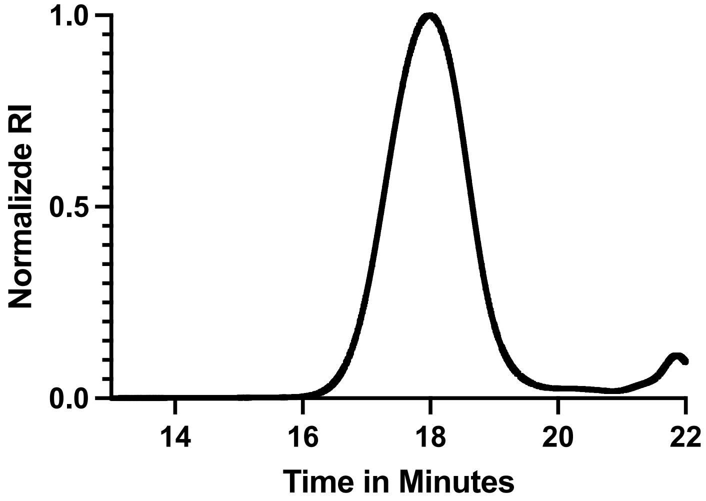

Figure S16. SEC traces of poly(**6**) in THF.

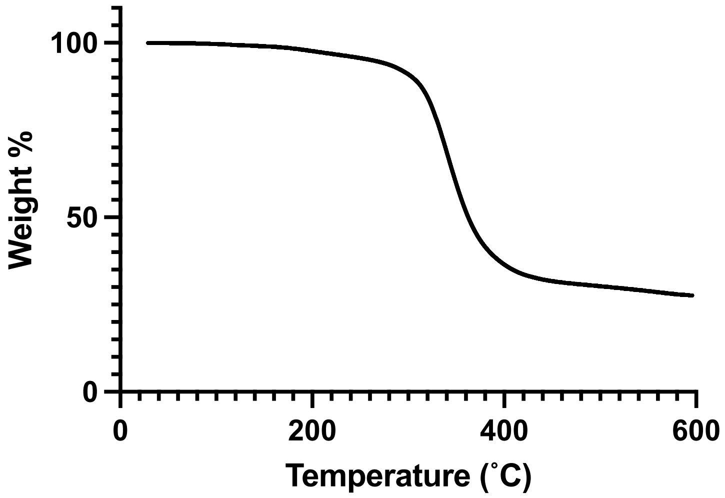

Figure S17. TGA of isotactic poly(**6**) using a ramp rate of 20˚C/min.

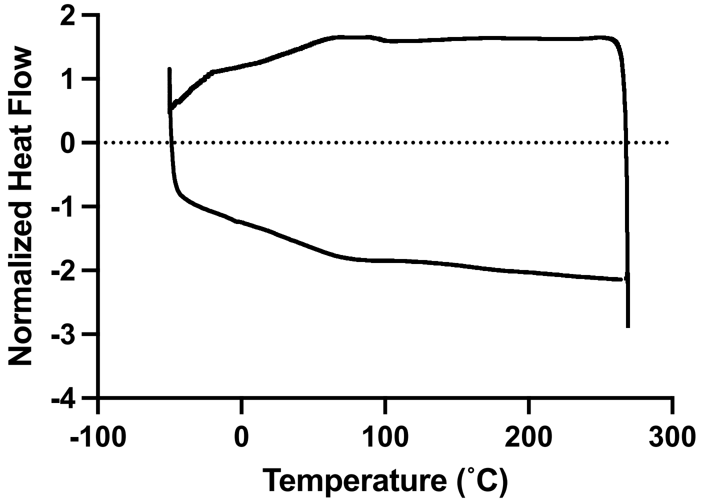

Figure S18. DSC of isotactic poly(**6**) using a ramp rate of 20˚C/min after removal of thermal history. T_g = 75˚C; T_m = not observed

4.4 Thiol-ene click (poly(7))

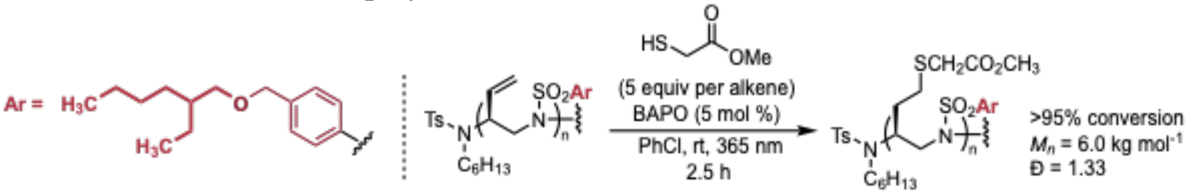

A flame-dried 4-mL reaction vial with a stair bar was charged with poly(**3**) (100 mg, 0.28 mmol repeat unit), followed by BAPO (11.9 mg, 10 mol %), methylthioglyclate (0.51 mL, 5.70 mmol, 20 equiv per repeat unit), followed by PhCl (0.2 mL). The reaction stirred at room temperature until all components were dissolved and the reaction was sparged with nitrogen for 30 min to remove dissolved oxygen. After sparging, the reaction was placed in an Asiga flash cure box and irradiated at 385 nm for 2.5 h with stirring. After irradiation, the reaction contents were precipitated into cold hexanes and the poly(**7**) was collected and dried under vacuum to furnish pure samples for NMR, SEC and CD.

^{1}H NMR (400 MHz, $CDCl_3$) δ 8.27- 7.75, 7.68- 7.12, 4.73-4.31, 3.85-3.52, 3.49-3.20, 3.15-3.00, 2.75-1.86, 1.71- 1.15, 0.99-0.71.

^{13}C NMR (101 MHz, $CDCl_3$) δ 170.5, 144.7, 138.0, 127.6, 73.8, 71.9, 55.2, 52.4, 39.7, 32.9, 31.3, 30.5, 29.1, 26.6, 23.8, 23.0, 14.1, 11.1.

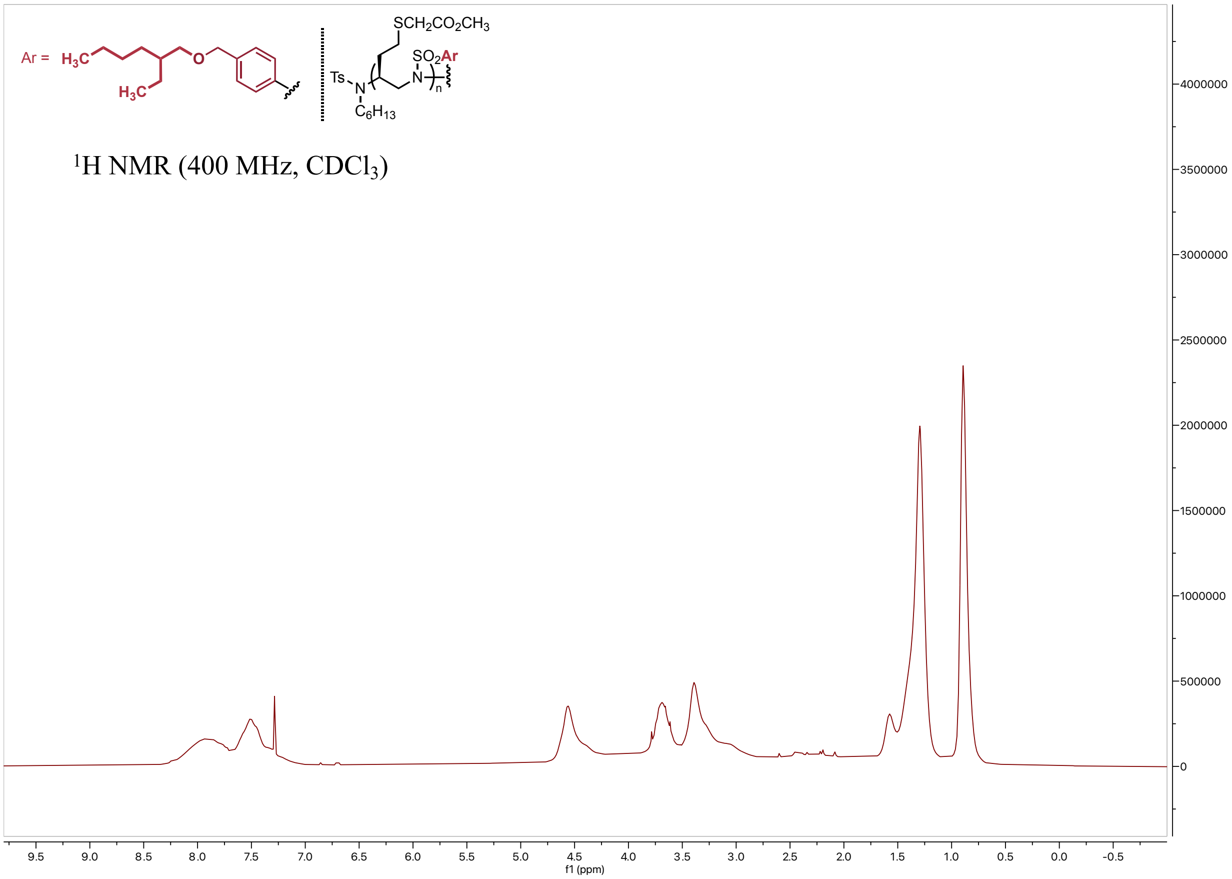

Figure S19. ^{1}H NMR spectrum of poly(**7**) in $CDCl_3$.

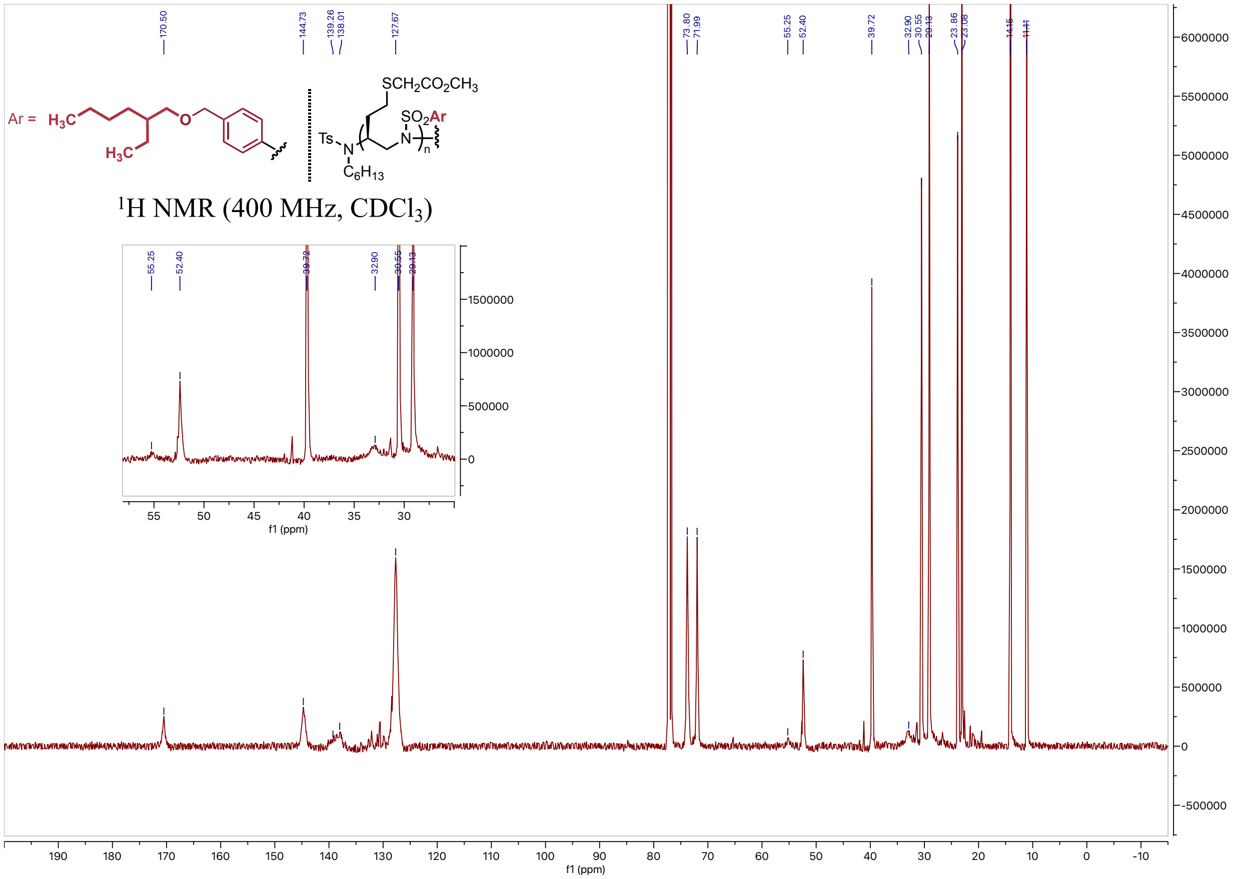

Figure S20. ^{13}C NMR spectrum of poly(**7**) in CDCl$_3$.

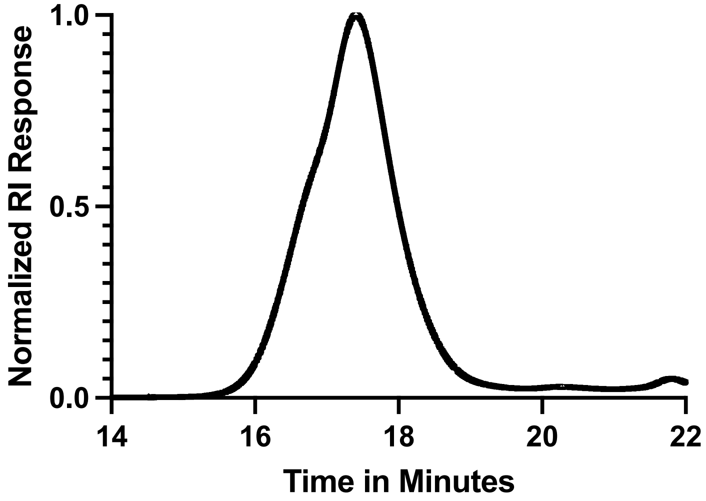

Figure S20. SEC traces of poly(**7**) in THF.

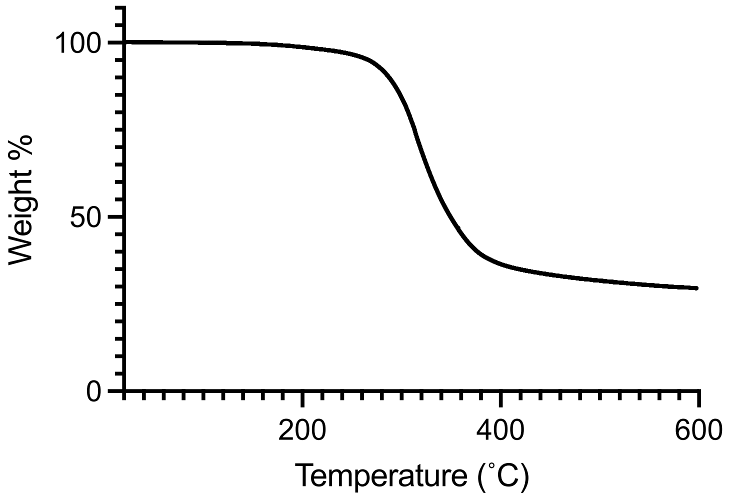

Figure S21. TGA trace of poly(**7**).

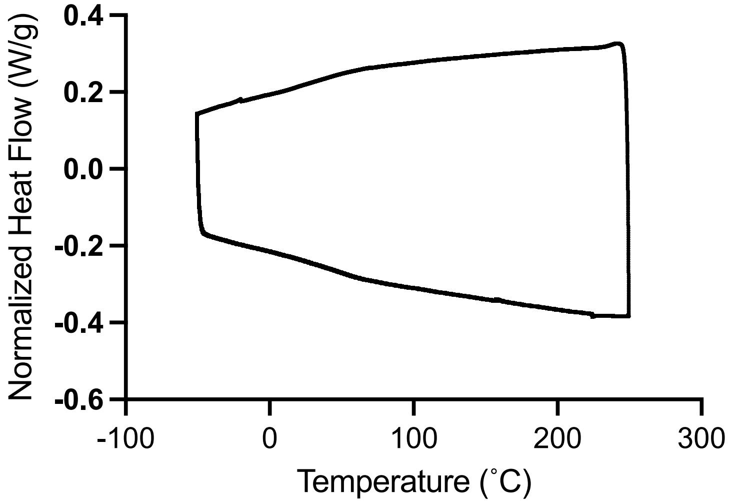

Figure S22. DSC traces of poly(**7**).

4.5 Alkene Reduction (poly(8))

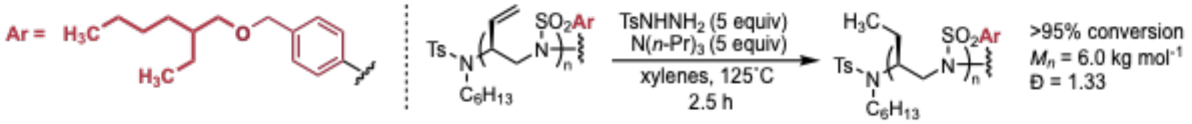

A flame-dried 20-mL scintillation vial with a stirbar was charged with poly(XX) (125 mg, 0.35 mmol repeat unit), xylenes (4.2 mL, 0.08M), and tripropylamine (0.8 mL, 12 equiv relative to repeat unit), BHT (1 mg) and the reaction was sparged with nitrogen for 30 min. After sparging, the reaction was heated to 125˚C and tosylhydrazine (650 mg total, 3.50 mmol, 10 equiv per repeat unit) was added in 10 portions with each portion added every 15 minutes (65 mg per portion for a total of 650 mg). After the final portion was added the reaction was allowed to cool to room temperature and the solvent was removed via rotary evaporator. The crude contents were dissolved in CH_2Cl_2 (3 mL) and precipitated into cold MeOH (50 mL) twice and dried under vacuum to furnish pure poly(**8**) for NMR, SEC, TGA, DSC and CD.

^{1}H NMR (400 MHz, $CDCl_3$) δ 8.18-7.82, 7.60-7.39, 4.64-4.46, 3.90-3.52, 3.41-3.18, 2.27-1.85, 1.64-1.47, 1.45-1.17, 0.97-0.76, 0.52-0.15.

^{13}C NMR (151 MHz, $CDCl_3$) δ 144.6, 138.4, 128.0, 127.7, 127.2, 73.7, 72.1, 59.7, 48.8, 39.7, 30.6, 29.1, 23.9, 23.1, 18.6, 14.2, 11.1, 10.8.

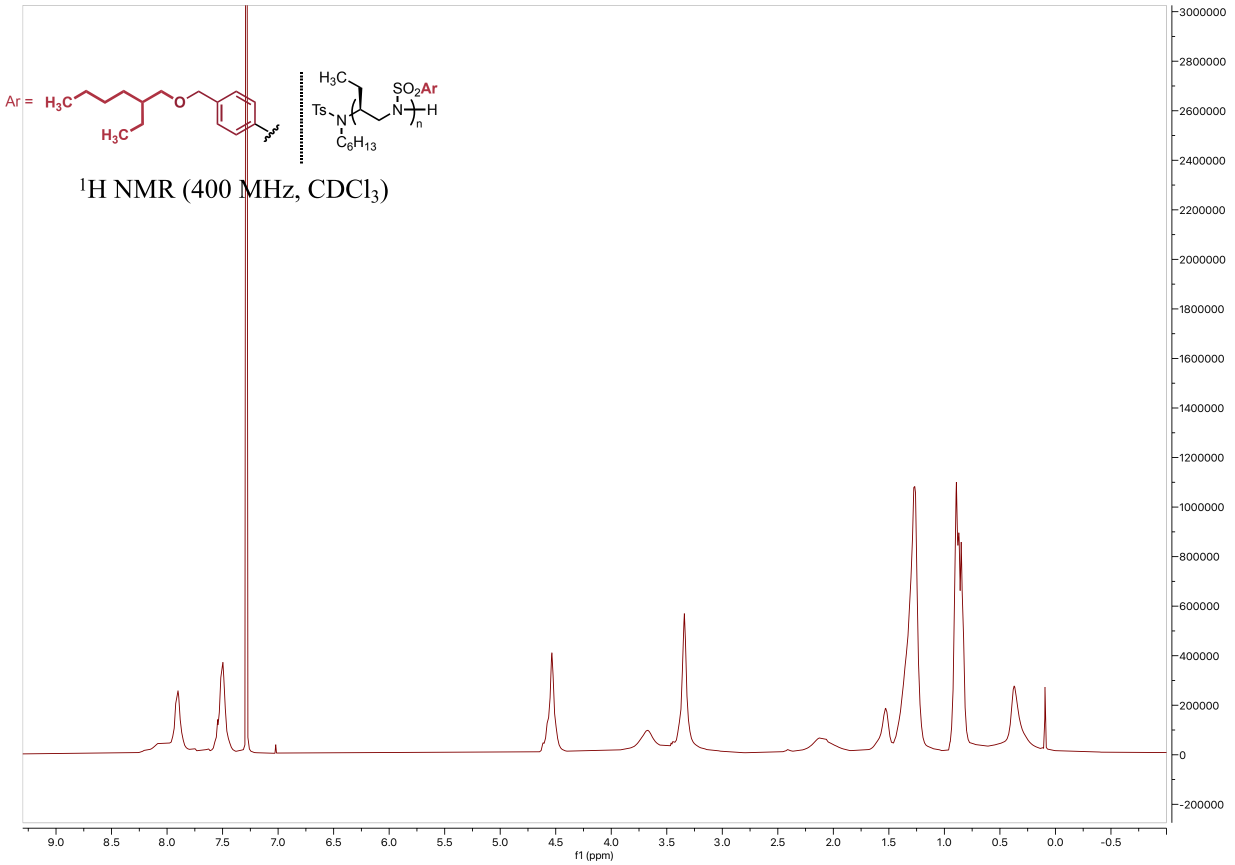

Figure S23. ^{1}H NMR spectrum of isotactic poly(**8**) in $CDCl_3$.

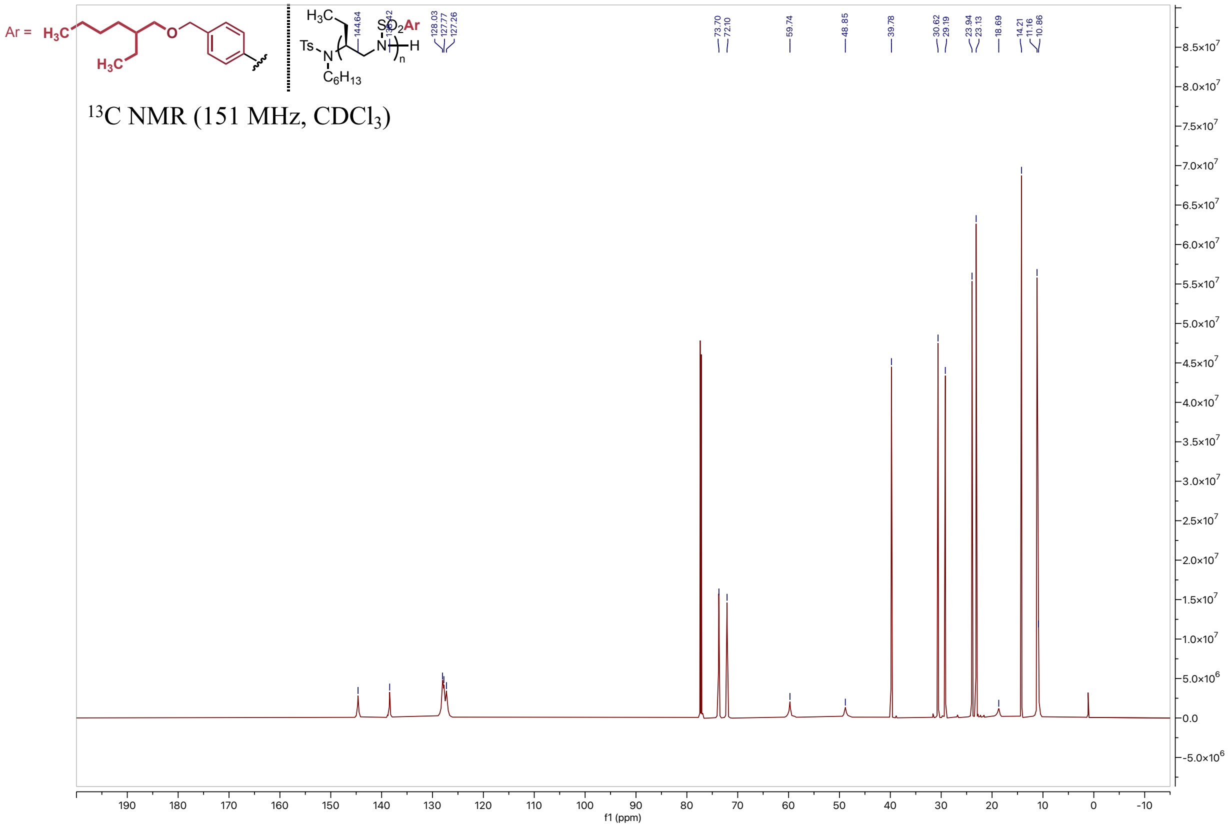

Figure S24. ^{13}C NMR spectrum of isotactic poly(**8**) in $CDCl_3$.

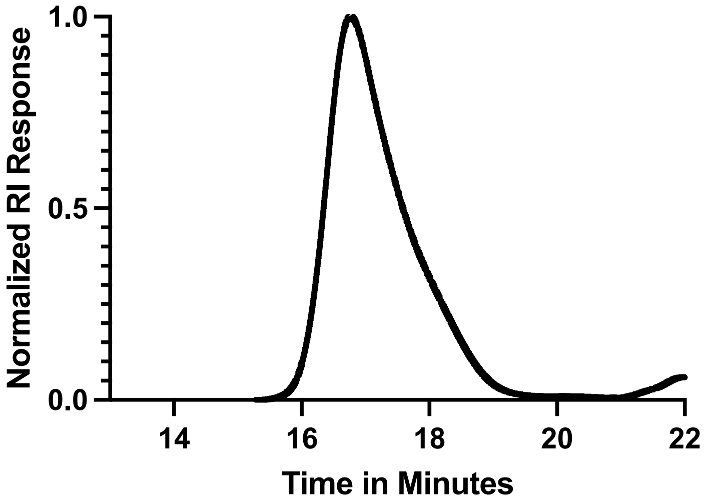

Figure S25. SEC traces of poly(**8**) in THF.

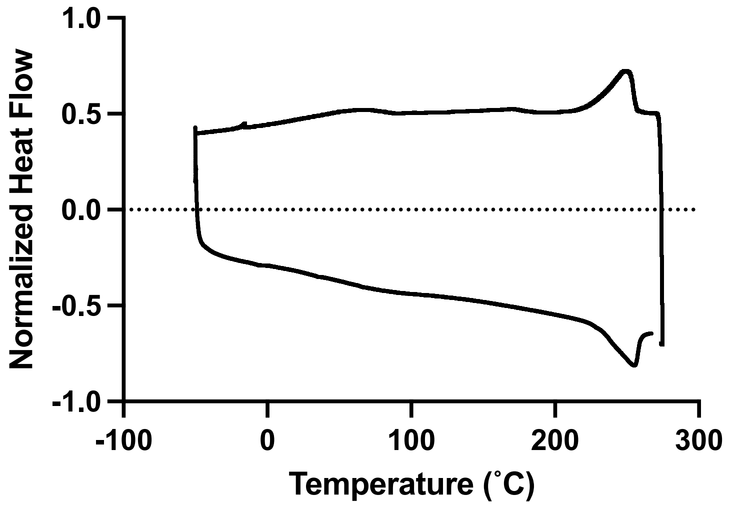

Figure S26. DSC of isotactic poly(**8**) using a ramp rate of 10˚C/min after removal of thermal history. T_g = 90˚C; T_m = 252˚C

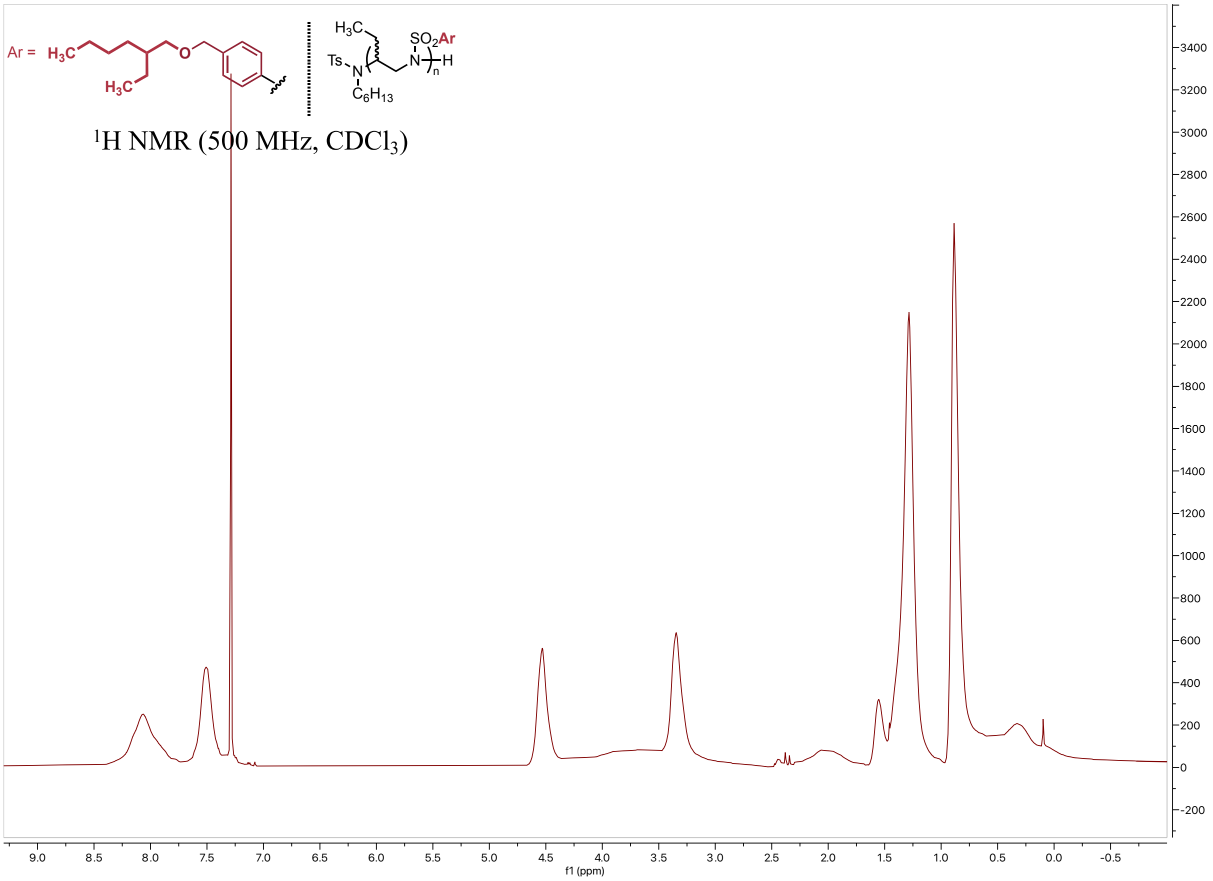

Figure S27. ^{1}H NMR spectrum of atactic poly(**8**) in $CDCl_3$.

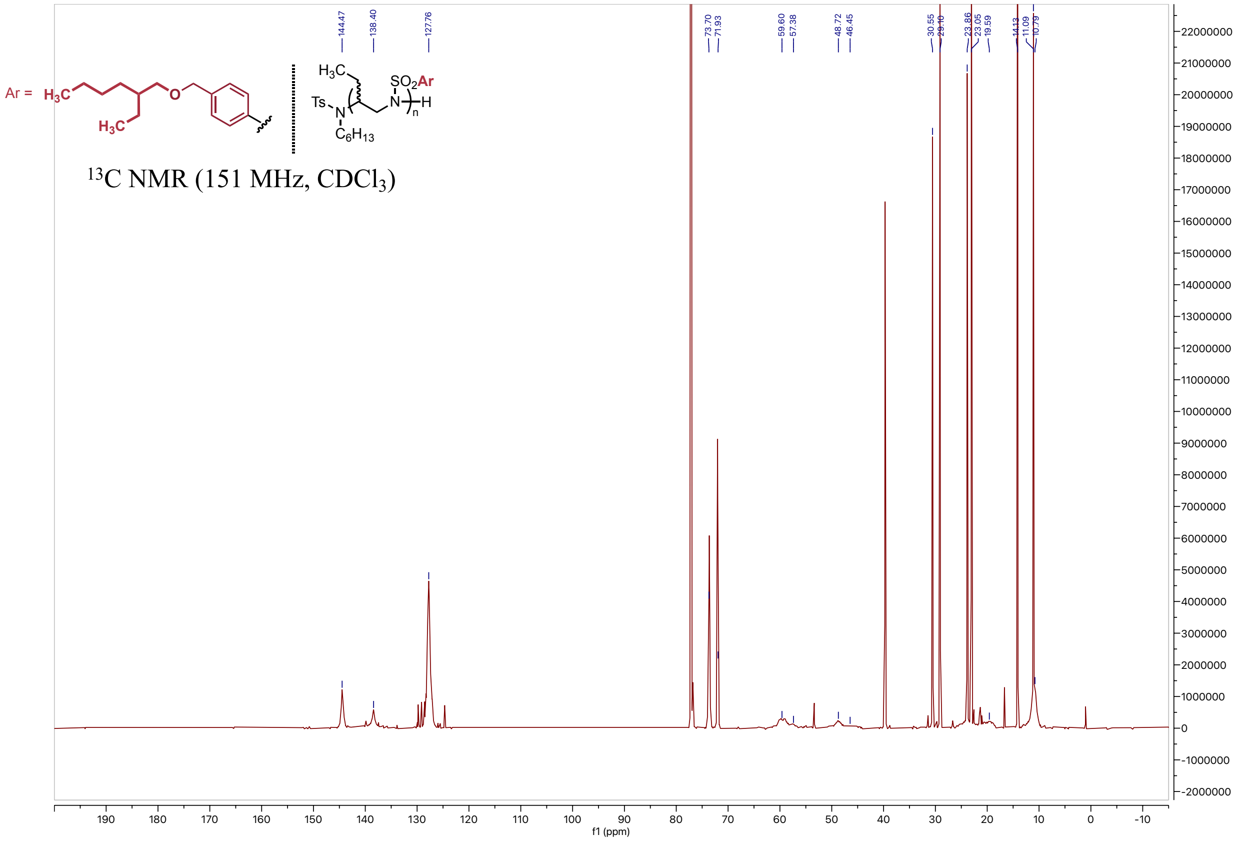

Figure S28. ^{13}C NMR spectrum of isotactic poly(**8**) in $CDCl_3$.

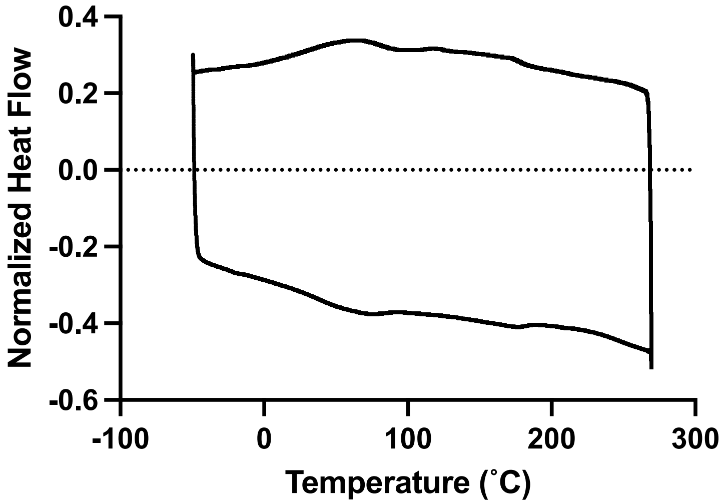

Figure S29. DSC of atactic poly(**8**) using a ramp rate of 10˚C/min after removal of thermal history. T_g = 90˚C, M_n = 10.0 kg mol^{-1}, Đ = 1.20.

4.6 C–H Oxidation (poly(9))

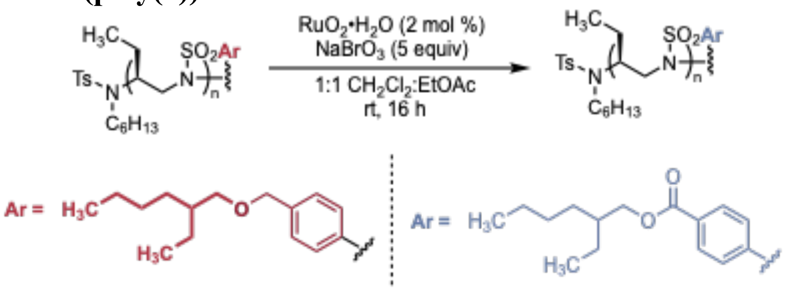

A 20-mL scintillation vial with a stir bar was charged with poly(**3**) (100 mg, 0.26 mmol repeat unit), followed by EtOAc and CH_2Cl_2 (1 and 0.5 mL, respectively). A solution of $NaBrO_3$ (0.400 g, 2.60 mmol, 10.0 equiv per repeat unit) in water (1.5 mL) was then added to the vial, leading to a biphasic solution. Vigorous stirring was initiated (1400 rpm) to maximize contact of the two phases and RuO_2 (2.5 mg, 0.01 mmol, 4 mol %) was added in single portion. The reaction stirred at 23˚C until the solution became a persistent yellow (~16h). After stirring, the organic layer was separated from the aqueous layer and quenched with a solution of saturated sodium bisulfite (2 mL). The organic layer was washed with brine, separated from the aqueous layer and dried with Na_2SO_4, filtered, and the solvent was removed via rotary evaporator. A sample was taken for NMR and SEC to determine conversion and molar mass and the polymer was further purified via precipitation with CH_2Cl_2/MeOH twice to furnish pure poly(**9**) samples for further characterization.

^{1}H NMR (600 MHz, $CDCl_3$) δ 8.34- 7.84, 4.40-4.13, 3.92-3.15, 2.30-1.92, 1.81-1.58, 1.53-1.16, 1.05-0.77, 0.59-0.20.

^{13}C NMR (151 MHz, $CDCl_3$) δ 165.0, 142.7, 134.7, 130.6, 127.1, 68.1, 59.7, 48.7, 38.7, 30.3, 29.7, 28.8, 23.8, 22.8, 13.9, 10.9.

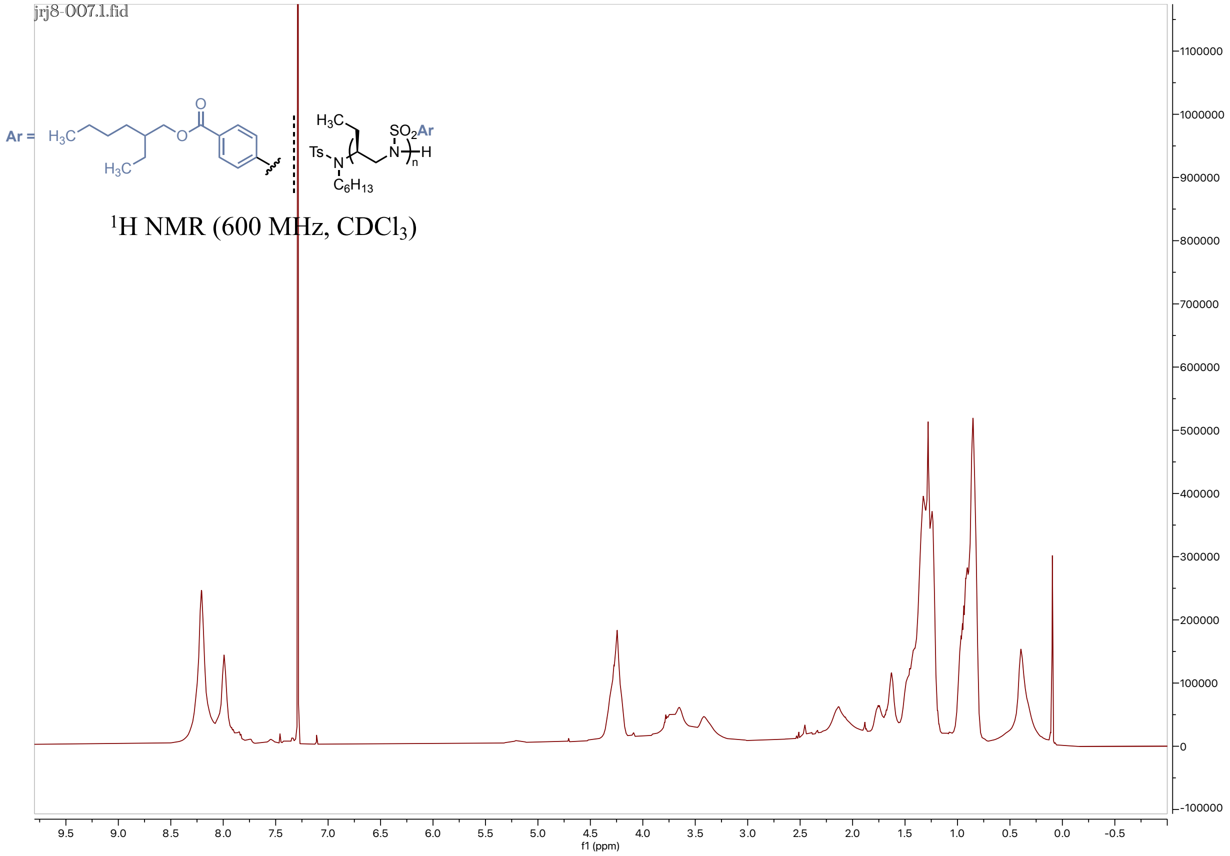

Figure S30. ^{1}H NMR spectrum of poly(**9**) in $CDCl_3$.

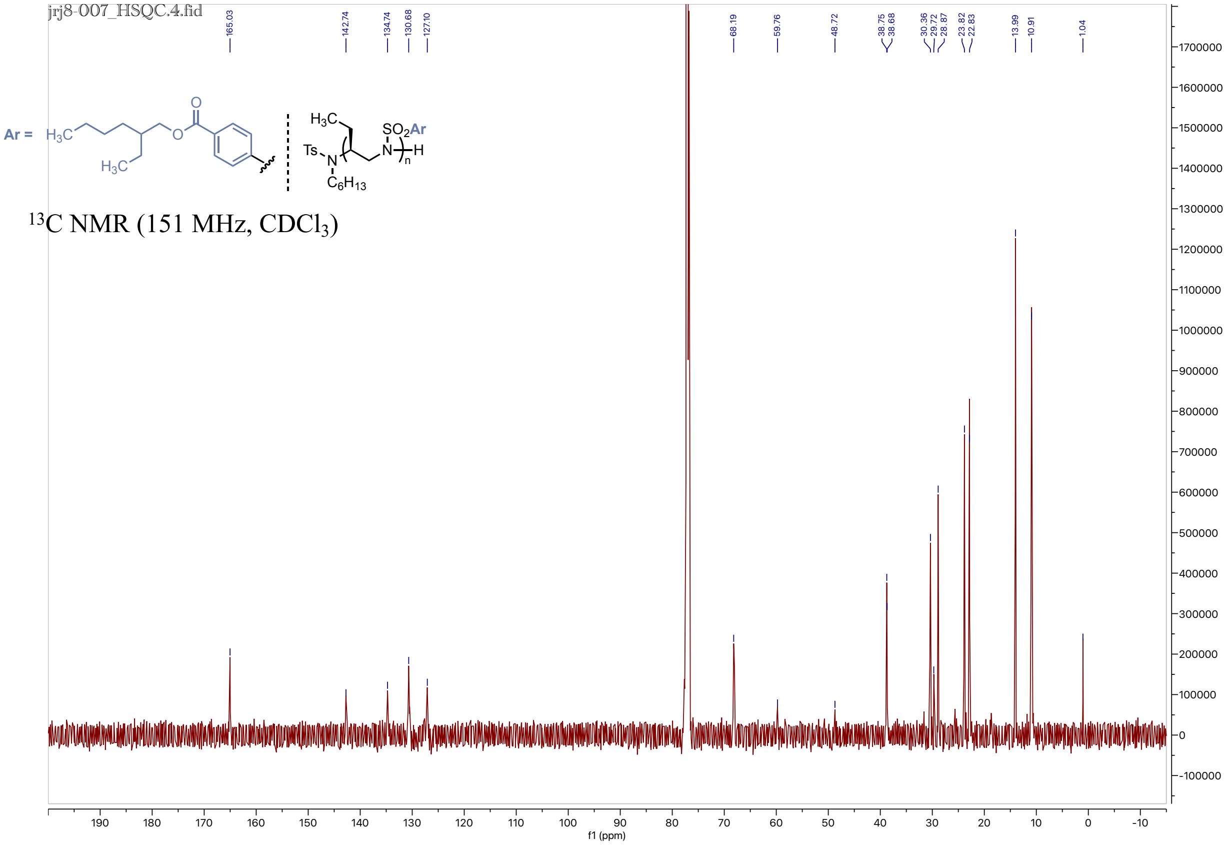

Figure S31. ^{13}C NMR spectrum of poly(**9**) in $CDCl_3$.

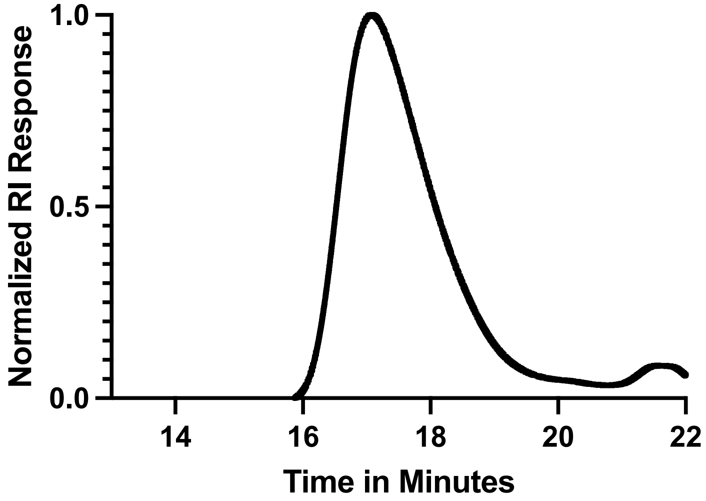

Figure S32. SEC traces of poly(**9**) in THF.

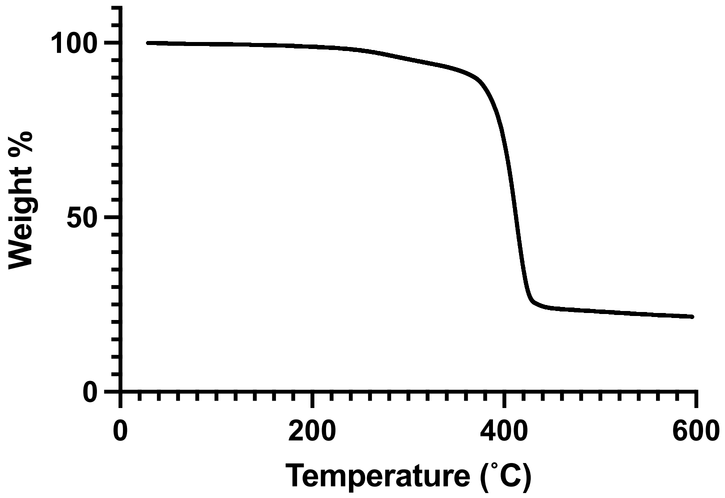

Figure S33. TGA of atactic poly(**9**) using a ramp rate of 20˚C/min.

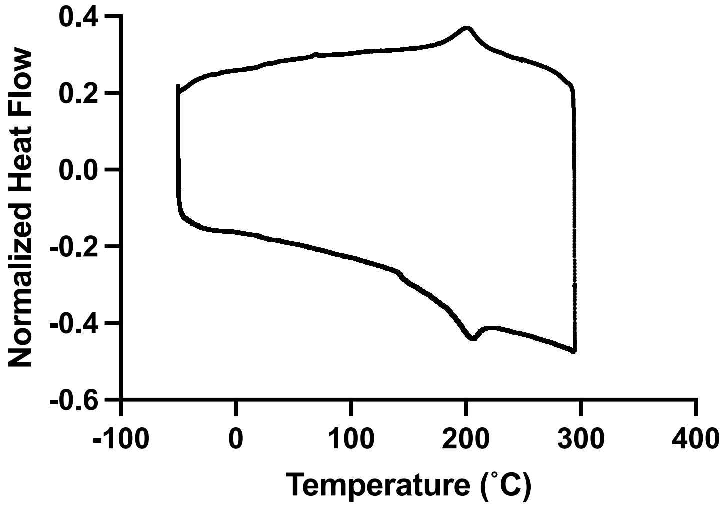

Figure S34. DSC of poly(**9**) using a ramp rate of 5˚C/min after removal of thermal history. T_g = 125˚C; T_m = 200˚C

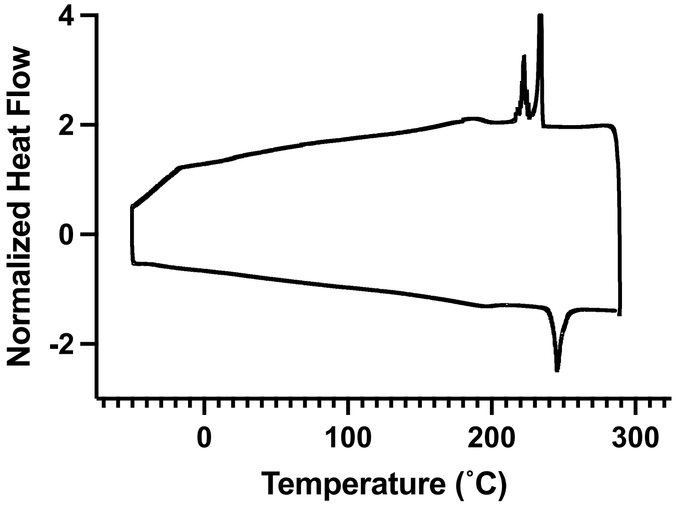

Figure S35. DSC of a 1:1 blend of poly(**9**) using a ramp rate of 10˚C/min after removal of thermal history. T_g = 145˚C; T_m = 242˚C

5 MALDI-TOF MS of poly(3)

Matrix : DCTB (20 mg/mL THF)
Analyte: poly(**3**) (5 mg/mL THF)
Cation dopant: NaI (1 mg/mL THF)
Sample prep: 20 μL matrix + 5 μL analyte + 1μL cation dopant and mixed thoroughly
Applied 1 μL of solution to the MALDI-TOF MS plate using the dried droplet method

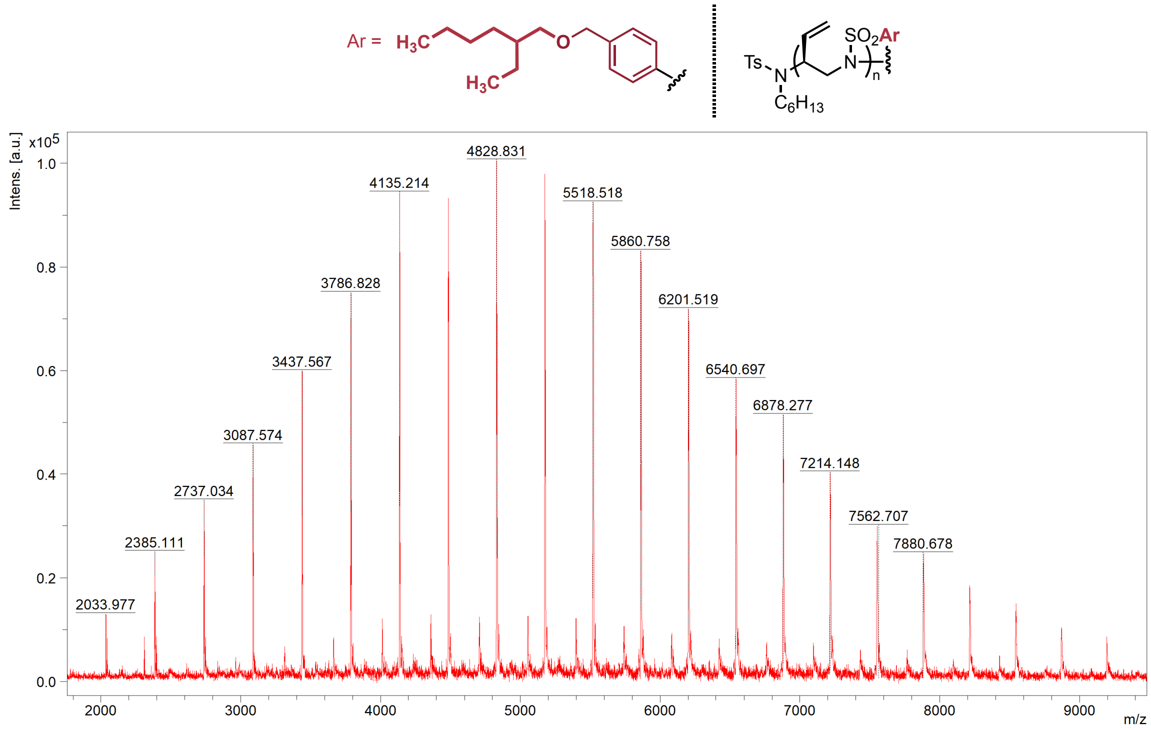

Figure S36. MALDI-TOF MS of poly(**3**).

Exact Mass exp: 2033.977
Exact Mass theo (Na + **monomer(5.0)** + **initiator**) = 2034.054
Δ = 0.07 Da

Exact Mass exp: 2385.111
Exact Mass theo (Na + **monomer(6.0)** + **initiator**) = 2385.241
Δ = 0.13 Da

Exact Mass exp: 3087.574
Exact Mass theo (Na + **monomer(8.0)** + **initiator**) = 3087.615
Δ = 0.04 Da

6 Characterization of tacticity and enantioselectivity of polymers

6.1 Tacticity

All tacticity calculations were conducted using ^{13}C NMR (8192 scans) with a 1-sec relaxation delay. All spectra *were baseline corrected with splines correction* prior to analysis. No smoothing functions were used as these can introduce additional error in the measurement.

Peak assignment for carbons can be seen in Figure S4 and matches with previous reports of aziridine polymerization.[7,8] No formation of the linear regioisomer is observed by ^{1}H or ^{13}C NMR.[9]

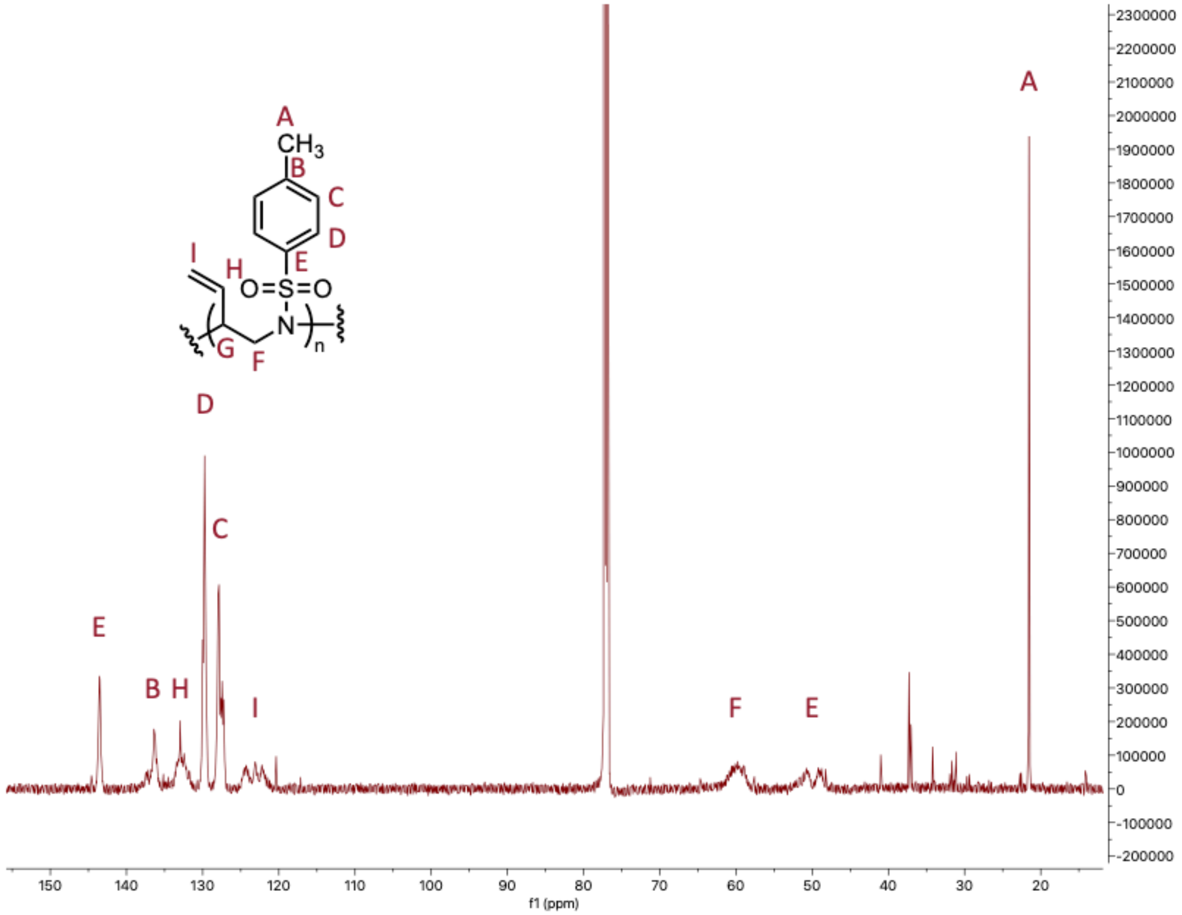

Figure S37. Peak assignments for the carbons of poly(**2**) in $CDCl_3$.

Identification of a suitable resonance for determination of tacticity was accomplished using ^{1}H-^{13}C HSQC experiments. Based on the ^{1}H-^{13}C HSQC of atactic poly(**2**) (Figure S5), we identified the two resonances between 48-51 ppm to be promising as they correspond to the diastereotopic methylene protons of the repeat unit.

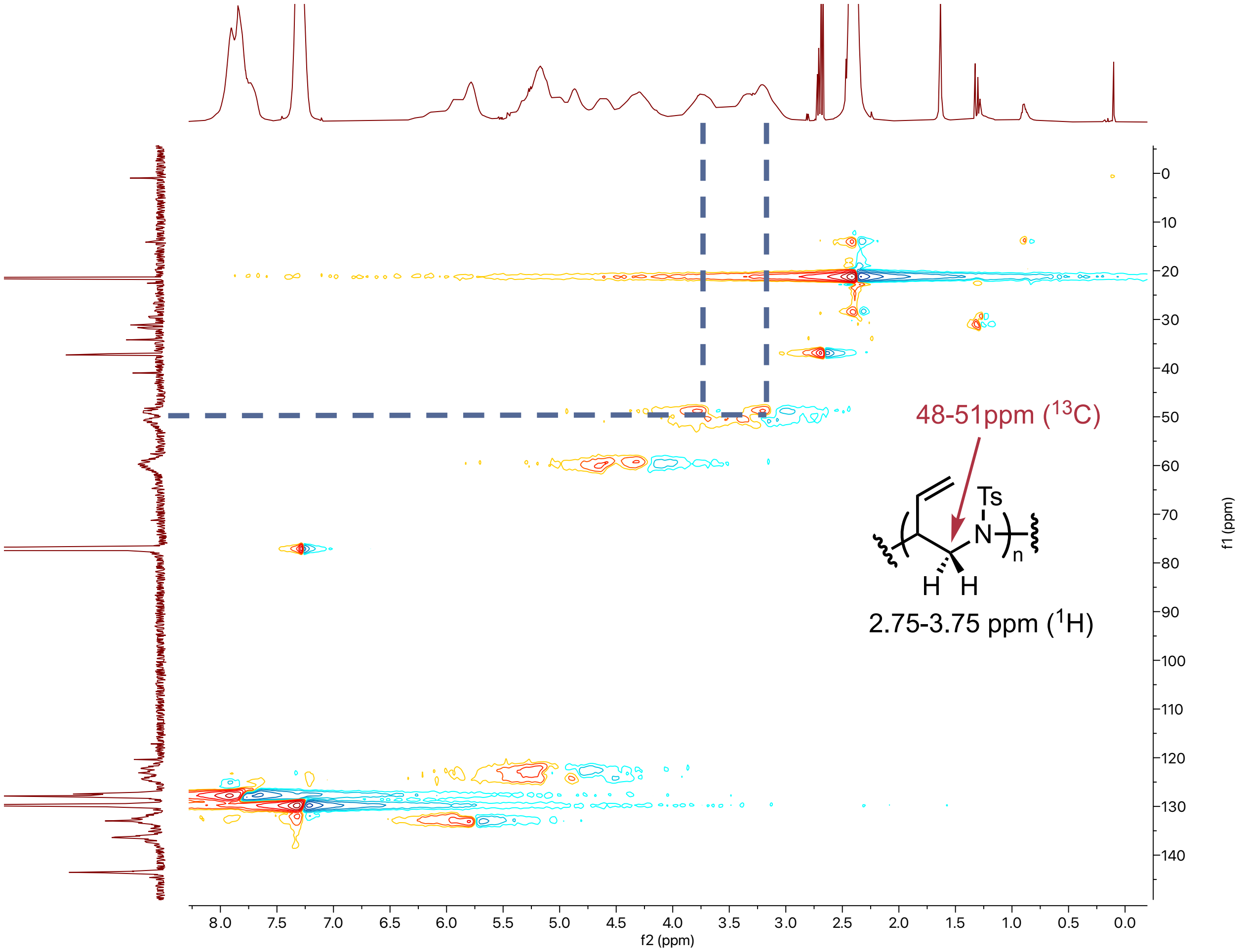

Figure S38. HSQC of poly(**2**) in $CDCl_3$.

Assignment of the meso and racemo diad was accomplished by polymerizing a 5:1 ratio of *R*-**2**:*rac*-**2** using anionic polymerization with KHMDS and the isolated polymer was analyzed using ^{13}C NMR spectroscopy and compared to atactic poly(**2**) (Figure S6).

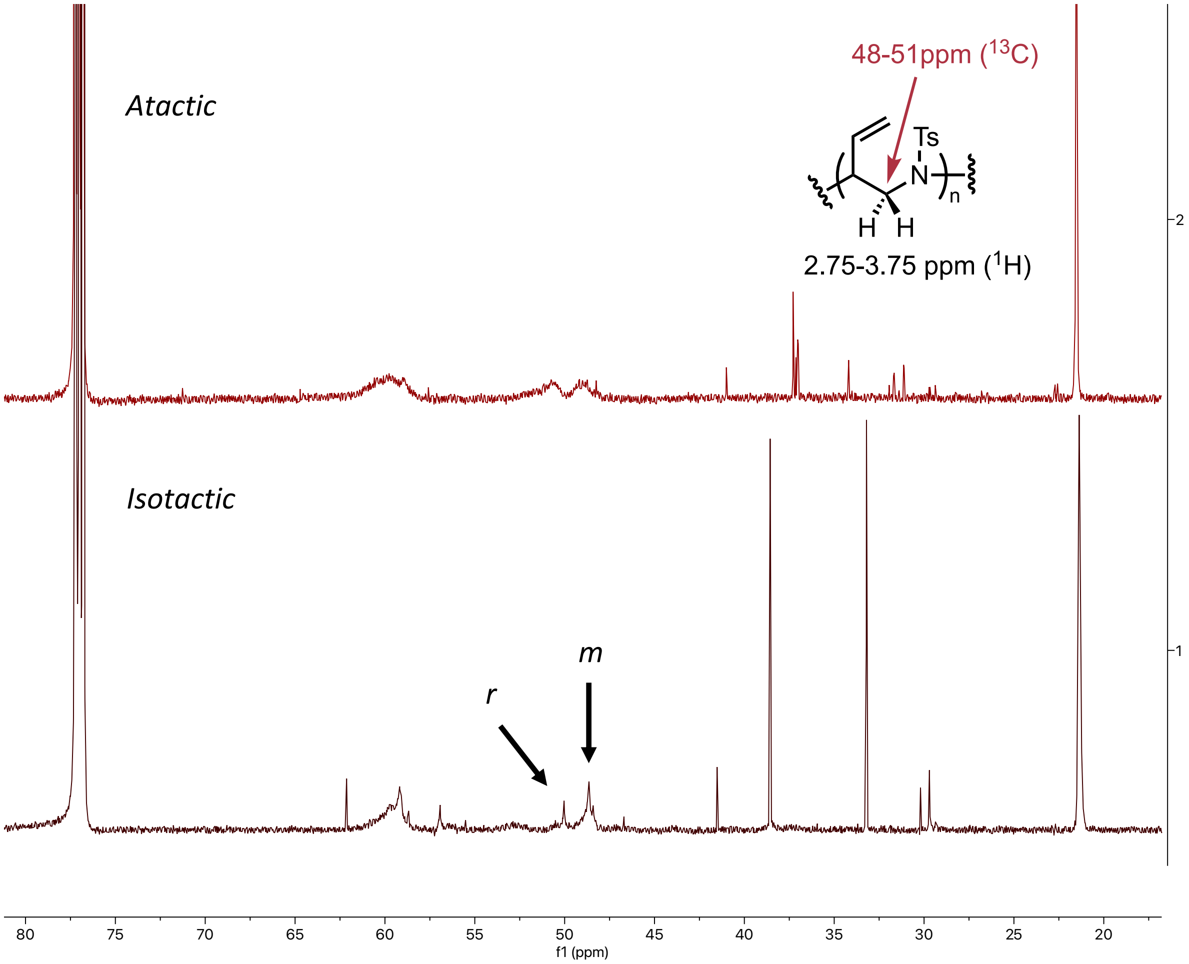

Figure S39. ^{13}C NMR of poly(**2**) in $CDCl_3$. Top = atactic, bottom = isotactic enriched.

Based on these data, 49-50 ppm was determined to be the *meso* diad and 50-51 ppm to be the *racemo* diad. These were applied to poly(**3**) by analogy.

6.2 Absolute Configuration of the Repeat Unit

The absolute configuration of the repeat unit was determined by comparing CD spectra of poly(**3**)s synthesized with *rac*-**3** with BINAP and enantioenriched *S*-**3** (95% ee by HPLC) polymerized with P_4-phosphazene. Based on the data, *S* stereocenters are set in poly(**3**) when using *R*-BINAP (Figure S8).

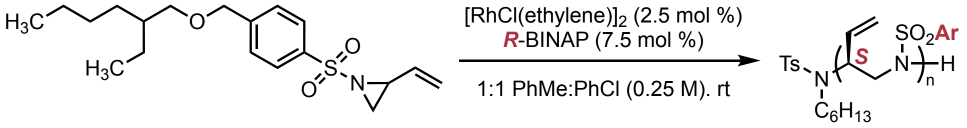

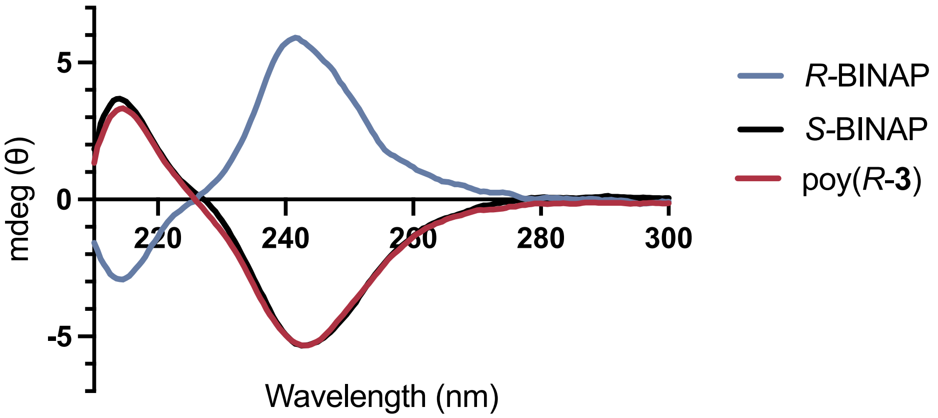

Figure S40. CD spectra of poly(**3**)s to determine absolute configuration of the repeat unit in the polymer.

7 Circular Dichroism and Optical Rotation of polymers

7.1 Optical Rotation Data of poly(3)

Table S7. Optical rotation data for poly(**3**)

Polymerization Conditions								
Ligand	Base	Solvent	M_n	% m	α	*l* (dm)	*c* (g/mL)	[α]
R-Binap	LiHMDS	THF	5.8	99	0.0122	1	0.00025	48.8
R-Binap	LiHMDS	PhCl	4.3	99	0.0302	1	0.00025	120.8
R-Binap	LiHMDS	PhMe	3	99	0.02	1	0.00025	80
R-Binap	LiHMDS	1:1 PhCl:PhMe	4.3	99	0.0253	1	0.00025	101.2
R-Binap	LiHMDS	1:1 PhCl:PhMe	4.8	99	0.0234	1	0.00025	79.6
R-Binap	P4	1:1 PhCl:PhMe	5.9	99	0.0199	1	0.00025	93.6
S-Binap	P4	1:1 PhCl:PhMe	9.6	99	-0.0254	1	0.00025	-101.6

7.2 Circular Dichroism

All CD was conducted at 0.1 mg/mL in THF (no inhibitor) at 23˚C unless specified below.

Circular dichroism and absorbance data for the *L*-monomer **3** and polymer synthesized using conditions outlined in 2.1 with *R*-Binap (*vide infra*). Data shows clear differences in absorbances for **3** compared to poly(**3**), notably the λ_{max} has shifted from 227 nm to 236 nm.

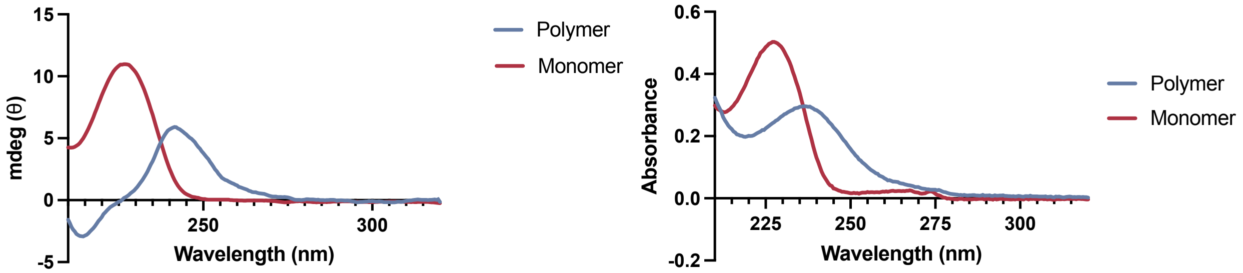

Figure S41. CD and absorbance data for monomer **3** and poly(**3**).

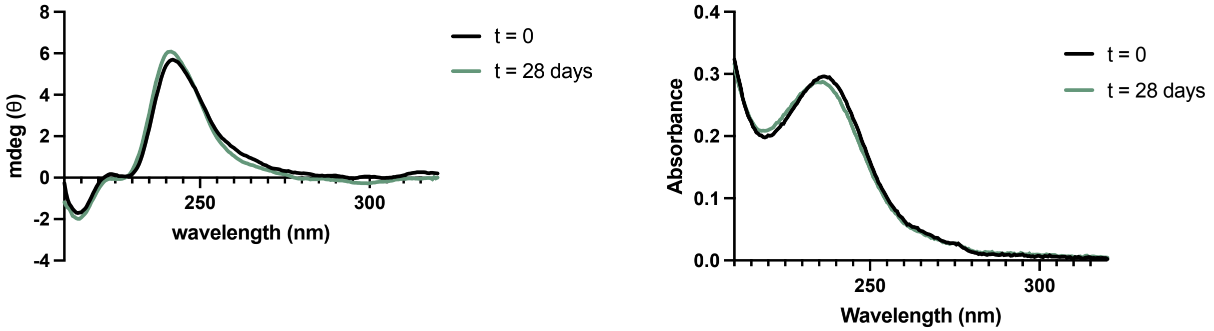

Figure S42. Time course study on CD response over time.

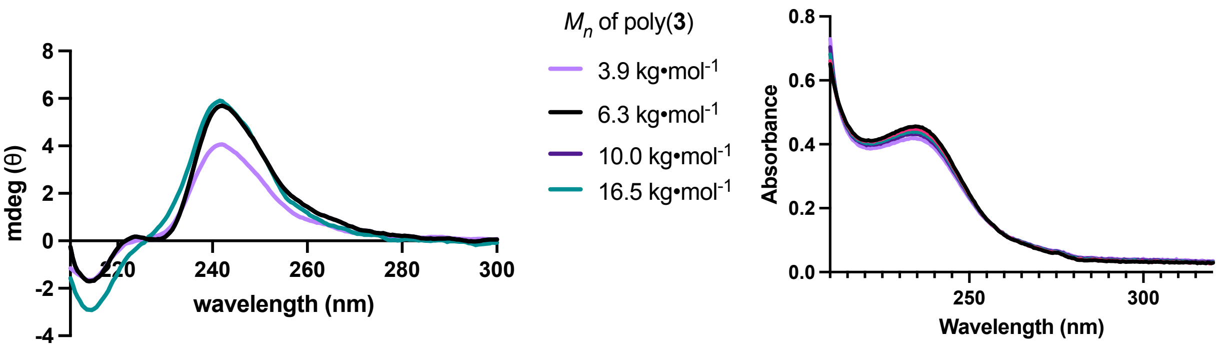

Figure S43. CD of poly(**3**) with varying target molar mass. No change in CD response is observed albeit the 3.5k target poly(**3**).

To investigate the stability of isotactic poly(**3**) helices to inversion a sample prepared with a M_n of 9.0 kDa and 99% m using was dissolved in HPLC grade THF at 1 mg/mL, capped, and left on the bench top where it was exposed to ambient temperature and sunlight for 4 weeks. Shown below are the CD traces of the sample on day 1 and after 4 weeks, where little to no change in CD response is seen—indicating samples are solution stable for up to 4 weeks.

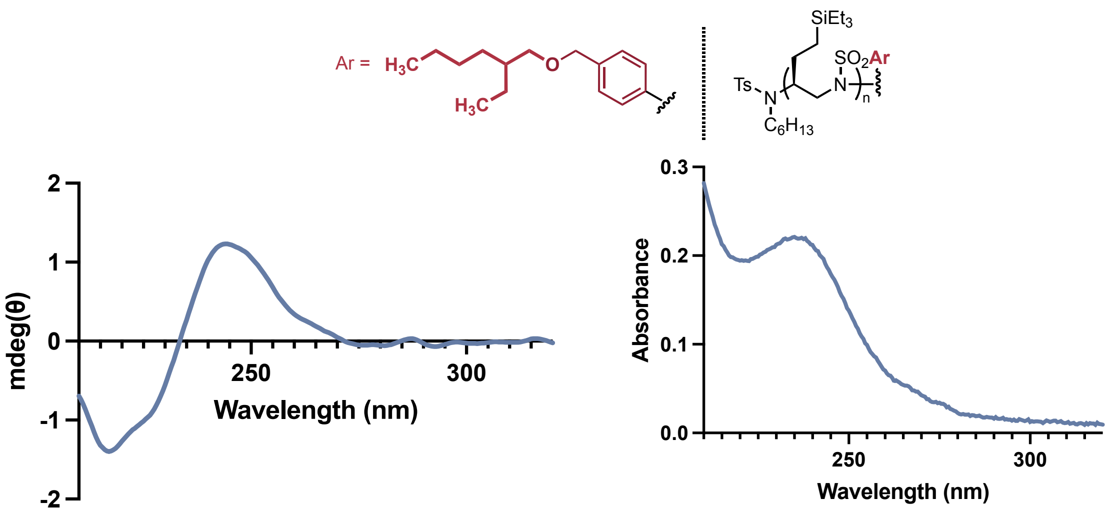

Figure S44. CD and UV-Vis spectrum of poly(**6**) in THF at 0.1 mg/mL.

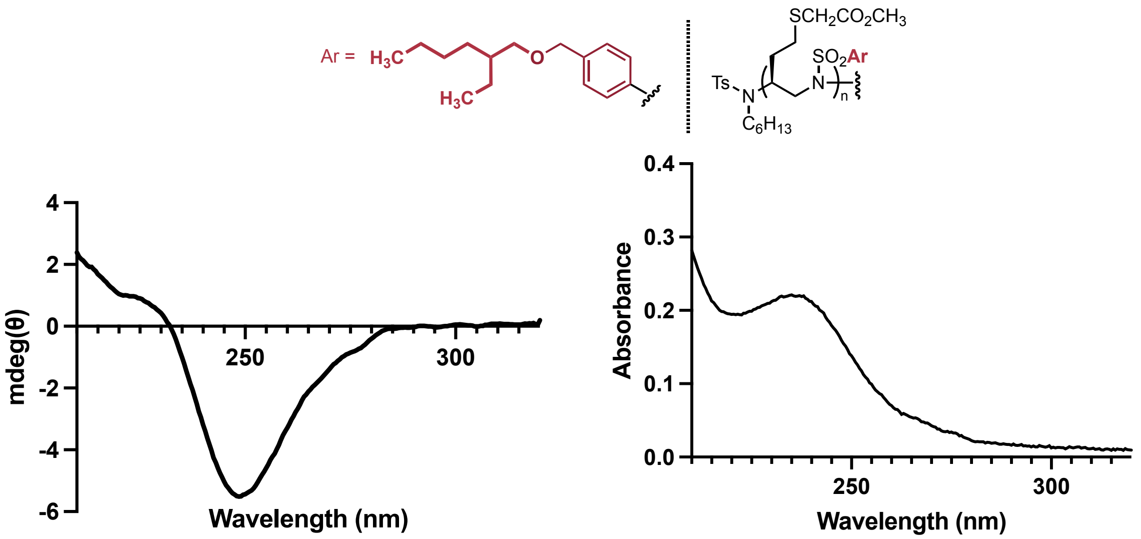

Figure S45. CD and UV-Vis spectrum of poly(**7**) in THF at 0.1 mg/mL.

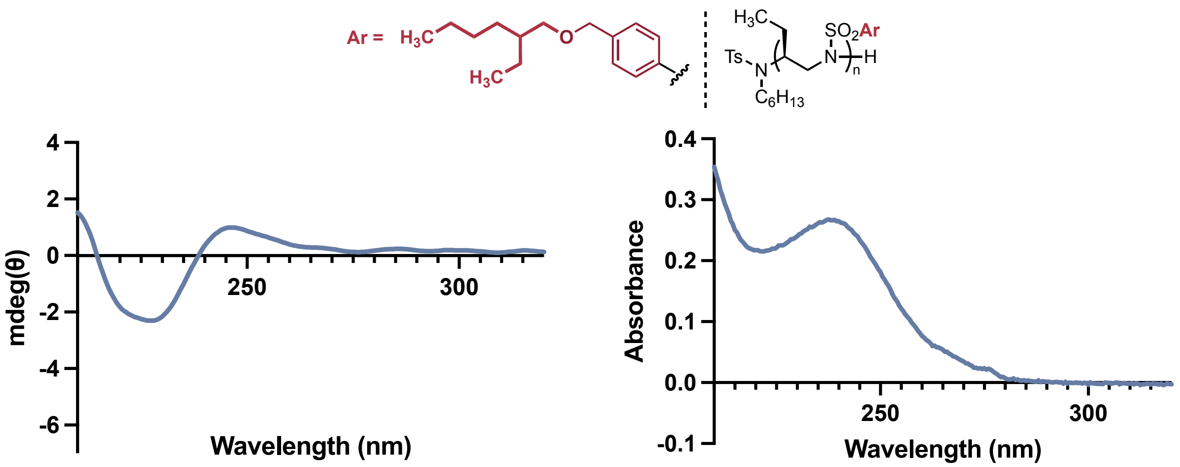

Figure S46. CD and UV-Vis spectrum of poly(**8**) in THF at 0.1 mg/mL.

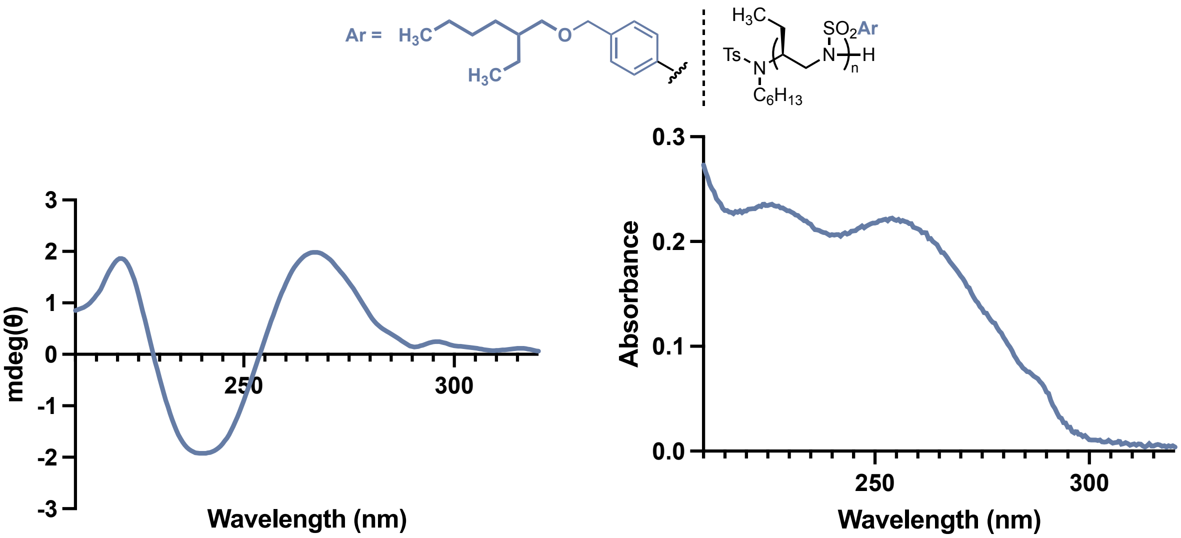

Figure S47. CD and UV-Vis spectrum of poly(**9**) in THF at 0.1 mg/mL.

8 NMR Studies of model π-allyl complex

8.1 Synthesis

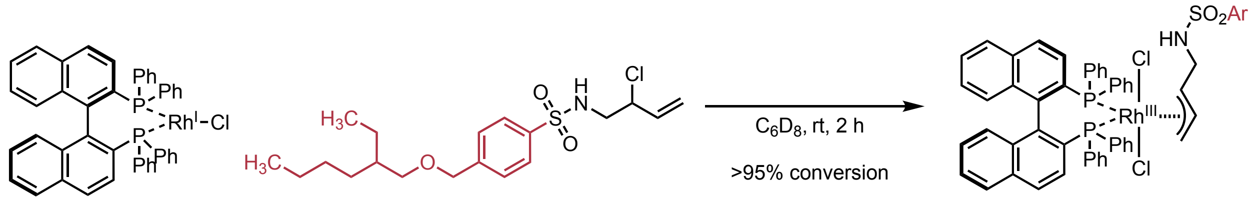

Complex I

In an N_2-filled glovebox, an 8-mL vial was charged with $[Rh(C_2H_4)_2Cl]_2$ (15.0 mg, 39 μmol, 1.0 equiv), *R*-BINAP (49.0 mg, 78 μmol, 2.0 equiv), followed by C_7D_8 (0.5 mL), and stirred for 15 min to ensure full complexation. To the solution was added *N*-(2-chlorobut-3-en-1-yl)-4-(((2-ethylhexyl)oxy)methyl) benzenesulfonamide **S1** (30.2 mg, 78 μmol, 2.0 equiv) in C_7D_8 (0.1 mL), and the reaction was monitored by ^{31}P NMR until all catalyst was consumed and **5** was formed. Once complete, the contents were transferred to an oven-dried J-Young tube for analysis by NMR.

The metal complex was stable for up 7 days in a J-Young tube at 23˚C

^{1}H NMR (600 MHz, C_7D_8) δ 10.43 – 10.21 (m, 1H), 8.95 – 8.66 (m, 2H), 8.59 – 6.24 (m, 39H), 6.10 (t, *J* = 7.5 Hz, 1H), 5.92 (dd, *J* = 27.9, 9.8 Hz, 1H), 5.65 (d, *J* = 42.6 Hz, 2H), 5.16 (bs, 1H), 4.65 (bs, 1H), 4.45 (bs, 1H), 4.31 – 4.04 (m, 2H), 3.63 (bs, 2H), 3.42 – 3.27 (m, 1H), 3.08 (d, *J* = 49.9 Hz, 2H), 2.01 (d, *J* = 17.9 Hz, 2H), 1.66 – 1.18 (m, 8H), 0.91 (dt, *J* = 26.9, 7.1 Hz, 6H).

^{13}C NMR (151 MHz, C_7D_8) δ 170.6, 154.3, 143.5 (d, *J* = 33 Hz), 141.9, 141.2 (d J = 53 Hz), 140.0, 135.9 (d, J = 36 Hz), 135.1, 134.6 (d, J = 26 Hz), 134.3, 134.0, 133.7, 133.5, 133.4, 133.0, 132.6, 131.6, 131.2, 130.7, 130.4, 130.1, 129.7, 129.4, 127.4, 127.0, 126.7, 126.5, 126.0, 125.8, 122.8, 117.8 (d, J = 23 Hz), 113.7, 113.5, 112.1, 107.7, 73.3, 72.3, 49.7 (d, J = 8 Hz), 47.0, 45.3, 44.9 (d, J = 11 Hz), 40.2, 31.0, 29.5, 24.3, 23.5, 14.4, 11.4.

Overlapping peaks with C_7D_8 limit full characterization of the aryl region

^{31}P NMR (202 MHz, Toluene) δ 27.57 (dd, *J* = 161.7, 22.4 Hz), 27.98 (dd, *J* = 113.2, 17.4 Hz), 30.44 (dd, *J* = 152.4, 16.8 Hz), 30.76 (dd, *J* = 114.4, 22.4 Hz)

8.2 Comparison of 5 to Rh-BINAP + 3

The complex derived from the catalyst and aziridine **3** could not be observed due to rapid oligomerization through an initiator-free pathway.[9,10]

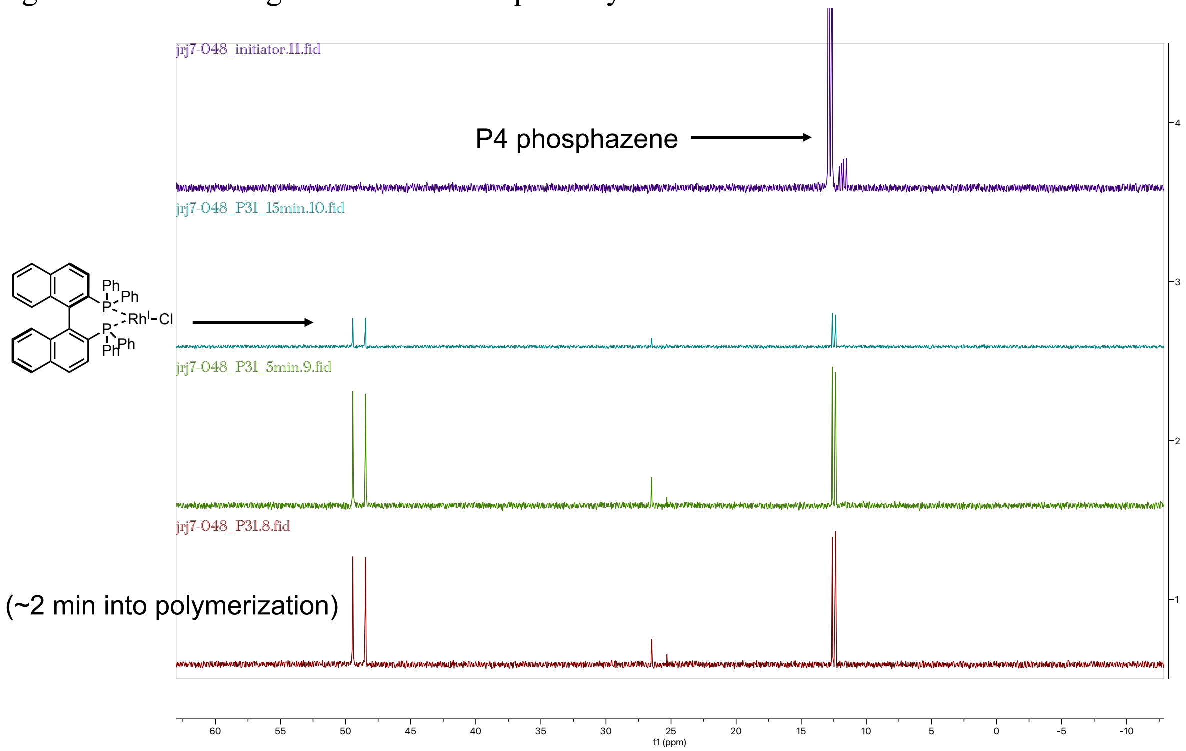

Figure S48. ^{31}P NMR of polymerization in NMR tube.

We sought to tame the reactivity via addition with an additional ligand (chloride), as well as protonation of the sulfonamide (ie compound **4**). This has been shown to lead to stable π-allyl complexes for spectroscopic studies. While the addition does alter the structure of the p-allyl complex, the bonding (η^1 vs σ+π enyl coordination), presence of multiple diastereomers (Pro-R and Pro-S), and dynamic behavior (π–σ–π isomerization) would provide supporting evidence for stereoconvergence.

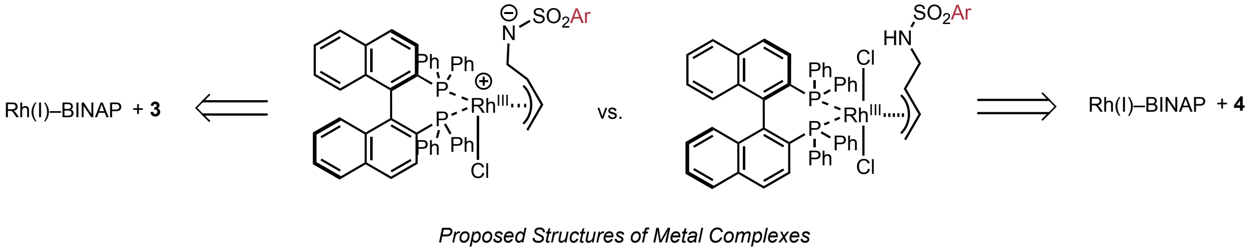

Figure S49. Comparison of complexes derived from Rh-BINAP + **3** vs. Rh-BINAP + **4**.

8.3 Observation of two diastereomers with "enyl" η^3 binding

Based on previous studies of rhodium π-allyl complexes, there exists two possible modes of binding to the allyl complex, η^1 and η^3 (Figure S16). Based on the number of ^{31}P signals and the presence of P-P coupling, the data supports η^3 binding.[11,12] From these data, we surmised there are two η^3 diastereomers in solution as there are no Rh-P couplings larger than 400 Hz known in the literature. Peaks for diastereomers were identified based on identical P–P coupling constants.

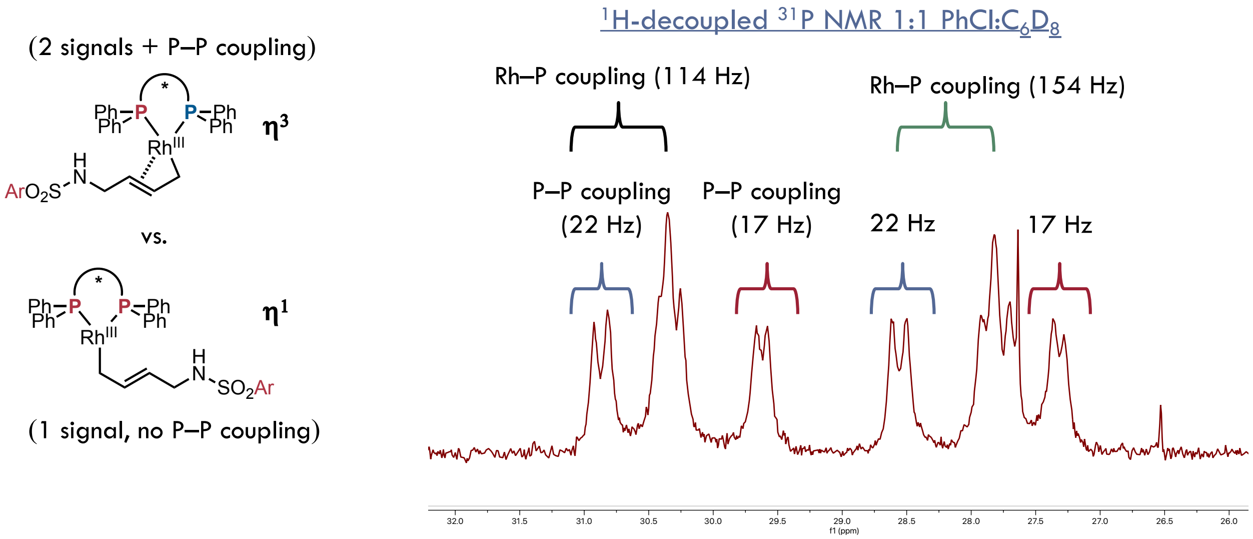

Figure S50. Rationale for η^3 binding to the π-allyl complex in **5** and the observation of multiple diastereomers.

Based on ^{1}H, ^{13}C and ^{31}P NMR spectra, we propose the two diastereomers of **5** are pro-R and Pro-S (Figure S17). However, the from these data we cannot identify which complex is Pro-R. At 23°C there exists a 1:1 relationship between Pro-R and Pro-S forms.

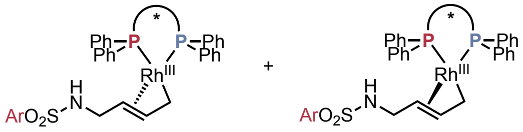

Pro-*S* **Pro-*R***

Figure S51. Proposed structures of η^3 π-allyl complexes in solution.

Enyl-type bonding was observed for both diastereomers based on variation of ^{1}H and ^{13}C chemical shifts and splitting of the allyl moiety. For Complex I, H_A and $H_{A'}$ (Figure S18A, green) are found between 2-3 ppm compared to H_B and H_C which are found closer to the alkene region of the ^{1}H NMR spectrum (4-5 ppm, Figure S18A, blue). Similar observations are observed with the chemical shifts of carbon A (40-50 ppm, Figure S18B) compared to carbons B and C (~110 ppm, Figure SXB) Furthermore, the carbon bonded to H_A and $H_{A'}$ exhibits Rh-C splitting of 7 and 11 Hz for the Pro-R and Pro-S forms, suggesting a formal covalent bond to the rhodium (Figure S18B). No Rh-C splitting is observed for the carbons bonded to H_B and H_C. However, broadening of H_B and H_C's ^{13}C resonance supports a dynamic process in solution.

Enyl bonding indicates there is little contribution of the resonance form wherein the metal sits on the other carbon atom (Figure S18C). This ultimately leads to a regiochemical memory effect as oxidative

addition and propagation occur via S_N2' mechanisms which leads to net retention of regiochemistry in the polymerization and has been seen previously.[13,14]

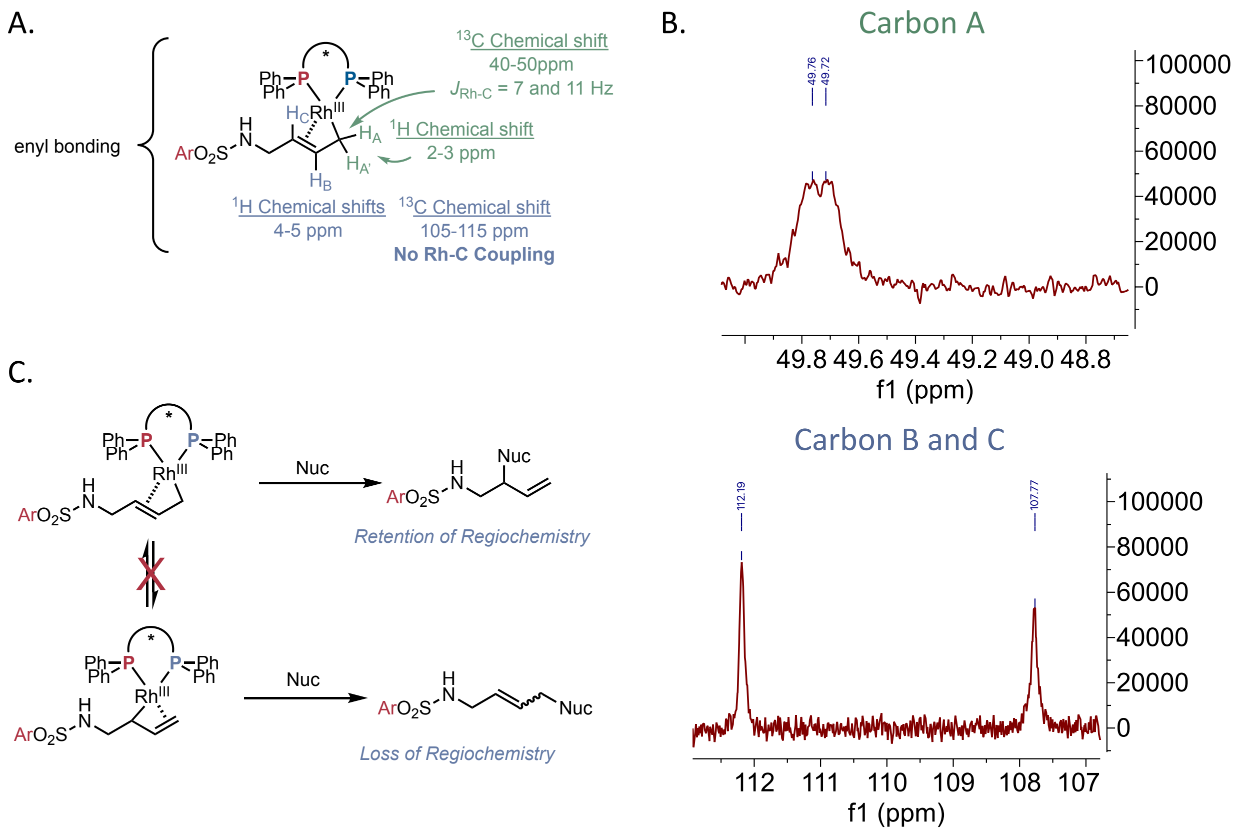

Figure S52. A. Observed chemical shifts and Rh-C J couplings supporting enyl bonding; B. Observed splitting with Carbon A and lack of splitting with carbon B; C. Implications of enyl bonding on regiochemical outcomes.

8.4 VT-NMR studies on π–σ–π isomerization of 5

5 was monitored by {H}^{31}P NMR as temperature decreased in 1:1 PhCl:C_7D_8 at concentrations relevant to catalysis (1.8 mg Rh per 0.75 mL solvent). Spectra were collected with 64 scans and a 5 second relaxation delay. At 23˚C, broadening of resonances is observed indicative of a dynamic process in solution and there is a 1:1 ratio of diastereomers (Pro-R and Pro-S) (Figure S19). Upon cooling to 10˚C, resonances notably sharpen, indicating a decrease in dynamic behavior. Slight broadening at -30˚C was observed but was attributed to increased viscosity of the solution considering the freezing point of chlorobenzene is -45˚C. We hypothesize the dynamic process occurring in solution is π–σ–π isomerization based on previously observed dynamic behavior of π-allyl complexes.[15]

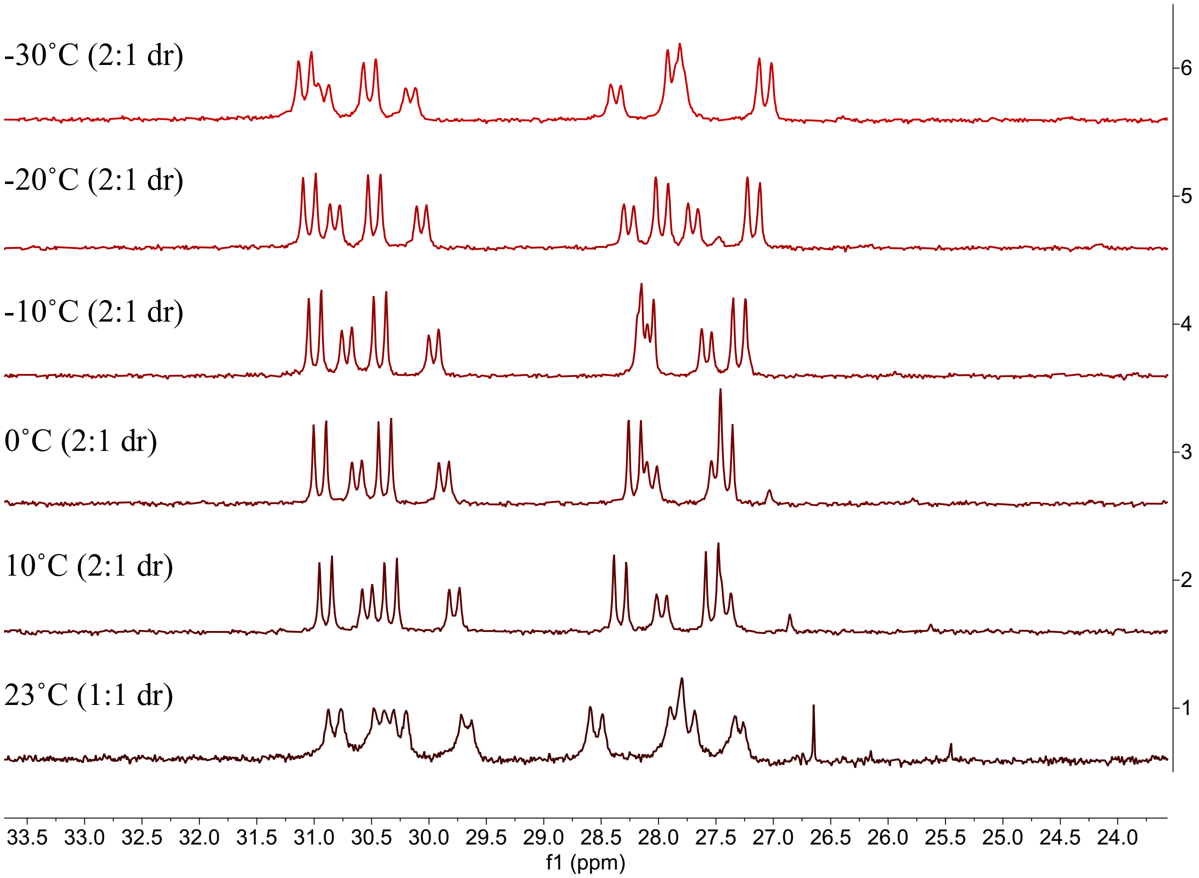

Figure S53. Stacked {H}^{31}P NMR spectra of **5** in 1:1 PhCl:C_7D_8 at various temperatures. Data were collected with 64 scans and a 5-second relaxation delay.

A recovery experiment was conducted wherein **5** was cooled from 23˚C to -30˚C, then allowed to warm back up to 23˚C whilst monitored by {H}^{31}P NMR Spectroscopy. After 5 min at 23˚C, another {H}^{31}P NMR spectrum was collected.

Upon cooling, a 1:1 distribution of diastereomers changes to 2:1, but recovers back to 1:1 after warming to 23˚C via π–σ–π isomerization (Figure S20). The data supports that the **Pro-R** and **Pro-S** diastereomers interconvert between each other readily at 23˚C but this is restricted at reduced temperatures in agreement with previous studies of π-allyl complexes. Upon cooling there is a slight preference for one diastereomer, which we hypothesize is due to interactions with the chiral ligand environment.

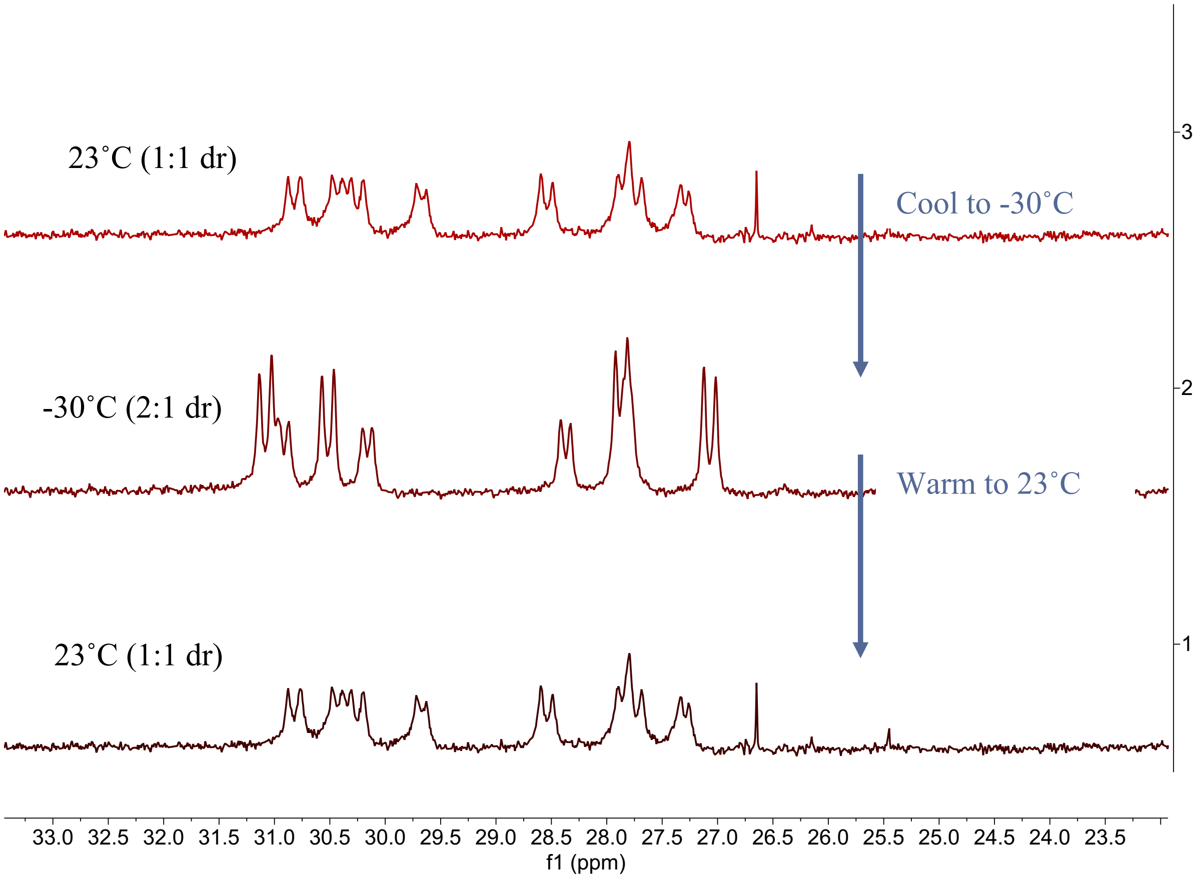

Figure S54. Stacked {H}^{31}P NMR spectra of **5** in 1:1 PhCl:C_7D_8 at various temperatures. Data were collected with 64 scans and a 5-second relaxation delay.

9 Mechanistic Analysis

9.1 Non-linear effect study

Polymerizations using varied enantiomeric excess of ligand were conducted following the general procedure for rhodium-catalyzed stereoconvergent polymerization of vinyl aziridines on 56 mg scale at 0.25 M. Two separate ligand solutions of R and S-BINAP were made and volumes were added to reach the overall target enantiomeric excess. Crude samples were analyzed using ^{1}H and ^{13}C NMR, SEC and the polymer was dissolved in 0.5 mL CH_2Cl_2 and precipitated into cold MeOH, and dried via vacuum to furnish pure polymer samples for analysis by optical rotation and CD.

The data supports a monomeric catalyst is active in the polymerization given the absence of non-linear effects (Figure S21).[16]

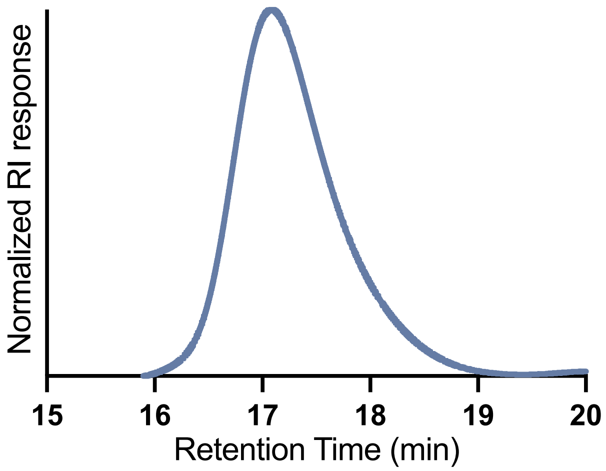

Figure S55. Representative SEC trace of poly(**3**) initiated with BINAP with varying %ee. M_n = 6.1 kg mol^{-1} and Đ = 1.30.

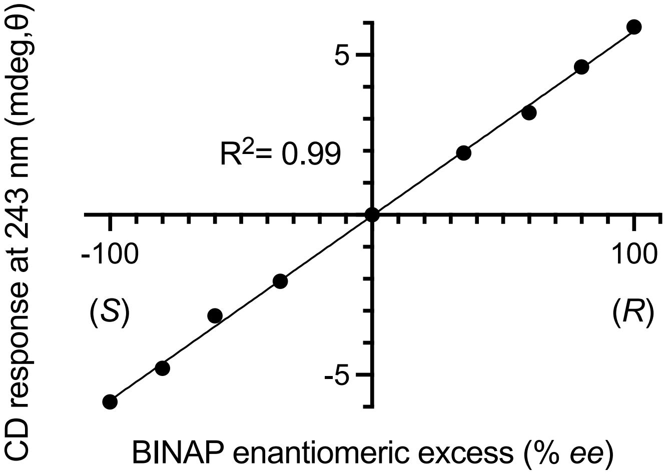

Figure S56. Ligand enantiomeric excess vs. CD response at 243 nm of poly(**3**) (0.1 mg/mL) synthesized using BINAP with varied %ee . Based on the data, there is no evidence of non-linear effects in this system.

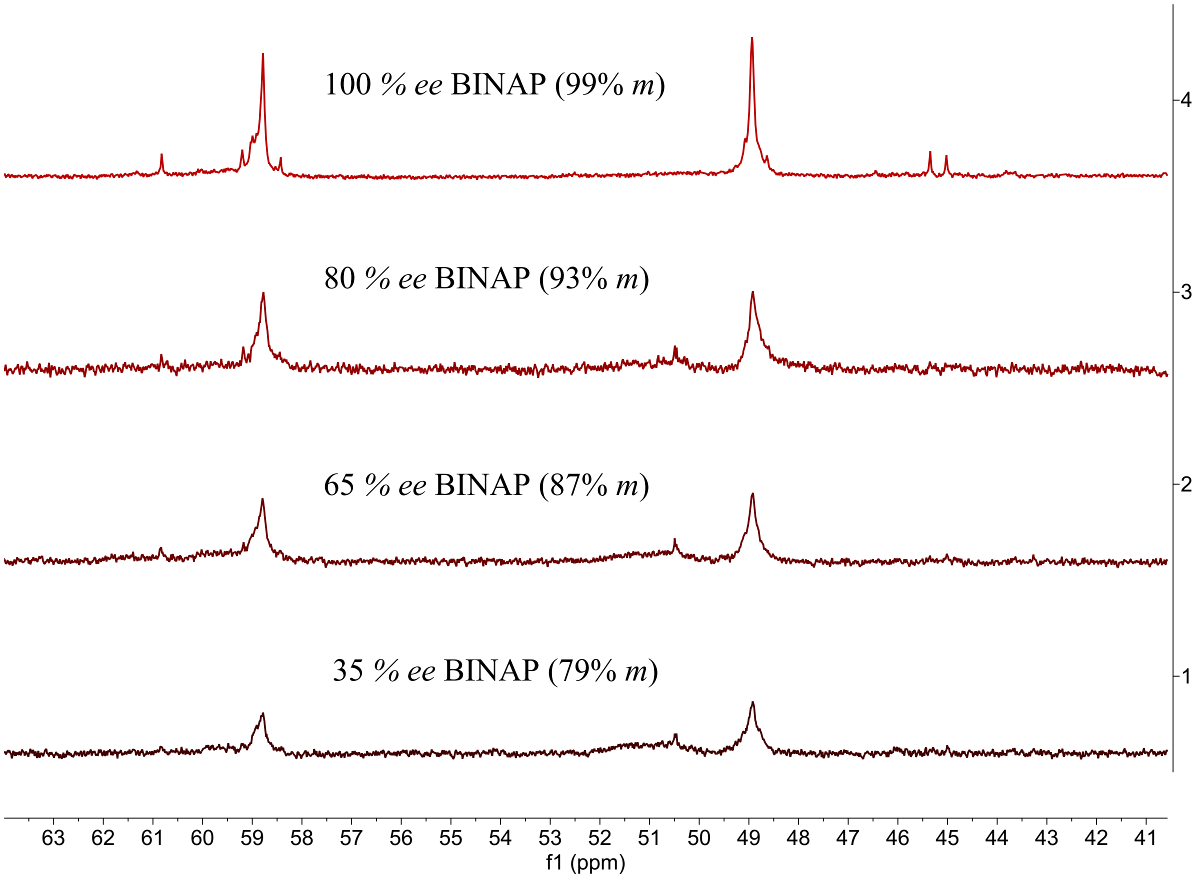

Figure S57. Tacticity calculations of poly(**3**)s based on *%ee* of BINAP using *rac*-**3**. All spectra were acquired with 8192 scans and corrected with splines prior to analysis.

9.2 Chiral initiator study

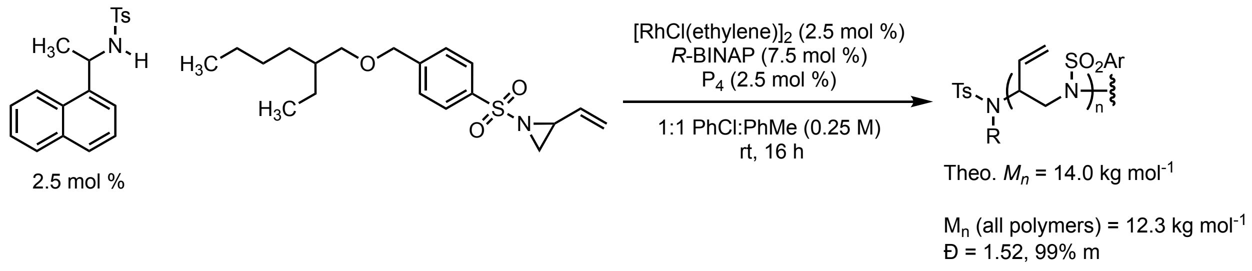

Polymerizations using 4-methyl-*N*-(1-(naphthalen-1-yl)ethyl)benzenesulfonamide of varying *% ee* were conducted following the general procedure for rhodium-catalyzed stereoconvergent polymerization of vinyl aziridines. Crude samples were analyzed using ^{1}H and ^{13}C NMR, SEC and the polymer was dissolved in 0.5 mL CH_2Cl_2 and precipitated into cold MeOH, and dried via vacuum to furnish pure polymer samples for analysis by ^{13}C NMR, optical rotation and CD.

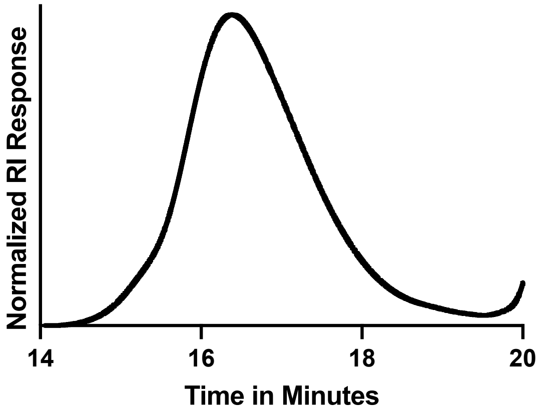

Figure S58. Representative SEC trace of poly(**3**) initiated with 4-methyl-*N*-(1-(naphthalen-1-yl)ethyl)benzenesulfonamide.

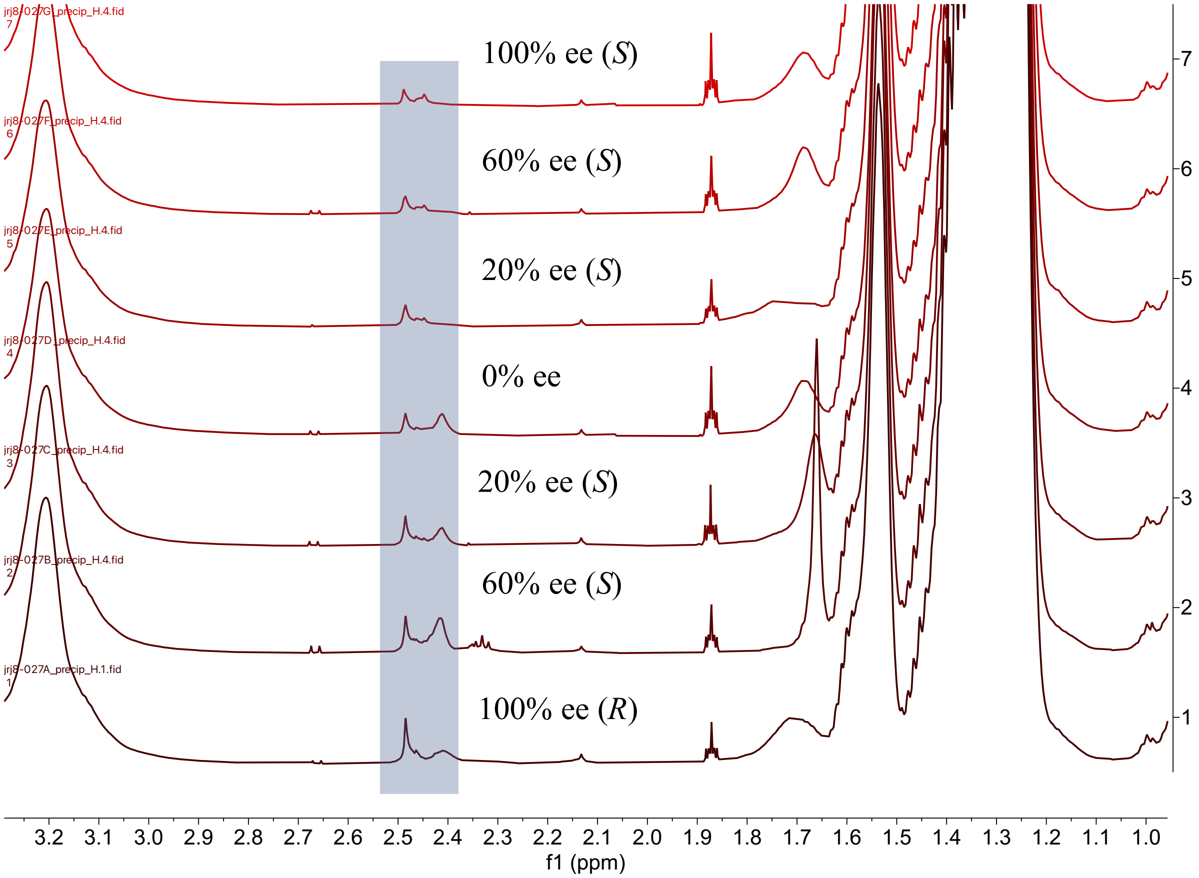

Figure S59. ^1H NMR in $CDCl_3$ of poly(**3**) initiated with 4-methyl-*N*-(1-(naphthalen-1-yl)ethyl)benzenesulfonamide showing the presence of the initiator.

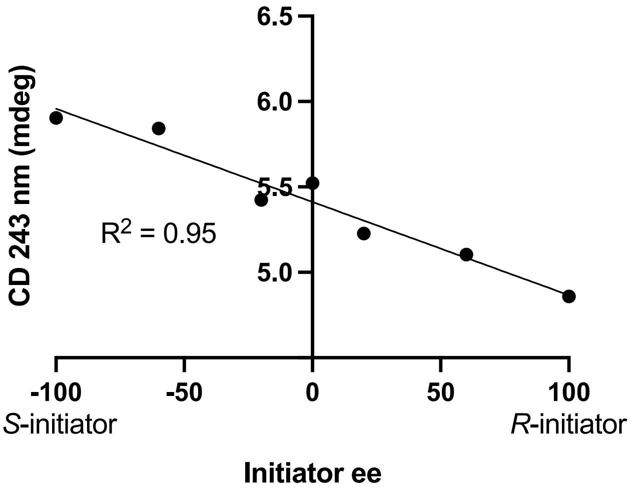

Figure S60. Plot of initiator *%ee* (4-methyl-*N*-(1-(naphthalen-1-yl)ethyl)benzenesulfonamide) vs. CD response of poly(**3**)s.

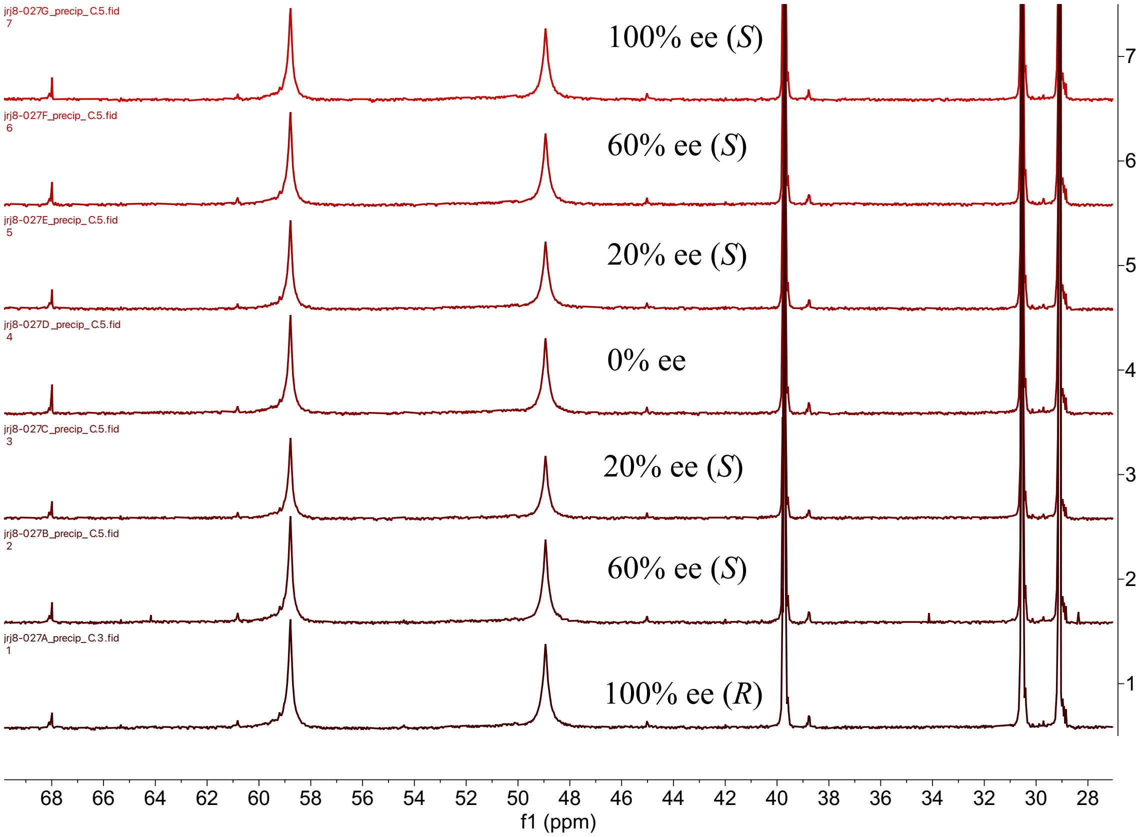

Figure S61. ^{13}C NMR in $CDCl_3$ of poly(**3**) initiated with 4-methyl-*N*-(1-(naphthalen-1-yl)ethyl)benzenesulfonamide. All samples exhibited 99% meso diads.

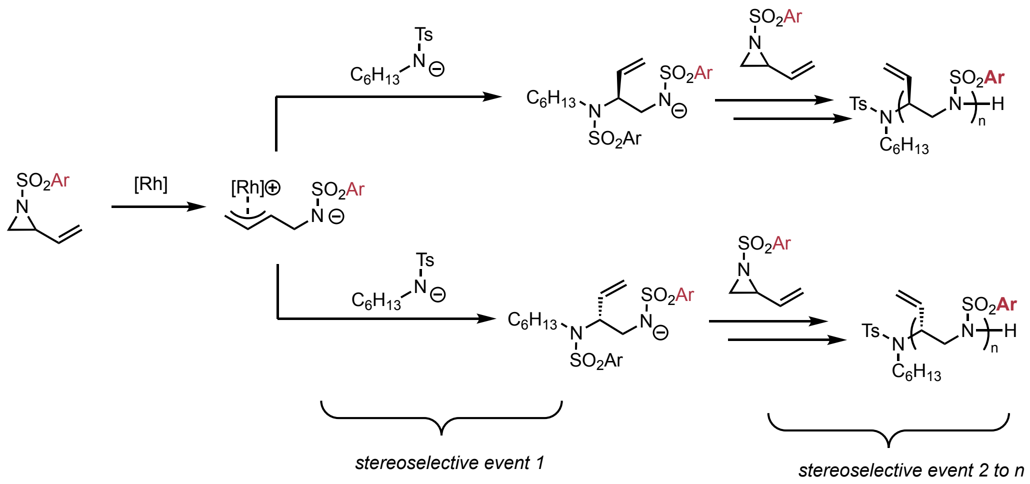

Figure S62. Stereoselective events occurring during polymerization.

Based on these data, the selectivity of the initiation strongly correlates with the overall enantioselectivity of the polymerization.[17] This is an agreement with the mechanism as the initiation sets the first chiral center

in the polymer (stereoselective event 1, Figure S25), and all proceeding propagations are diastereoselective (stereoselective event 2 to n, Figure S26).

9.3 Enantioenriched monomer experiments

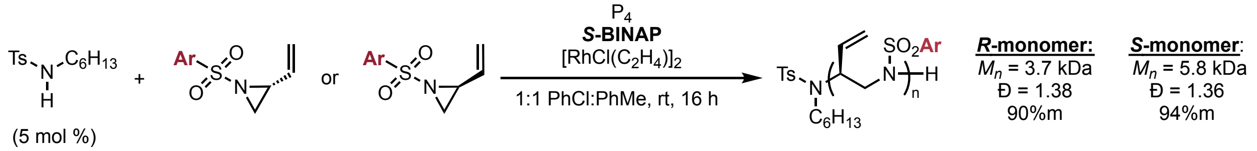

Polymerizations using enantioenriched **3** were conducted following the general procedure for rhodium-catalyzed stereoconvergent polymerization of vinyl aziridines on 30 mg scale and volumes used were scaled to match the same concentration (0.25 M). Crude samples were analyzed using ^{1}H and ^{13}C NMR, SEC and the polymer was dissolved in 0.5 mL CH_2Cl_2 and precipitated into cold MeOH, and dried via vacuum to furnish pure polymer samples for analysis by optical rotation and CD.

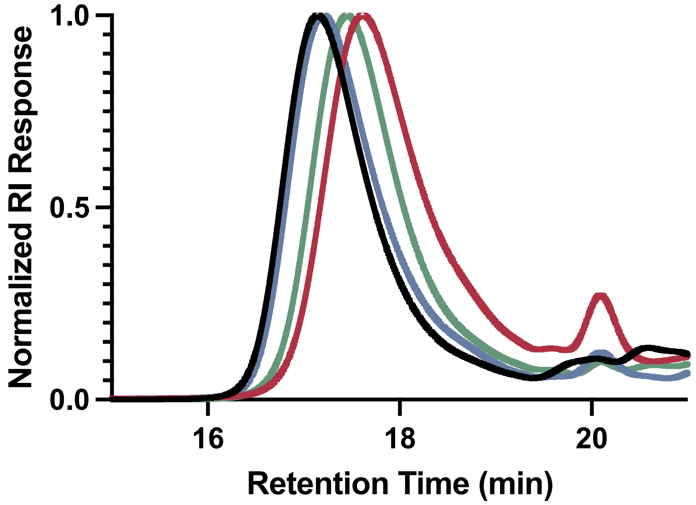

Figure S63. SEC in THF using an RI detector of poly(**3**) derived from enantioenriched **3**.

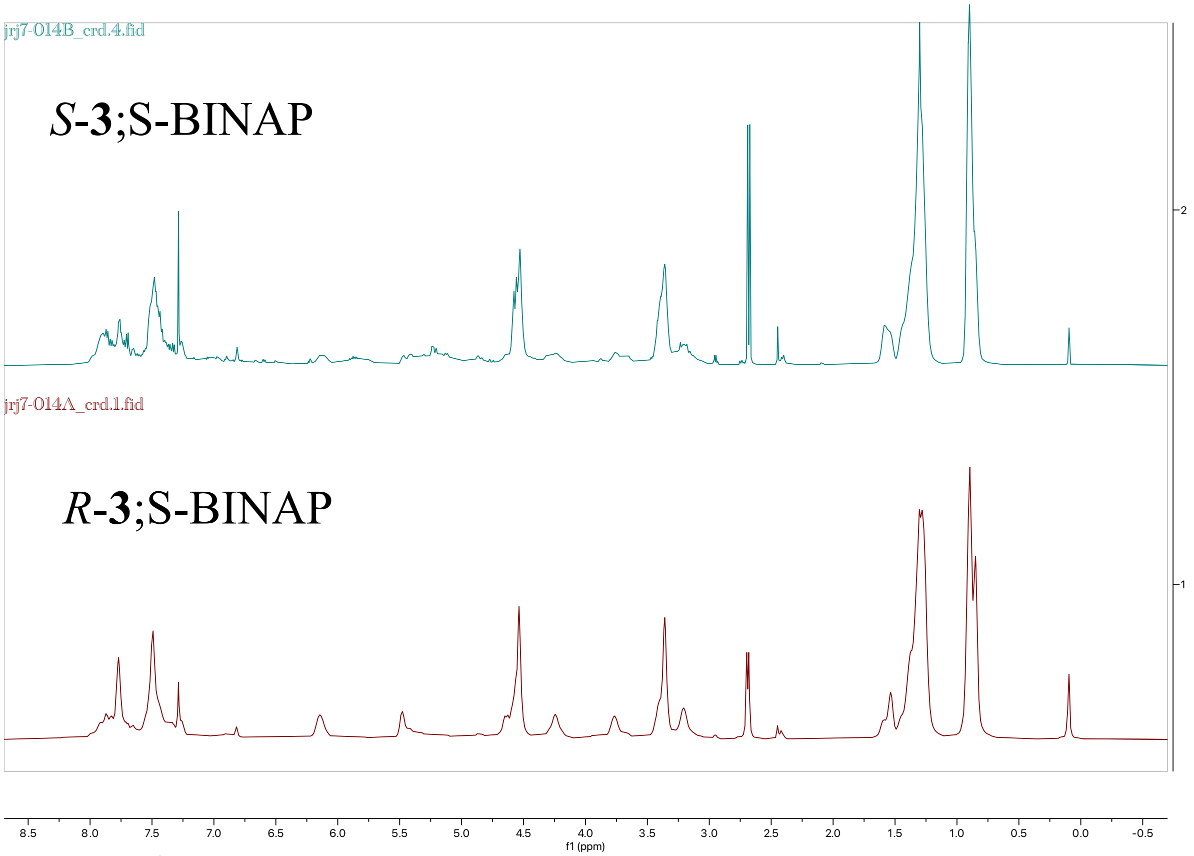

Figure S64. ^{1}H NMR spectra in $CDCl_3$ of poly(**3**) synthesized with both enantiomers of **3**.

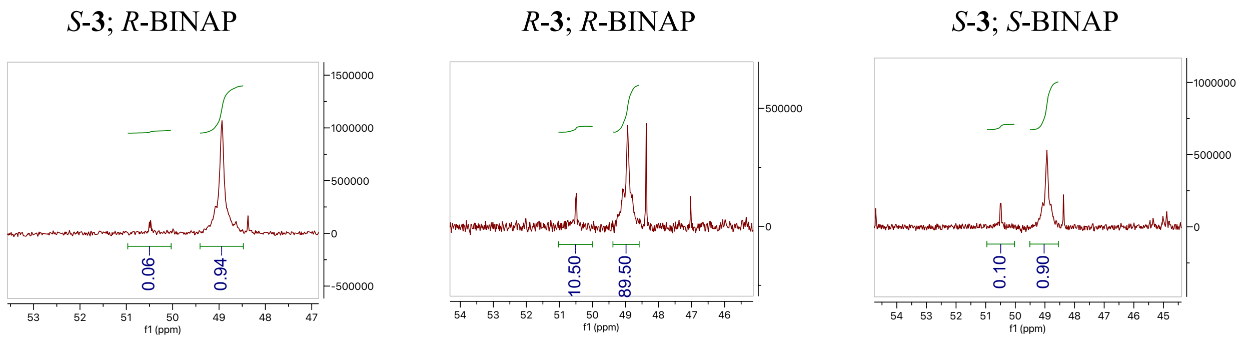

Figure S65. Tacticity calculations of poly(**3**)s synthesized in this work using enantioenriched **3**.

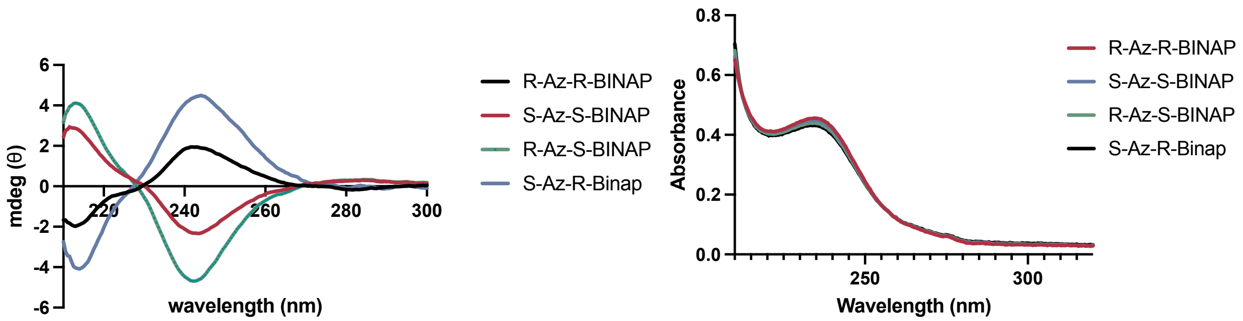

Figure S66. CD of polymers derived from enantioenriched monomers.

Table S8. Optical Rotation data for poly(**3**) derived from enantioenriched monomers.

Monomer enantiomer (*%ee*)	BINAP enantiomer	M_n	% m	α	*l* (dm)	*c* (g/mL)	[α]
R (95)	R	3.7	90	0.0058	1	0.00025	23.2
S (95)	S	5.5	94	-0.0069	1	0.00025	-27.6
R (95)	S	4.5	90	-0.0165	1	0.00025	-66

10. References

(1) Bakkali-Hassani, C.; Rieger, E.; Vignolle, J.; Wurm, F. R.; Carlotti, S.; Taton, D. The Organocatalytic Ring-Opening Polymerization of N-Tosyl Aziridines by an N-Heterocyclic Carbene. *Chemical Communications* 2016, *52* (62), 9719–9722. https://doi.org/10.1039/C6CC04323B.

(2) Salvado, O.; Gava, R.; Fernández, E. Diborylalkyllithium Salts Trigger Regioselective Ring Opening of Vinyl Aziridines. *Org Lett* 2019, *21* (22), 9247–9250. https://doi.org/10.1021/acs.orglett.9b03672.

(3) Guillon, R.; Pagniez, F.; Giraud, F.; Crépin, D.; Picot, C.; Le Borgne, M.; Morio, F.; Duflos, M.; Logé, C.; Le Pape, P. Design, Synthesis, and in Vitro Antifungal Activity of 1-[(4-Substituted-Benzyl)Methylamino]-2-(2,4-Difluorophenyl)-3-(1H-1,2,4-Triazol-1-Yl)Propan-2-Ols. *ChemMedChem* 2011, *6* (5), 816–825. https://doi.org/https://doi.org/10.1002/cmdc.201000530.

(4) Sirkecioglu, O.; Karliga, B.; Talinli, N. Benzylation of Alcohols by Using Bis[Acetylacetonato]Copper as Catalyst. *Tetrahedron Lett* 2003, *44* (46), 8483–8485. https://doi.org/https://doi.org/10.1016/j.tetlet.2003.09.106.

(5) Dauban, P.; Sanière, L.; Tarrade, A.; Dodd, R. H. Copper-Catalyzed Nitrogen Transfer Mediated by Iodosylbenzene PhI=O [2]. *Journal of the American Chemical Society*. 2001, pp 7707–7708. https://doi.org/10.1021/ja010968a.

(6) Ghorai, M. K.; Kumar, A.; Tiwari, D. P. BF3·OEt2-Mediated Highly Regioselective SN2-Type Ring-Opening of N-Activated Aziridines and N-Activated Azetidines by Tetraalkylammonium Halides. *J Org Chem* 2010, *75* (1), 137–151. https://doi.org/10.1021/jo902244y.

(7) Wang, X.; Liu, Y.; Li, Z.; Wang, H.; Gebru, H.; Chen, S.; Zhu, H.; Wei, F.; Guo, K. Organocatalyzed Anionic Ring-Opening Polymerizations of N-Sulfonyl Aziridines with Organic Superbases. *ACS Macro Lett* 2017, *6* (12), 1331–1336. https://doi.org/10.1021/acsmacrolett.7b00775.

(8) Bakkali-Hassani, C.; Rieger, E.; Vignolle, J.; Wurm, F. R.; Carlotti, S.; Taton, D. The Organocatalytic Ring-Opening Polymerization of N-Tosyl Aziridines by an N-Heterocyclic Carbene. *Chemical Communications* 2016, *52* (62), 9719–9722. https://doi.org/10.1039/C6CC04323B.

(9) Jagannathan, J. R.; Ma, Y.; Curole, B. J.; Grayson, S. M.; Fenton, O. S.; Leibfarth, F. A. Regioselective Palladium-Catalyzed Chain-Growth Allylic Amination Polymerization of Vinyl Aziridines. *J Am Chem Soc* 2024, *146* (22), 15264–15274. https://doi.org/10.1021/jacs.4c02599.

(10) Suzuki, M.; Sawada, S.; Saegusa, T. New Ring-Opening Polymerization via a .Pi.-Allyl Complex. 1. Polymerization of Diethyl 2-Vinylcyclopropane-1,1-Dicarboxylate Catalyzed by a Palladium(0) Complex. *Macromolecules* 1989, *22* (3), 1505–1507. https://doi.org/10.1021/ma00193a091.

(11) van Haaren, R. J.; Zuidema, E.; Fraanje, J.; Goubitz, K.; Kamer, P. C. J.; van Leeuwen, P. W. N. M.; van Strijdonck, G. P. F. Synthesis and Characterisation of Bite Angle-Dependent (H1-Allyl)Rh and (H3-Allyl)Rh Complexes Bearing Diphosphine Ligands. Implications for Nucleophilic Substitution Reactions. *Comptes Rendus Chimie* 2002, *5* (5), 431–440. https://doi.org/https://doi.org/10.1016/S1631-0748(02)01406-6.

(12) Xu, W.-B.; Sun, M.; Shu, M.; Li, C. Rhodium-Catalyzed Regio- and Enantioselective Allylic Amination of Racemic 1,2-Disubstituted Allylic Phosphates. *J Am Chem Soc* 2021, *143* (22), 8255–8260. https://doi.org/10.1021/jacs.1c04016.
(13) Evans, P. A.; Nelson, J. D. Conservation of Absolute Configuration in the Acyclic Rhodium-Catalyzed Allylic Alkylation Reaction: Evidence for an Enyl (σ + π) Organorhodium Intermediate. *J Am Chem Soc* 1998, *120* (22), 5581–5582. https://doi.org/10.1021/ja980030q.
(14) Tsuji, J.; Minami, I.; Shimizu, I. Allyation of Carbonucleophiles with Allylic Carbonates under Neutral Conditions Catalyzed by Rhodium Complexes. *Tetrahedron Lett* 1984, *25* (45), 5157–5160. https://doi.org/https://doi.org/10.1016/S0040-4039(01)81551-3.
(15) Pregosin, P. S.; Salzmann, R. Structure and Dynamics of Chiral Allyl Complexes of Pd(II): NMR Spectroscopy and Enantioselective Allylic Alkylation. *Coord Chem Rev* 1996, *155*, 35–68. https://doi.org/https://doi.org/10.1016/S0010-8545(96)90176-9.
(16) Satyanarayana, T.; Abraham, S.; Kagan, H. B. Nonlinear Effects in Asymmetric Catalysis. *Angewandte Chemie International Edition* 2009, *48* (3), 456–494. https://doi.org/https://doi.org/10.1002/anie.200705241.
(17) Sorensen, C. C.; Leibfarth, F. A. Stereoselective Helix-Sense-Selective Cationic Polymerization of N-Vinylcarbazole Using Chiral Lewis Acid Catalysis. *J Am Chem Soc* 2022, *144* (19), 8487–8492. https://doi.org/10.1021/jacs.2c02738.

11. Characterization Data

NMR Small Molecules

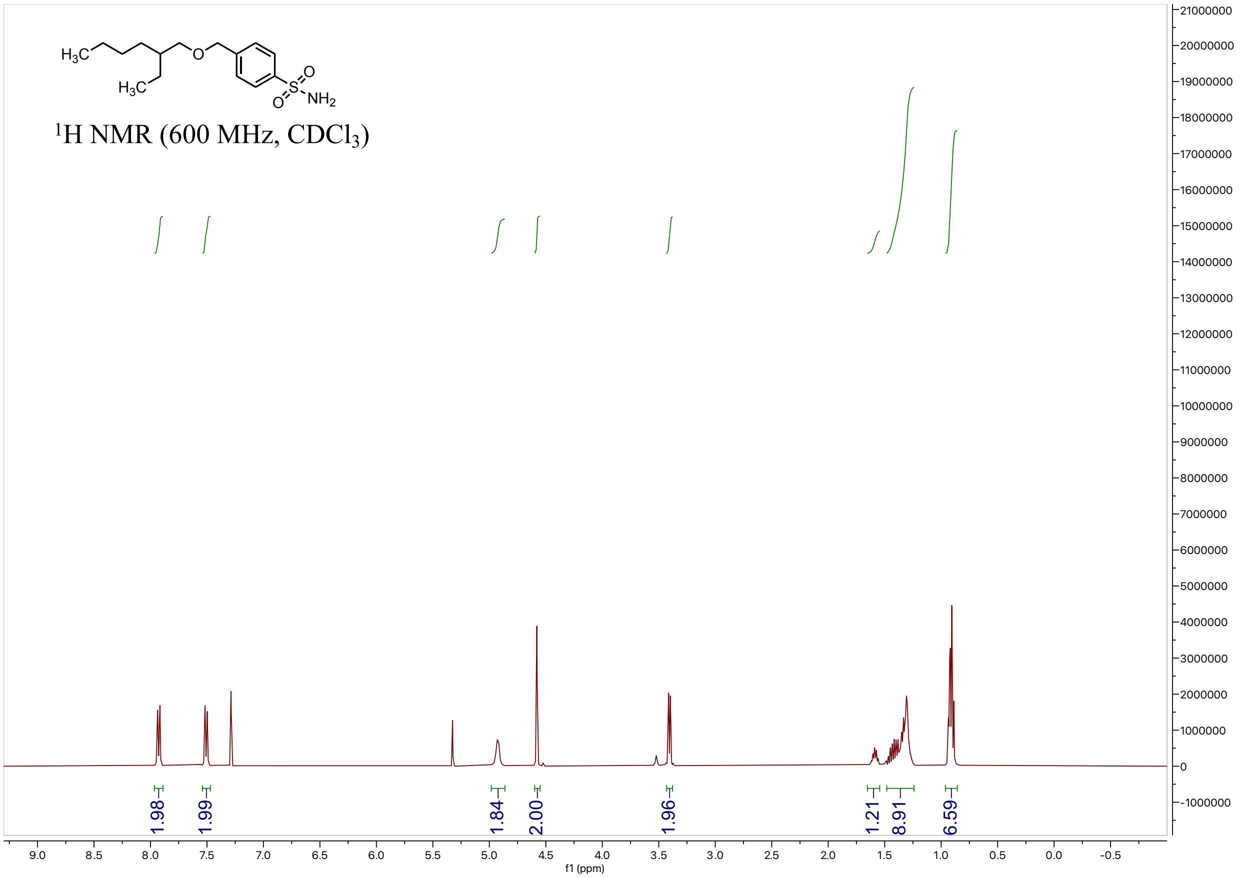

Figure S67. ^{1}H NMR spectrum of *rac*-4-(((2-ethylhexyl)oxy)methyl)benzenesulfonamide in $CDCl_3$.

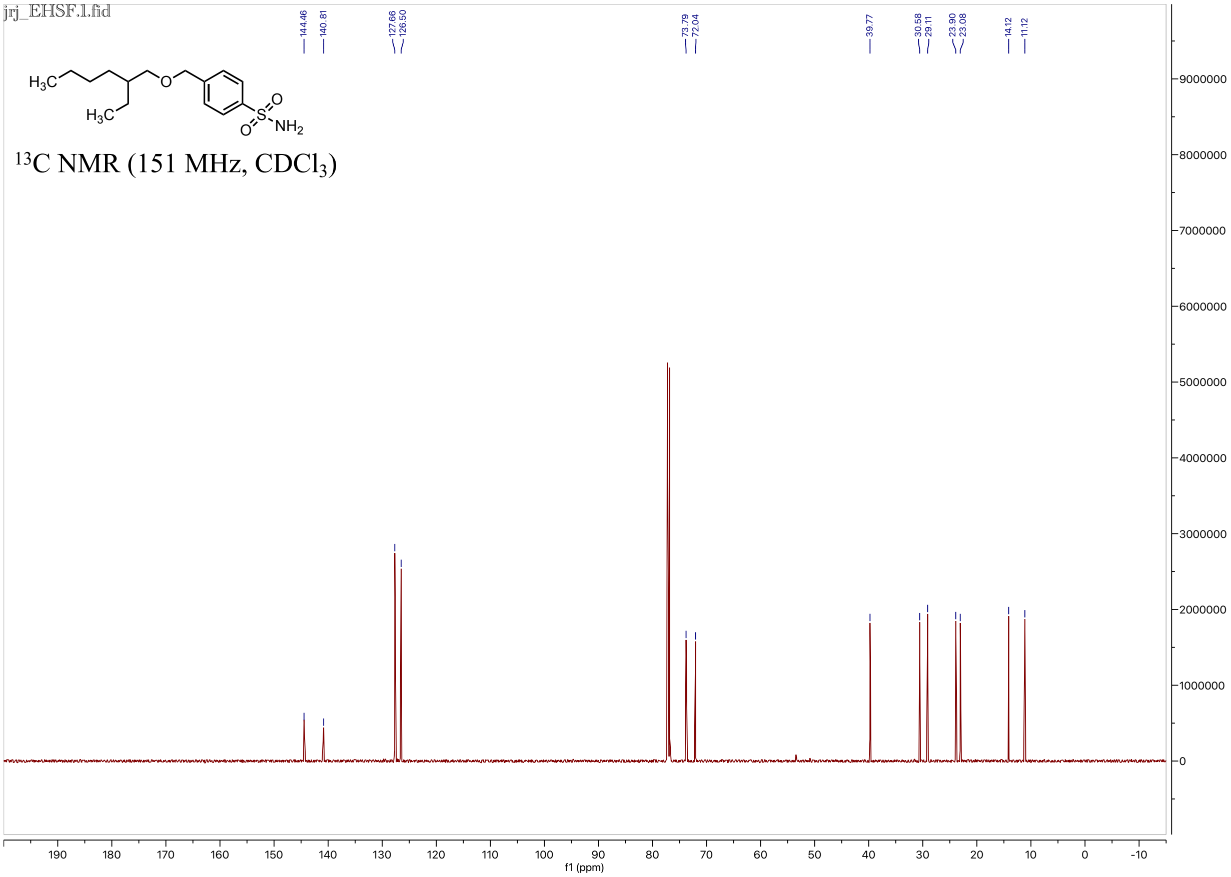

Figure S68. ^{13}C NMR spectrum of *rac*-4-(((2-ethylhexyl)oxy)methyl)benzenesulfonamide in CDCl$_3$.

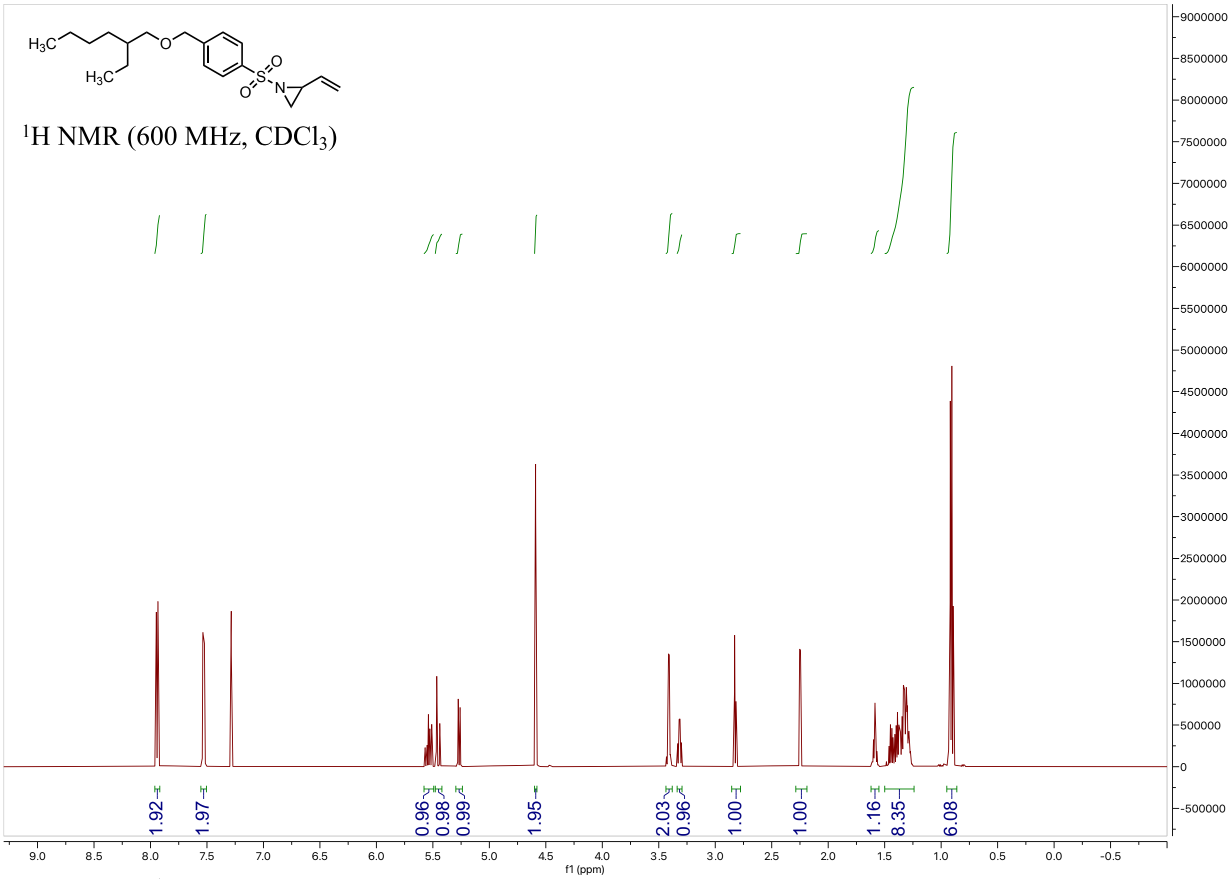

Figure S69. ^{1}H NMR spectrum of *rac*-1-((4-(((2-ethylhexyl)oxy)methyl)phenyl)sulfonyl)-2-vinylaziridine in $CDCl_3$.

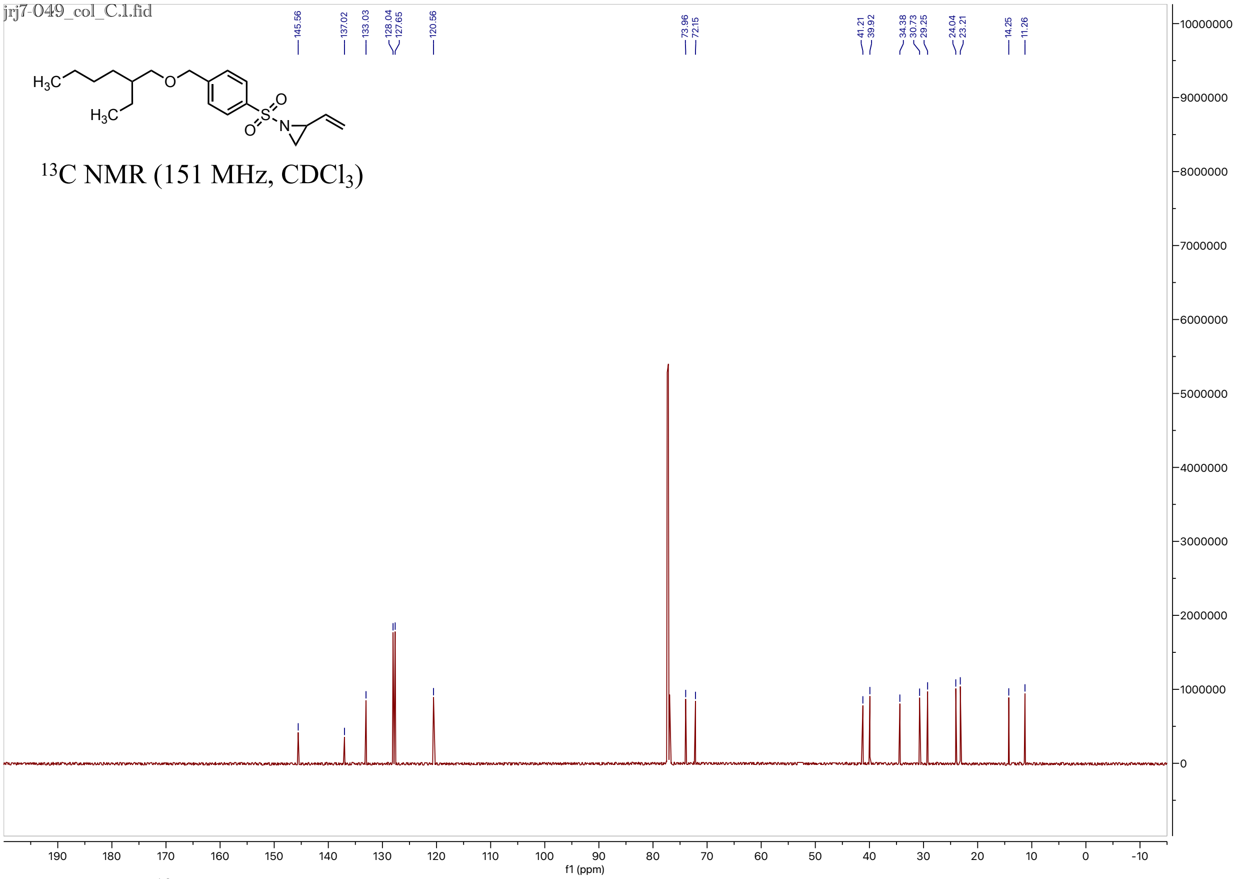

Figure S70. ^{13}C NMR spectrum of *rac*-1-((4-(((2-ethylhexyl)oxy)methyl)phenyl)sulfonyl)-2-vinylaziridine in $CDCl_3$.

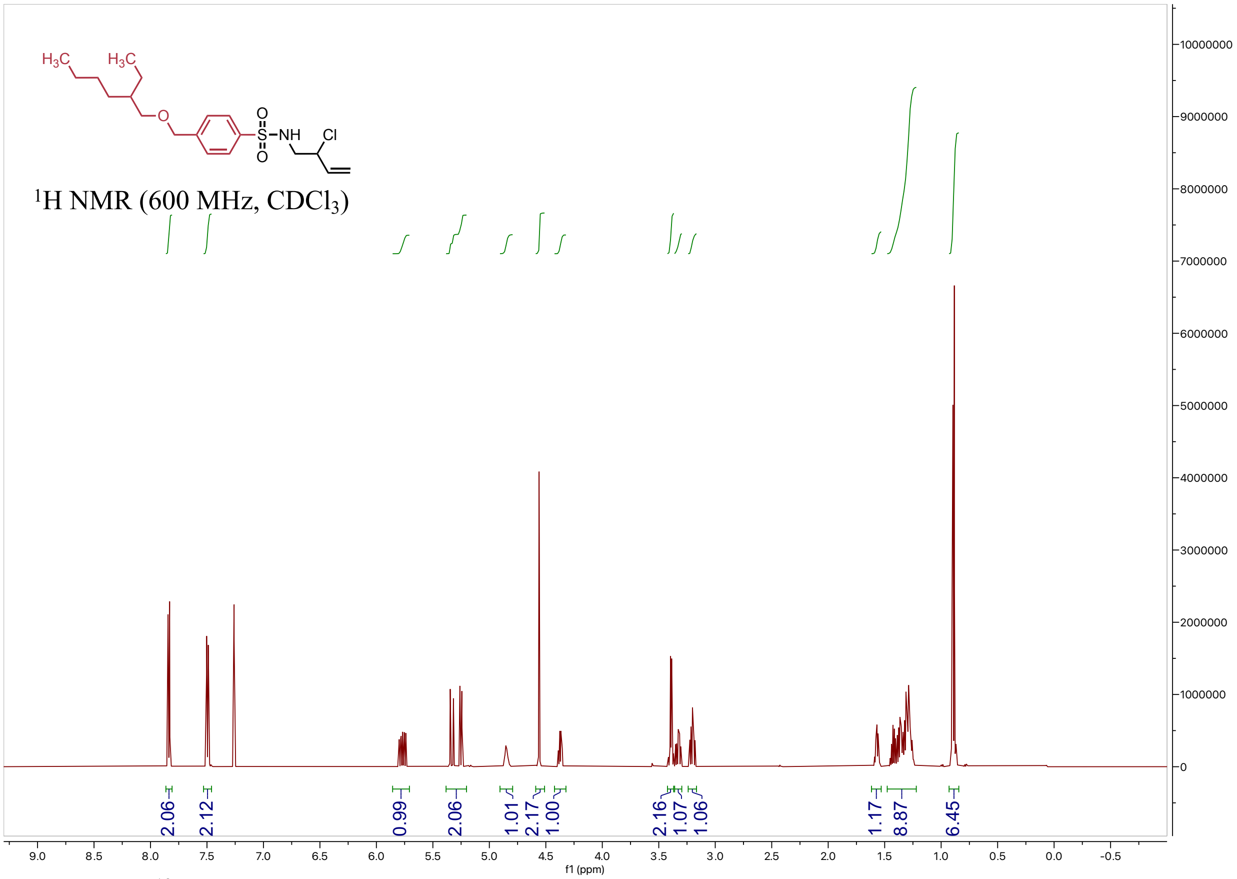

Figure S71. ^{13}C NMR spectrum of *rac*-1-((4-(((2-ethylhexyl)oxy)methyl)phenyl)sulfonyl)-2-vinylaziridine in $CDCl_3$.

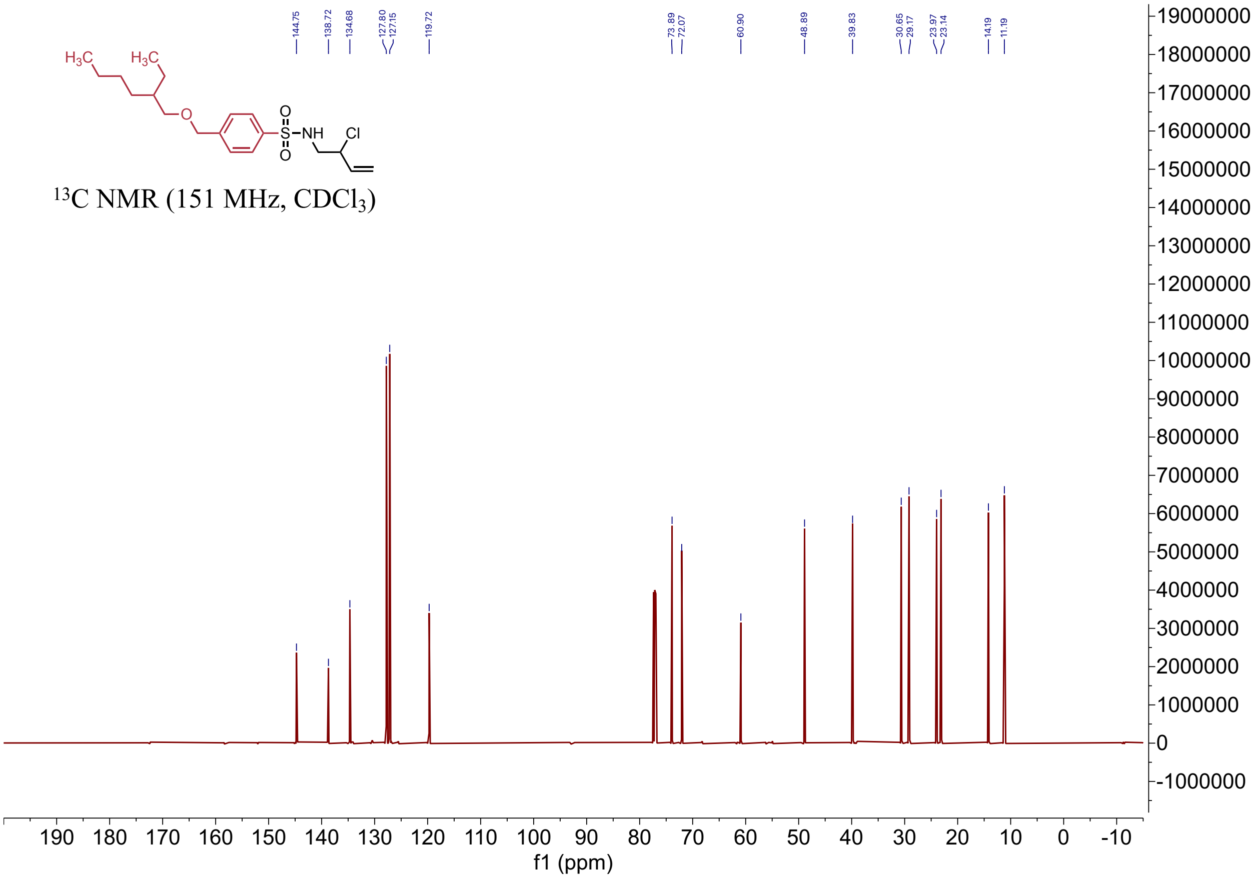

Figure S72. ^{13}C NMR spectrum of *rac*-1-((4-(((2-ethylhexyl)oxy)methyl)phenyl)sulfonyl)-2-vinylaziridine in $CDCl_3$.

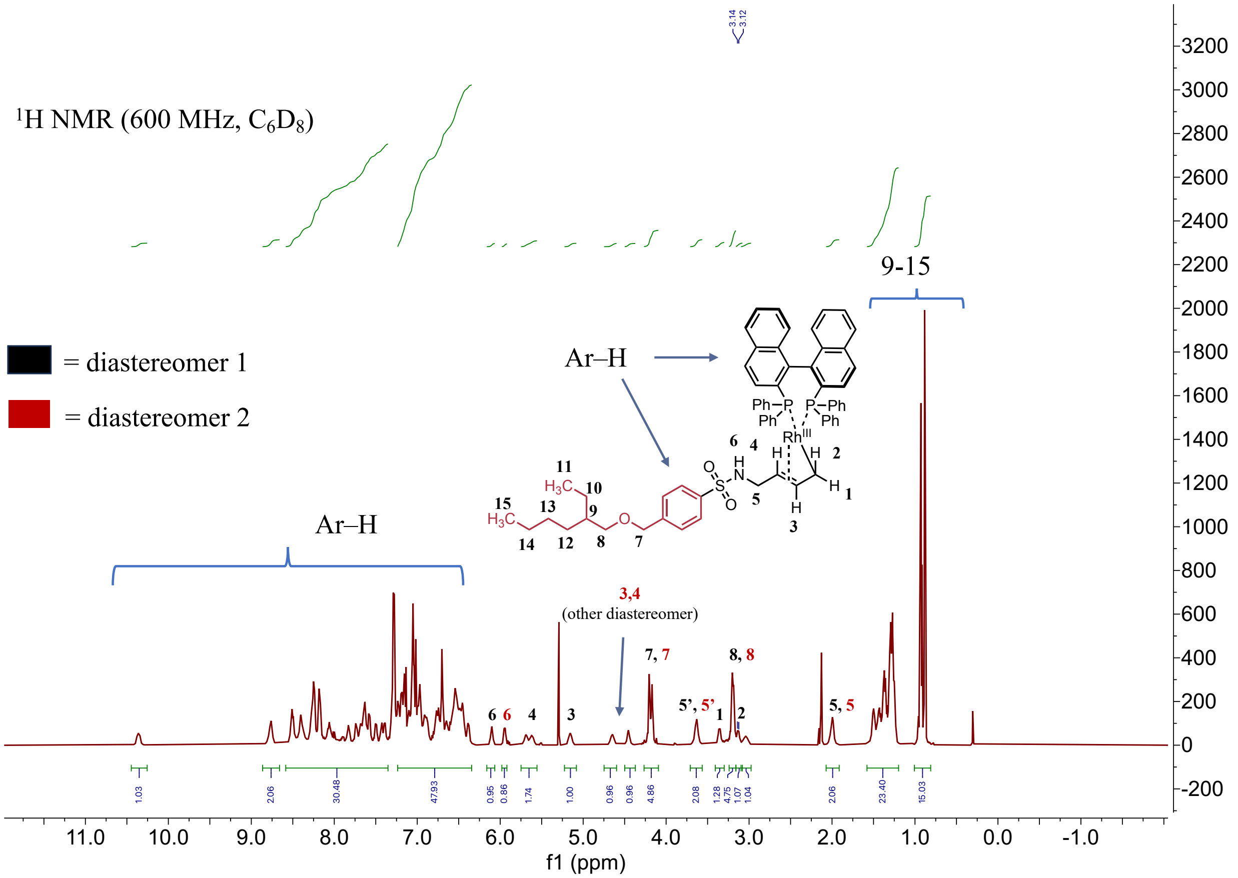

Figure S73. ^1H NMR spectrum of **5** in C_7D_8.

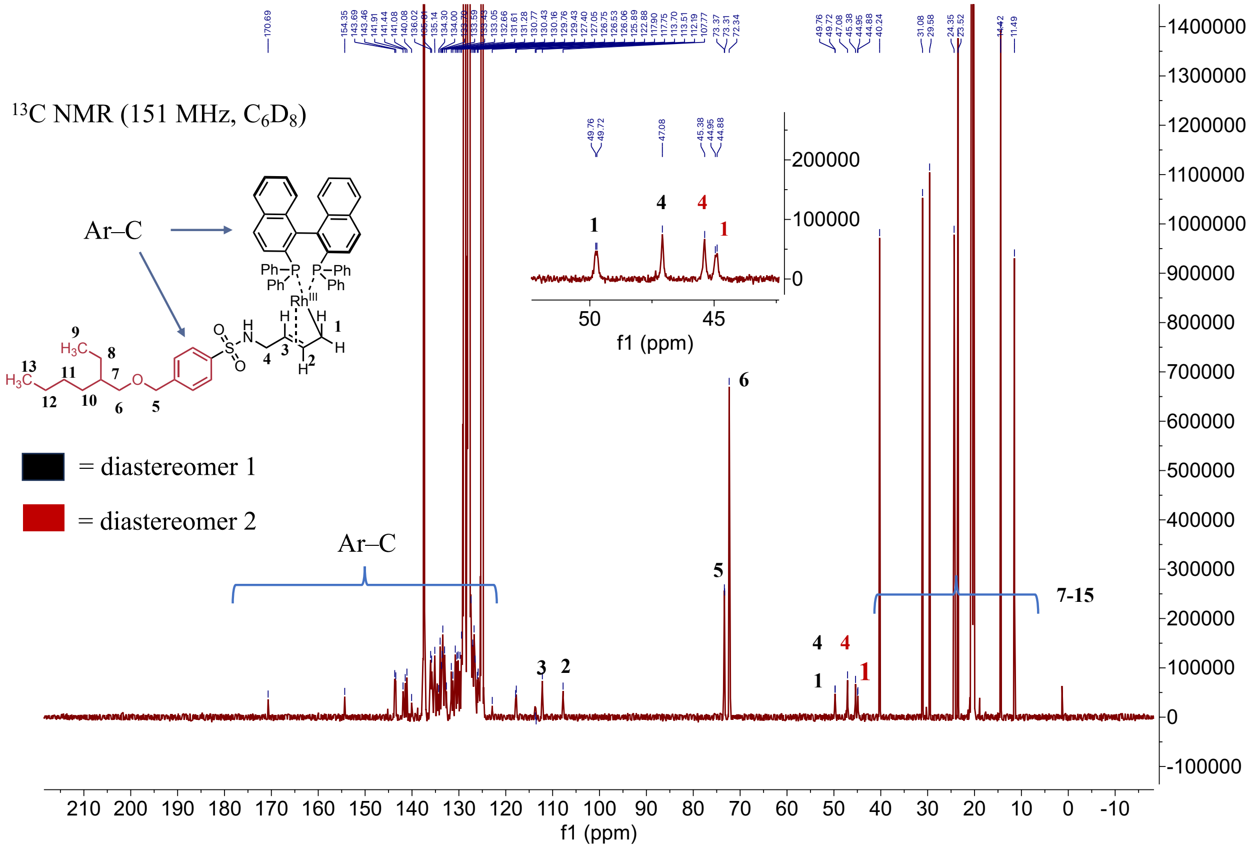

Figure S74. ^{13}C NMR spectrum of **5** in C_7D_8.

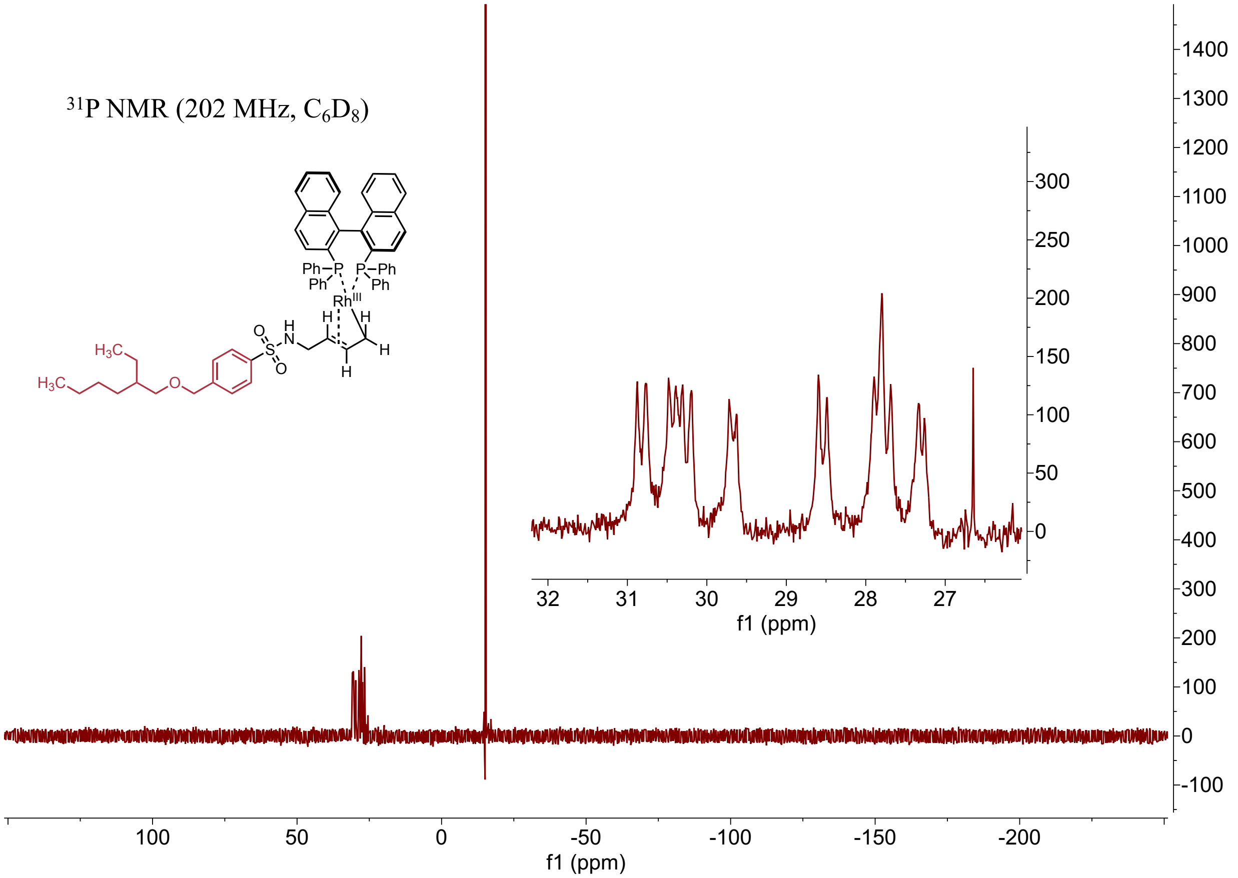

Figure S75. ^{31}P NMR spectrum of **5** in C_7D_8.

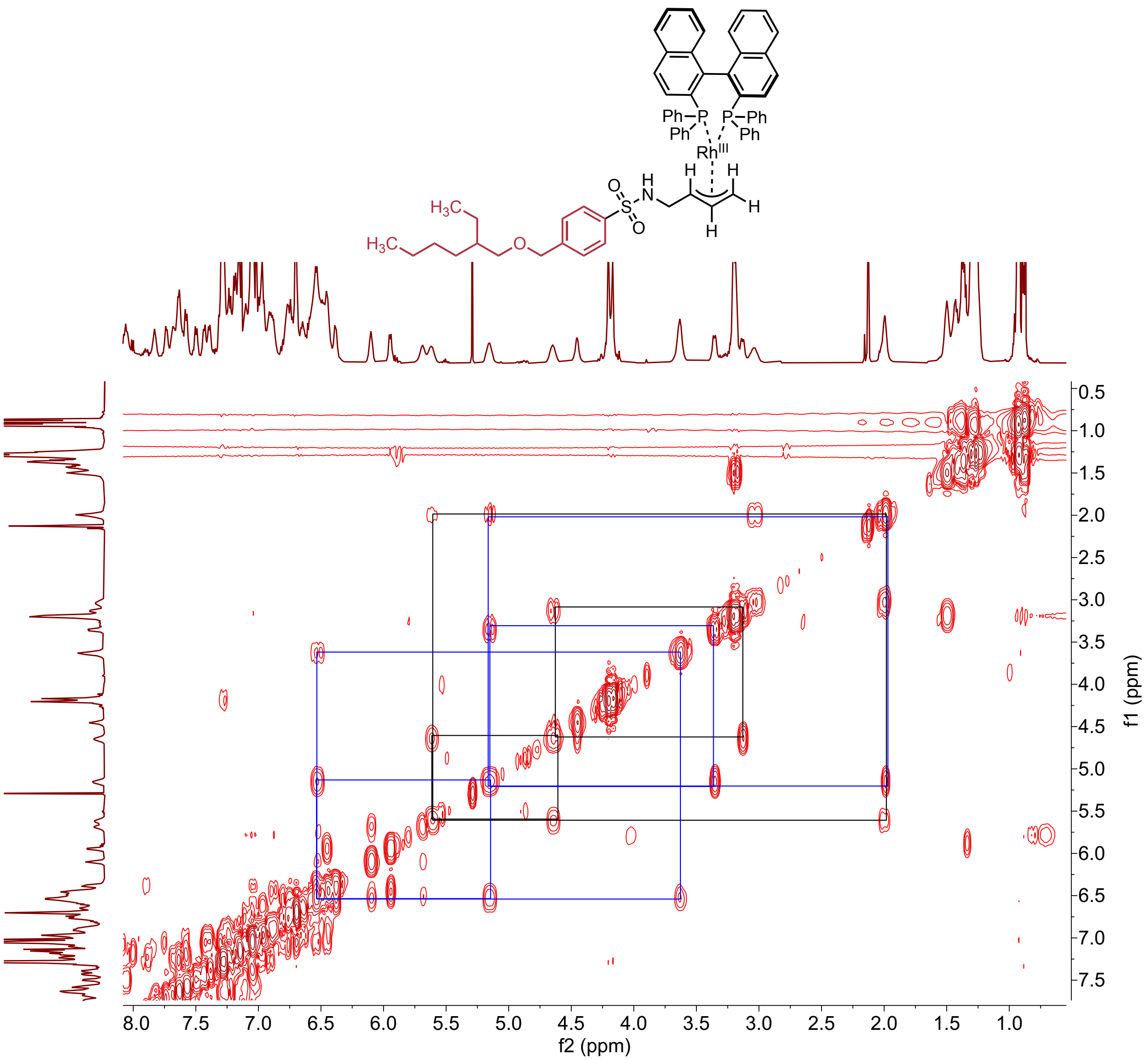

Figure S76. ^{1}H-^{1}H COSY spectrum of **5** in C_7D_8 and the observed spin systems for the diastereomers, denoted with black and blue squares.

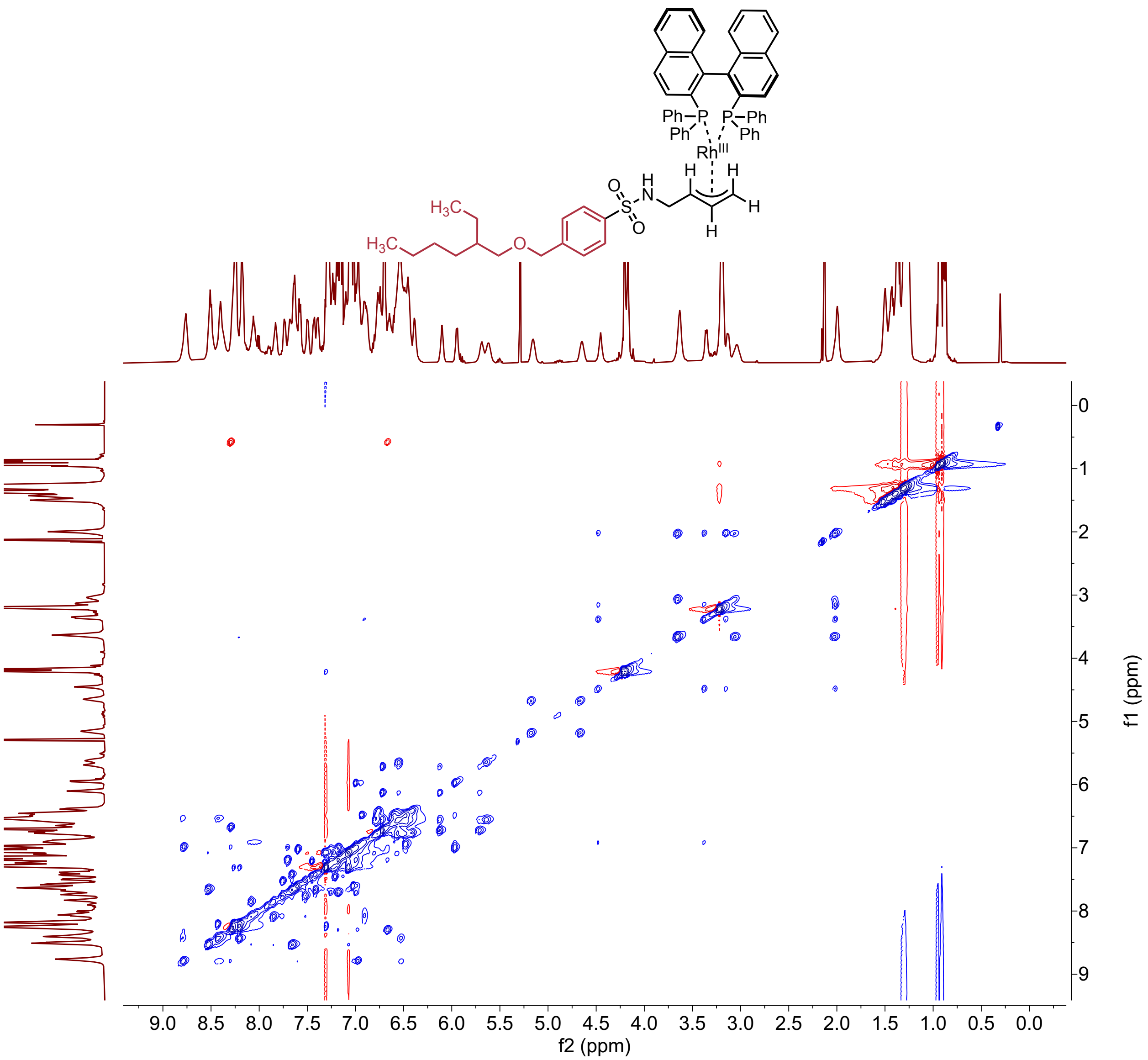

Figure S77. 2D NOESY spectrum of **5** in C_7D_8

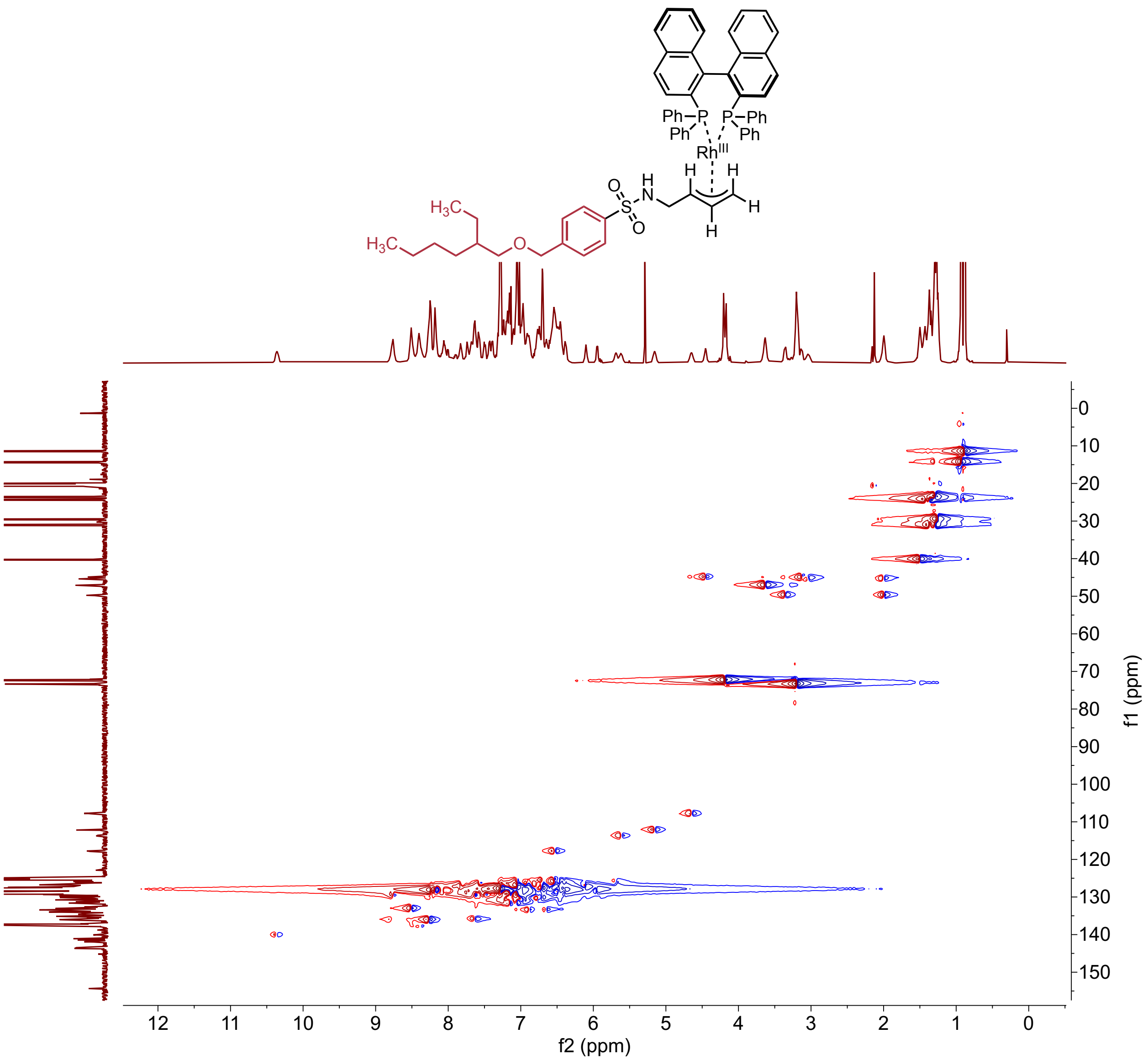

Figure S78. ^{1}H-^{13}C HSQC spectrum of **5** in C_7D_8

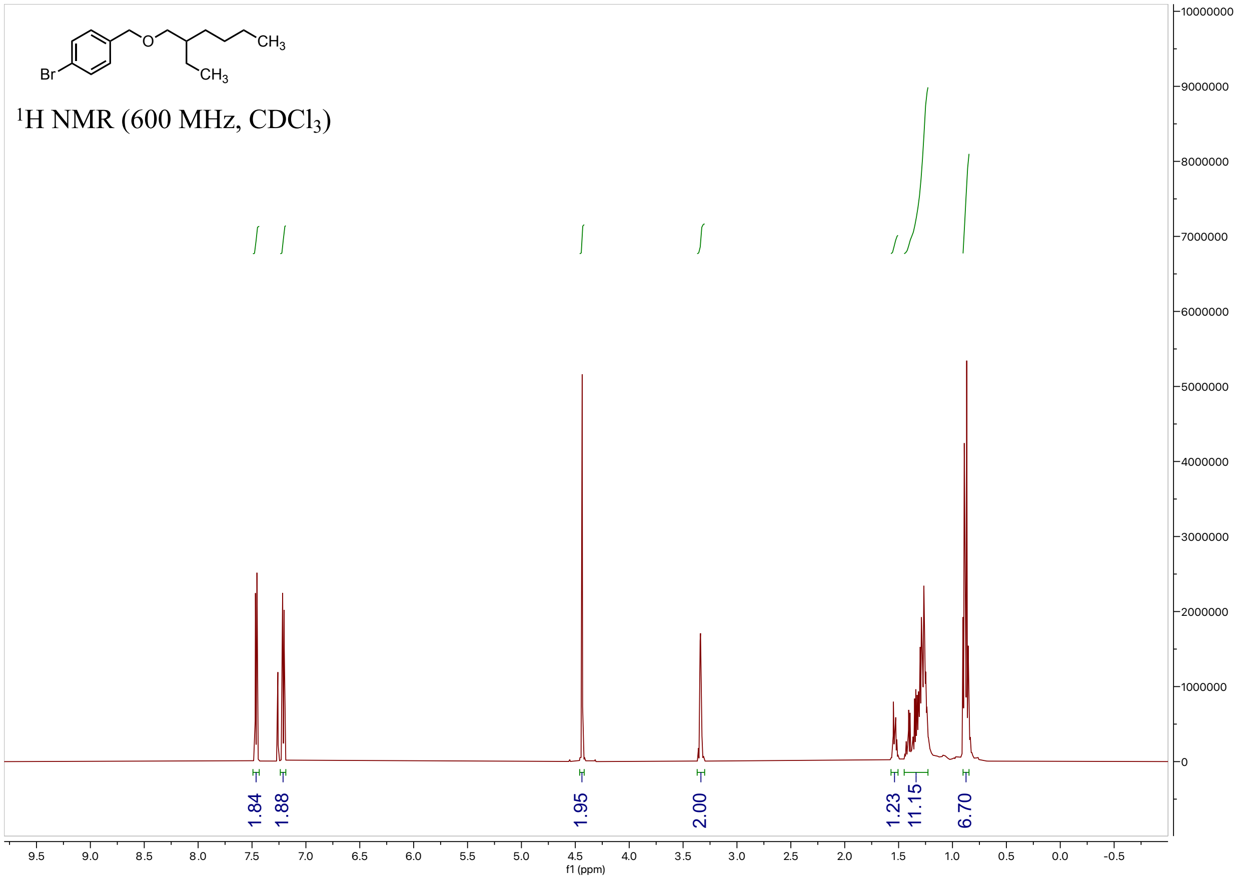

Figure S79. ^{1}H NMR spectrum of *rac*-1-bromo-4-(((2-ethylhexyl)oxy)methyl)benzene in $CDCl_3$.

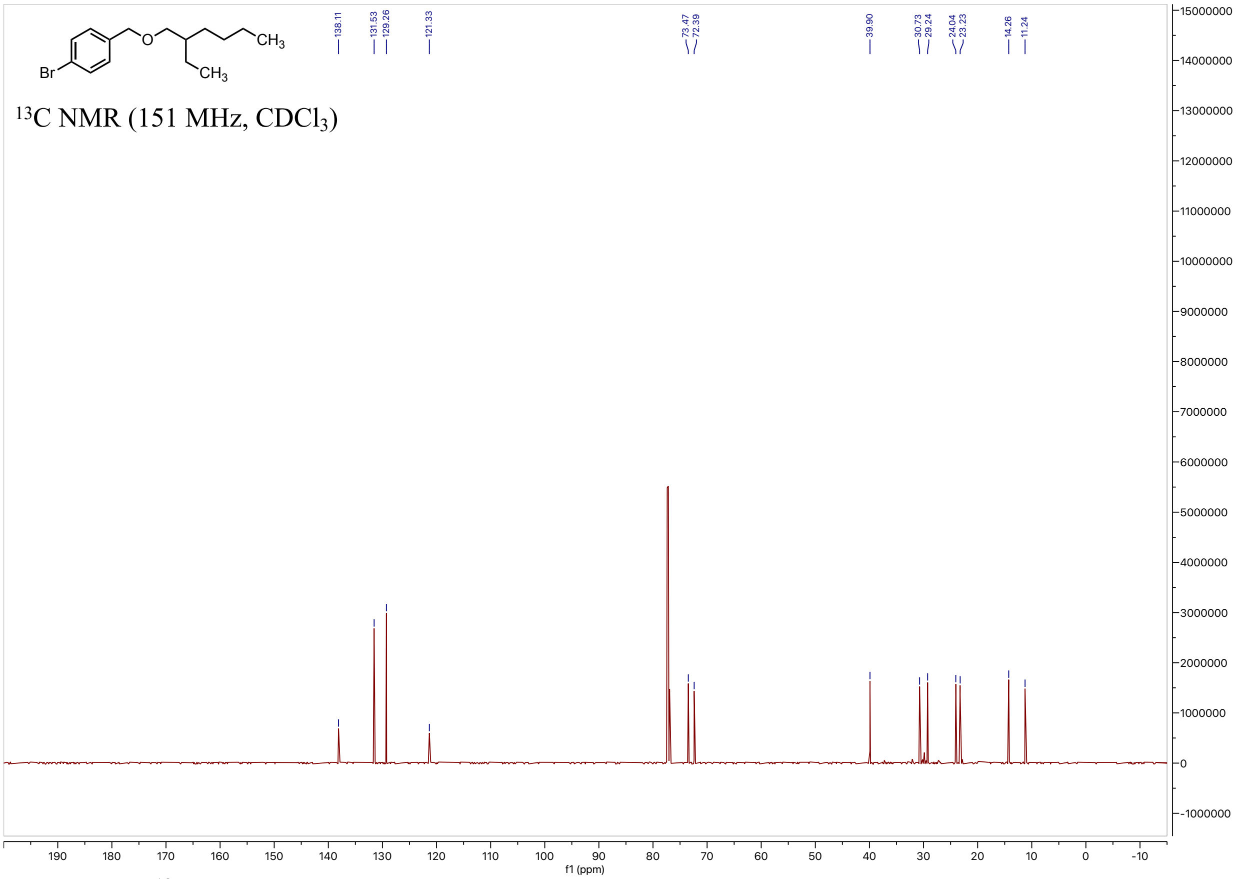

Figure S80. ^{13}C NMR spectrum of *rac*-1-bromo-4-(((2-ethylhexyl)oxy)methyl)benzene in $CDCl_3$.

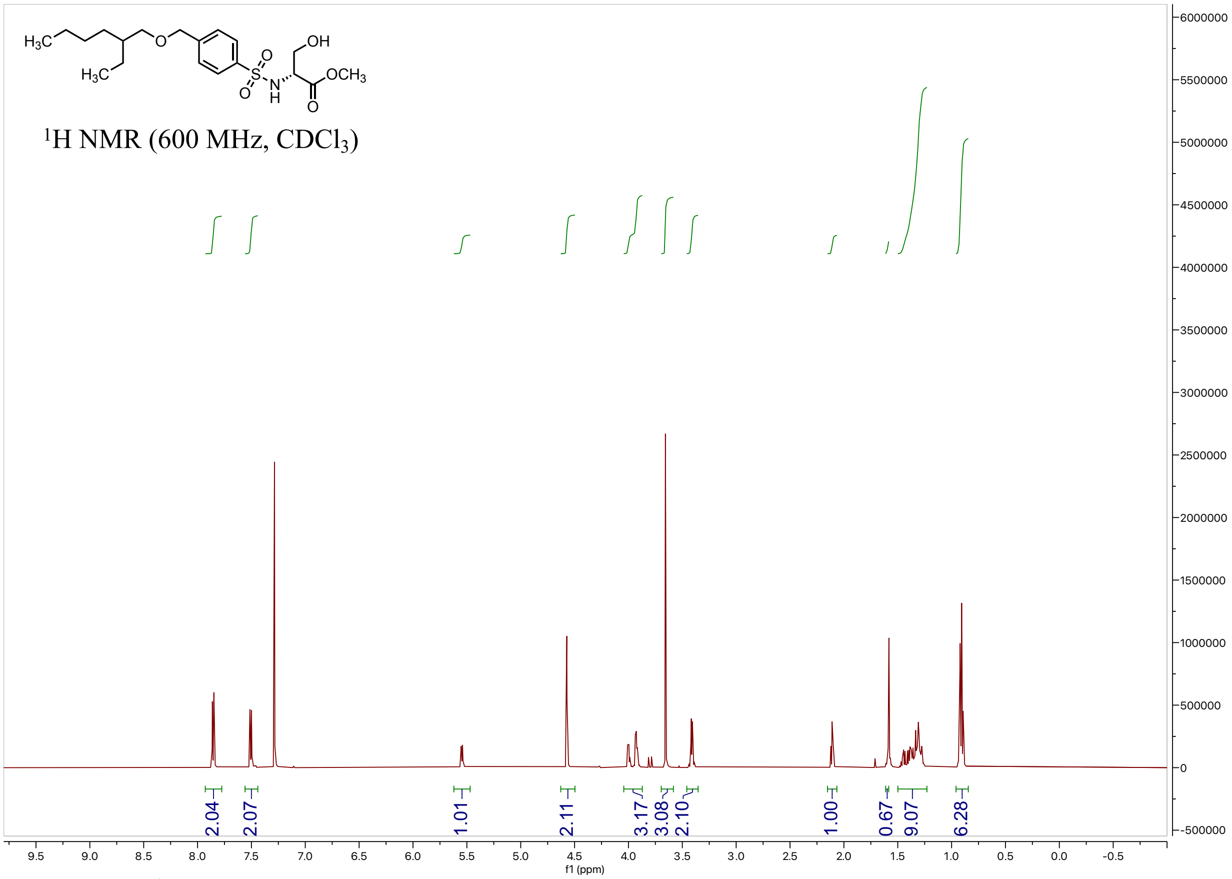

Figure S81. ^{1}H NMR spectrum of methyl ((4-(((2-ethylhexyl)oxy)methyl)phenyl)sulfonyl)-*D*-serinate in $CDCl_3$.

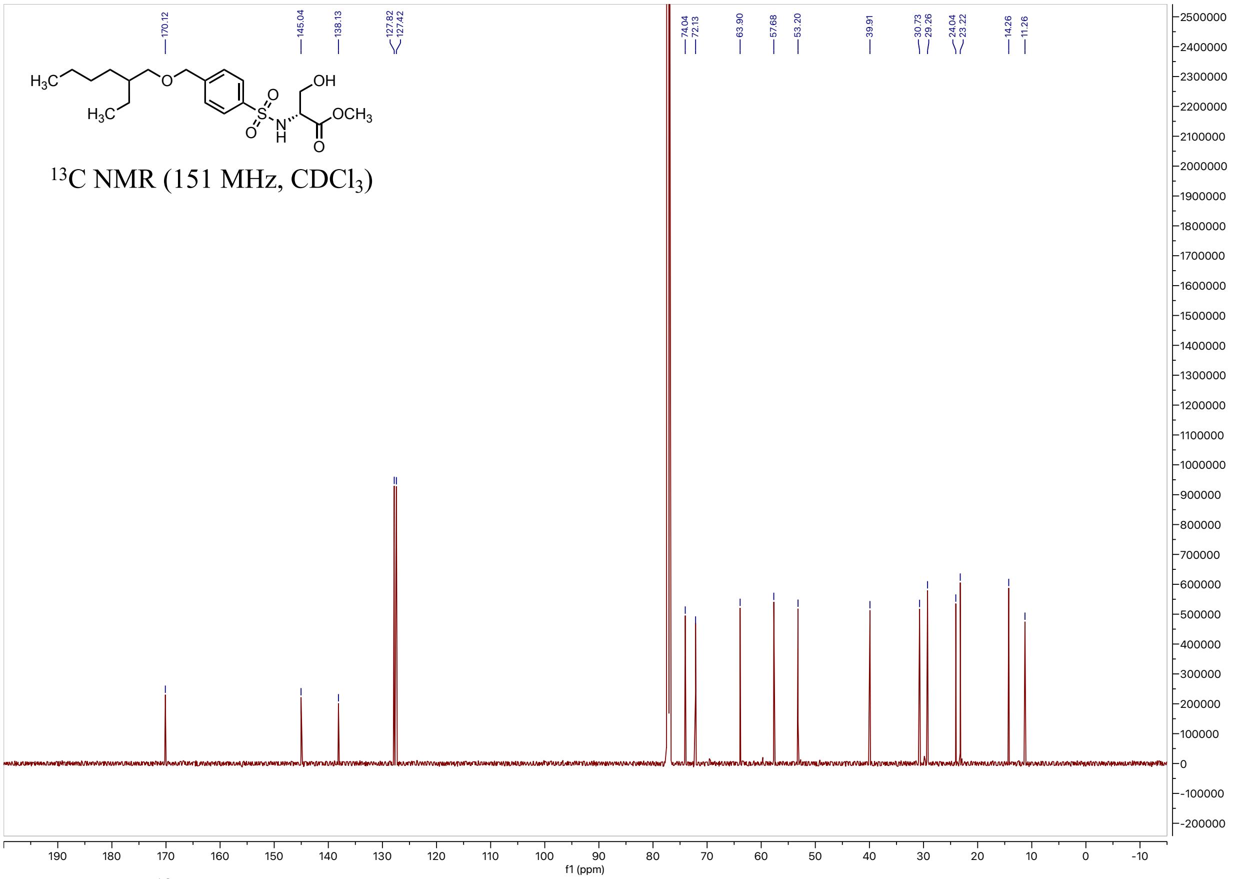

Figure S82. ^{13}C NMR spectrum of methyl ((4-(((2-ethylhexyl)oxy)methyl)phenyl)sulfonyl)-*D*-serinate in $CDCl_3$.

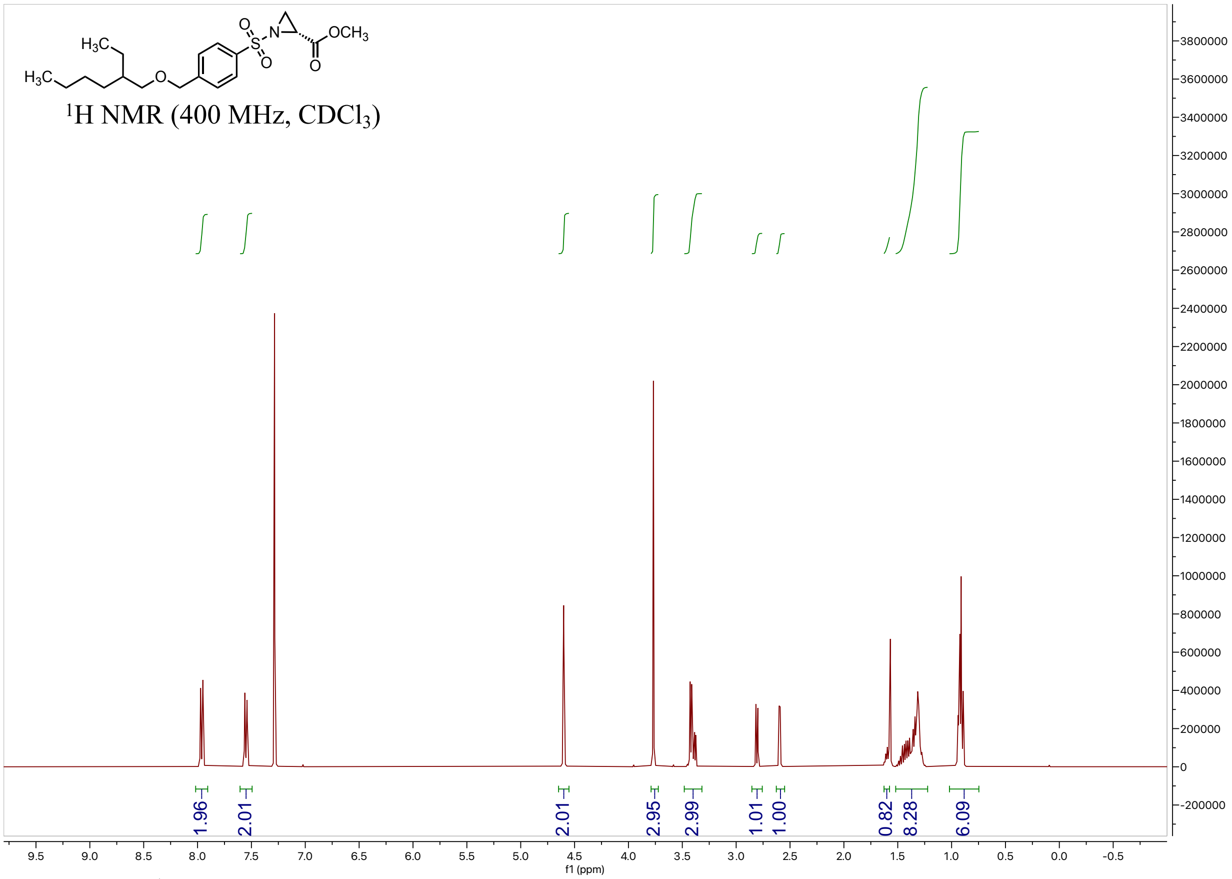

Figure S83. ^{1}H NMR spectrum of methyl (2*R*)-1-((4-(((2-ethylhexyl)oxy)methyl)phenyl) sulfonyl)aziridine-2-carboxylate in $CDCl_3$.

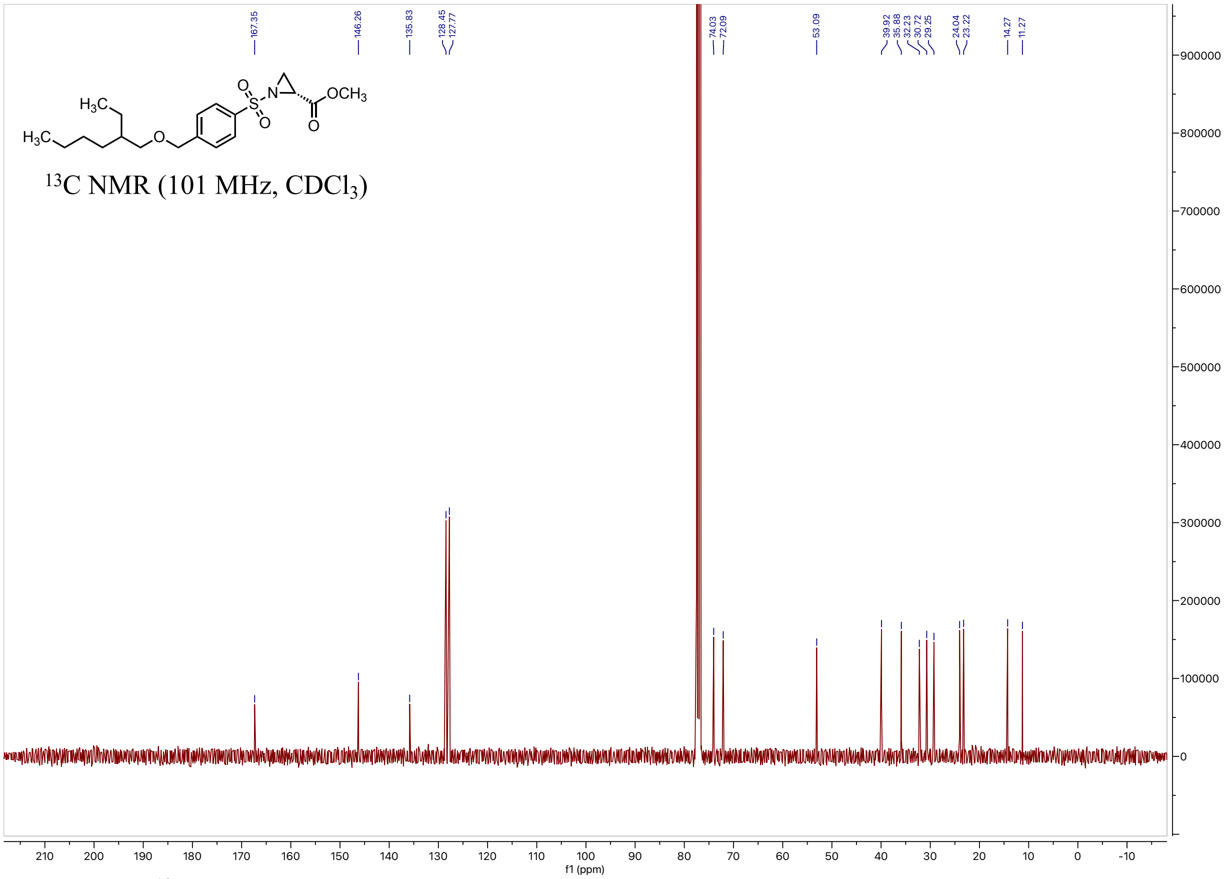

Figure S84. ^{13}C NMR spectrum of methyl (2*R*)-1-((4-(((2-ethylhexyl)oxy)methyl)phenyl) sulfonyl) aziridine-2-carboxylate in $CDCl_3$.
